# Altered lipid metabolism and inflammatory programs associate with adipocyte loss in familial partial lipodystrophy 2

**DOI:** 10.1172/JCI198387

**Published:** 2025-11-11

**Authors:** Jessica N. Maung, Rebecca L. Schill, Akira Nishii, Maria Foss de Freitas, Bonje N. Obua, Marcus Nygård, Maria D. Mendez-Casillas, Isabel D.K. Hermsmeyer, Donatella Gilio, Ozge Besci, Yang Chen, Brian Desrosiers, Rose E. Adler, Anabela D. Gomes, Merve Celik Guler, Hiroyuki Mori, Romina M. Uranga, Ziru Li, Hadla Hariri, Liping Zhang, Anderson de Paula Souza, Keegan S. Hoose, Kenneth T. Lewis, Taryn A. Hetrick, Paul Cederna, Carey N. Lumeng, Susanne Mandrup, Elif A. Oral, Ormond A. MacDougald

**Affiliations:** 1 Department of Molecular & Integrative Physiology,; 2 Department of Internal Medicine, and; 3 Cellular and Molecular Biology Program, University of Michigan Medical School, Ann Arbor, Michigan, USA.; 4 Center for Functional Genomics and Tissue Plasticity (ATLAS), Department of Biochemistry and Molecular Biology, University of Southern Denmark (SDU), Odense, Denmark.; 5 Department of Surgery, University of Michigan Medical School, Ann Arbor, Michigan, USA.

**Keywords:** Clinical Research, Metabolism, Adipose tissue, Bioinformatics, Genetic diseases

## Abstract

Familial partial lipodystrophy 2 (FPLD2) is a rare disease characterized by adipose tissue loss and redistribution and metabolic dysfunction. FPLD2 is caused by pathogenic variants in the *LMNA* gene, encoding nuclear lamins A/C, structural proteins that control nuclear function and gene expression. However, the mechanisms driving adipocyte loss in FPLD2 remain poorly defined. In this study, we recruited 8 families with developing or established FPLD2 and performed clinical, histological, and transcriptomic analyses of subcutaneous adipose tissue biopsies. Bulk and single-nucleus RNA sequencing revealed suppression of lipid metabolism and mitochondrial pathways, alongside increased inflammation. These signatures were mirrored in tamoxifen-inducible adipocyte-specific *Lmna*-knockout mice, in which lamin A/C-deficient adipocytes shrank and disappeared. *Lmna-*deficient fibroblasts shared similar gene expression changes, linked to altered chromatin accessibility, underscoring lamin A/C’s potential regulatory role in lipid metabolism and inflammatory programs. By directly comparing atrophic and hypertrophic adipose depots in FPLD2, and integrating human, mouse, and in vitro models, this study provides insights into disease progression and potential therapeutic targets.

## Abstract 2

Familial partial lipodystrophy 2, caused by mutations in lamin A/C, leads to fat loss. Lamin A/C controls lipid metabolism, inflammation, and adipocyte maintenance.

## Graphical abstract

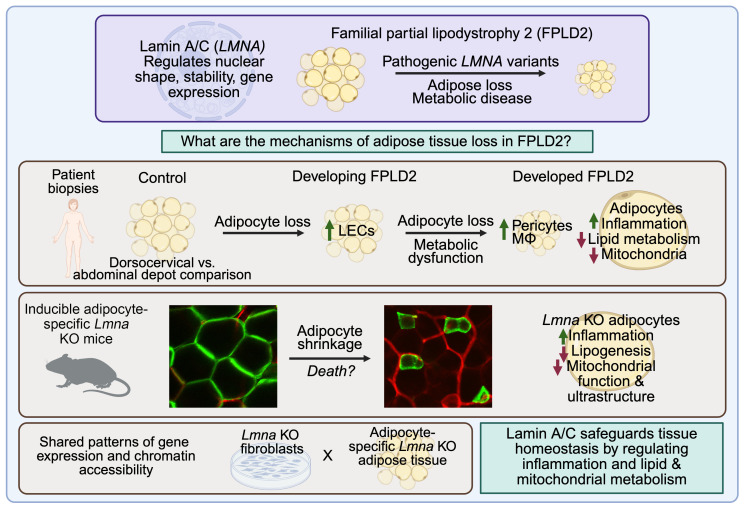

## Introduction

Adipose tissue is a key metabolic and endocrine organ, and alterations in its mass or function have major health consequences. Whereas excess adipose tissue contributes to obesity and its comorbidities, lipodystrophy syndromes involve adipose loss, redistribution, and dysfunction. Despite their contrasting phenotypes, both conditions cause metabolic complications such as fatty liver, insulin resistance, and cardiovascular disease because of loss of safe lipid storage (1, 2). Initially estimated to affect ~1 in 1 million (3), lipodystrophy prevalence is now thought to be up to ~1 in 20,000, with genetic prevalence near ~1 in 7,000 (4). The most common form, familial partial lipodystrophy type 2 (FPLD2, Dunnigan’s disease), features selective subcutaneous fat loss from limbs and trunk with redistribution to the face, neck, and sometimes visceral depots (1, 5–7). Treatments include lifestyle interventions; management of hyperlipidemia and insulin resistance, including with glucagon-like peptide-1 (GLP-1) receptor agonists (8, 9); and recombinant leptin, which improves multiple metabolic abnormalities (10). However, mechanisms driving adipose loss in FPLD2 remain unclear, and defining them could identify new therapeutic targets.

FPLD2 arises from pathogenic *LMNA* missense variants encoding lamin A/C (11), key intermediate filament proteins forming the nuclear lamina. This structure supports the nucleus, regulates nuclear transport and mechanical responses, and organizes chromatin via lamina-associated domains (12–14). Mutations disrupting lamin A/C cause diverse laminopathies, including muscular dystrophies, cardiomyopathies, neuropathies, and premature aging syndromes. In FPLD2, substitutions of arginine 482 (R482Q/W/L) disrupt a DNA-binding region, likely leading to transcriptional dysregulation (15–18). Thus, dissecting FPLD2 mechanisms may reveal broader principles of tissue-specific disease in laminopathies.

Fundamental studies of *Lmna*-knockout (-KO) mouse embryonic fibroblasts (MEFs) show abnormal nuclear morphology, altered nuclear pore complex distribution, and reduced cellular stiffness (19, 20). In mice, global *Lmna* deletion causes muscular dystrophy and premature death, limiting studies of metabolic tissues like adipose (19, 21). Overexpression of FPLD2-associated *LMNA* R482Q/W in 3T3-L1 preadipocytes inhibits adipocyte differentiation, as does overexpression of wild-type lamin A/C (22), suggesting that disruption of lamin stoichiometry can impair adipogenesis. Transgenic mice overexpressing human R482Q lamin A under the aP2 promoter either lacked overt lipodystrophy (23) or developed mild adipose loss after prolonged high-fat diet (24). These mice exhibited increased extracellular matrix (ECM) and fibrosis in white adipose tissue (WAT), even without lipodystrophy, implicating *Lmna* variants in adipose ECM remodeling (23). However, overexpression models and the aP2 promoter’s macrophage activity (25) complicate interpretation, underscoring the need for better adipocyte-specific models. Beyond cell culture and mouse models, studies of human WAT in FPLD2 are rare because of limited tissue availability. Analyses of abdominal and thigh adipose tissue revealed downregulated lipid metabolism genes and heterochromatin defects, while dorsocervical fat, often hypertrophic in FPLD2, showed heterogeneous adipocyte morphology, fibrosis, and brown adipose–like features (23, 26, 27). These findings suggest complex adipose remodeling in FPLD2, but underlying mechanisms remain incompletely defined.

Our group previously developed an adipocyte-specific *Lmna*-KO (*Lmna*^ADKO^) mouse recapitulating FPLD2 features, including progressive WAT loss and metabolic dysfunction (28). *Lmna*^ADKO^ mice failed to exhibit the normal diurnal oscillation in respiratory exchange ratio and thus were metabolically inflexible, whereas patients with FPLD2 exhibit increased energy expenditure and fat oxidation only upon dietary fat challenge but not at baseline (29). *Lmna*^ADKO^ mice exhibited smaller adipocytes without macrophage infiltration, suggesting cell-autonomous mechanisms of adipocyte loss. Although lacking a pathogenic *LMNA* variant, the model verified the essential role of lamin A/C in adipocyte maintenance.

In this study, we recruited 8 families with confirmed *LMNA* variants and obtained abdominal and upper neck subcutaneous biopsies for histologic and molecular WAT profiling. With 16 participants, this represents the largest FPLD2 biopsy cohort to profile adipose transcriptomes at single-cell resolution. We also developed a tamoxifen-inducible adipocyte-specific *Lmna*-KO (*Lmna*^iADKO^) model to trace adipocyte loss dynamics in adult mice. Integration of human and mouse data revealed conserved suppression of lipid metabolism and activation of inflammatory pathways contributing to adipocyte degeneration. Combining these findings with chromatin accessibility analyses from *Lmna*-KO MEFs demonstrated that lamin A/C deficiency alters accessibility of lipid and immune regulatory genes. Taken together, these results define shared molecular signatures upon loss or dysfunction of lamin A/C and highlight pathways of therapeutic relevance for treatment in FPLD2.

## Results

### Participants with FPLD2 display loss of adipose tissue and metabolic dysfunction.

**Figure 1:**
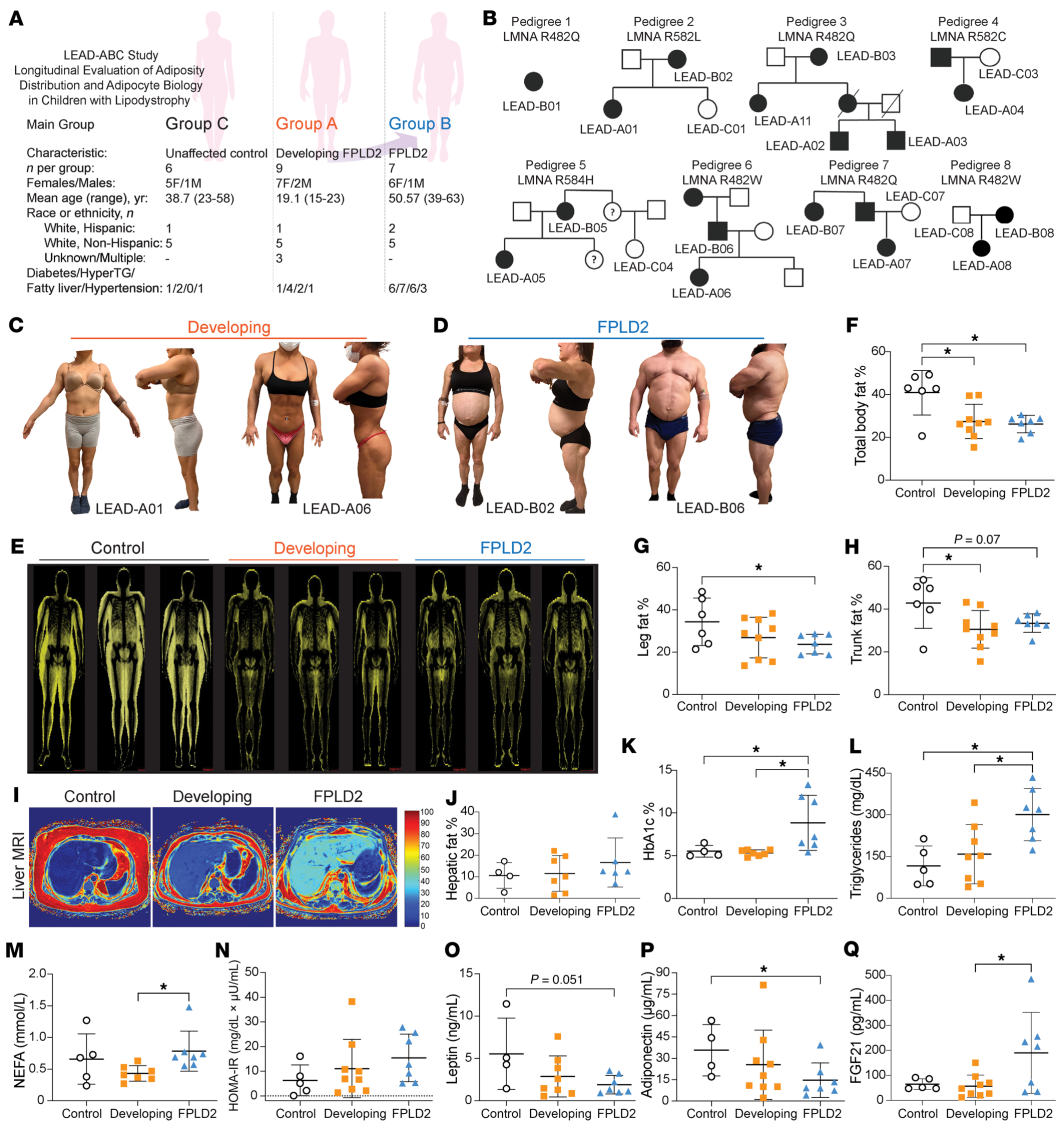
Clinical, metabolic, and molecular characterization of individuals with FPLD2. (**A**) Study design and patient groups. Groups include unaffected family members (control, group C, *n* = 6), genetically affected but clinically unaffected individuals (developing, group A, *n* = 9), and patients with FPLD2 (FPLD2, group B, *n* = 7). (**B**) Pedigrees from multiple families with FPLD2. Filled symbols represent affected individuals, open symbols indicate unaffected individuals, and question marks denote unknown phenotypic status. (**C**) Images of patients with developing FPLD2 with early signs of fat redistribution. (**D**) Images of patients with FPLD2 phenotypes with peripheral lipoatrophy and upper trunk fat accumulation. (**E**) Whole-body fat shadows. Quantification of fat mass percentage in (**F**) total body, (**G**) leg, and (**H**) trunk. (**I**) MRI-based hepatic fat fraction maps with (**J**) quantification. (**K**) Hemoglobin A1c (HbA1c) percentages. (**L**) Triglyceride concentrations in plasma. (**M**) Nonesterified fatty acid (NEFA) concentrations. (**N**) Homeostatic Model Assessment of Insulin Resistance (HOMA-IR) scores. (**O**) Leptin concentrations. (**P**) Adiponectin concentrations. (**Q**) Fibroblast growth factor 21 (FGF21) concentrations. Data are represented as mean ± SD. *P < 0.05. Statistical analyses were performed using 1-way ANOVA, followed by Bonferroni’s post hoc test.

To investigate how pathogenic *LMNA* variants drive FPLD2 progression, we recruited 8 families into the Longitudinal Evaluation of Adiposity Distribution and Adipocyte Biology in Children with Lipodystrophy (LEAD-ABC) study. Participants were stratified into 3 groups: group C, unaffected family members or recruited controls; group A, individuals with a pathogenic variant but without extensive symptoms; and group B, patients with overt FPLD2 (Figure 1A). Whole-genome sequencing identified 5 disease-causing *LMNA* variants across the 8 pedigrees (Figure 1B). Participants with developing FPLD2 (group A) retained visible subcutaneous WAT on limbs and trunk without visceral WAT accumulation (Figure 1C). In contrast, participants with developed FPLD2 (group B) exhibited classical fat redistribution: loss of subcutaneous WAT, increased visceral adiposity, and prominent fat accumulation in the upper neck and face (Figure 1D). These regional changes were visualized using fat shadow imaging (Figure 1E and Supplemental Figure 1A; supplemental material available online with this article; https://doi.org/10.1172/JCI198387DS1). Quantitatively, both developing and developed FPLD2 groups had reduced total body fat percentage compared with controls (Figure 1F). Individuals with FPLD2 specifically had reduced leg fat (Figure 1G), and those with developing FPLD2 already had decreased trunk fat (Figure 1H). MRI revealed no significant hepatic fat accumulation in participants with FPLD2 (Figure 1, I and J).

We next evaluated metabolic parameters across groups. Participants with developed FPLD2 (group B) exhibited increased glycated hemoglobin (Figure 1K), circulating triglycerides (Figure 1L), nonesterified fatty acids (NEFA) (Figure 1M), and glucose area under the curve (AUC) after oral glucose tolerance test (OGTT) (Supplemental Figure 1B), indicating impaired metabolic control. However, Homeostatic Model Assessment of Insulin Resistance (HOMA-IR) (Figure 1N), insulin AUC (Supplemental Figure 1C), NEFA AUC (Supplemental Figure 1D), fasted insulin (Supplemental Figure 1E), and Adipose Tissue Insulin Resistance Index (ADIPO-IR) (Supplemental Figure 1F) did not differ significantly between groups. Circulating leptin (Figure 1O) and adiponectin (Figure 1P) were reduced in FPLD2, consistent with WAT loss. Fibroblast growth factor 21 (FGF21) (Figure 1Q) and growth differentiation factor 15 (GDF15) (Supplemental Figure 1G) were elevated in FPLD2, consistent with stress or metabolic dysfunction (30). Clinical characteristics are summarized in Table 1. Collectively, these data highlight patterns of adipose loss during FPLD2 progression and confirm that participants with developed, but not developing, FPLD2 exhibit metabolic dysfunction (1, 31).

### WAT biopsies from participants with FPLD2 have decreased fatty acid metabolism and increased inflammation gene expression.

**Figure 2:**
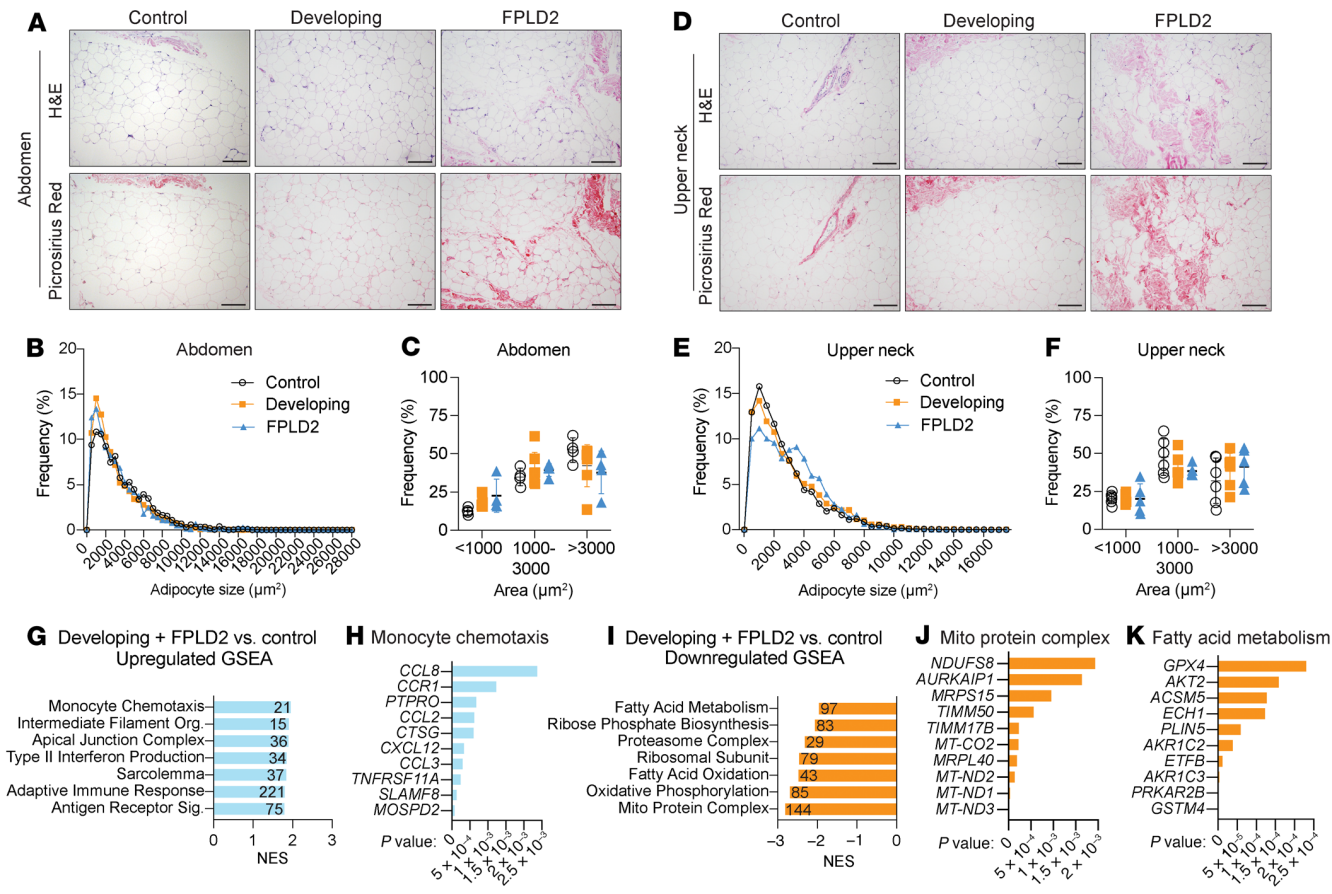
Biopsies from patients with FPLD2 have no change in adipocyte size, but bulk RNA-sequencing suggests decreased metabolism and increased inflammation in patient adipose tissue. (**A**) Representative adipose tissue histological images and Picrosirius red–stained tissue for collagens from biopsies across patient groups in abdominal biopsies. Scale bar: 200 μm. (**B**) Frequency distribution of adipocyte size and (**C**) frequency of adipocytes less or greater than 2,500 μm^2^ from abdominal biopsies. (**D**) Histological images and Picrosirius red analyses on upper neck biopsies. (**E**) Frequency distribution of adipocyte size and (**F**) frequency of adipocytes less or greater than 2,500 μm^2^ from upper neck biopsies. Bulk RNA-sequencing (RNA-Seq) on patient biopsies. Biopsies from group A and B were combined to compare with group C. *n* = 4–5 samples (upper neck and abdomen combined) per group. Gene set enrichment analysis (GSEA) identified (**G**) upregulated pathways with normalized enrichment scores (NES) and (**H**) leading-edge genes for the monocyte chemotaxis pathway. (**I**) Downregulated GSEA pathways and leading-edge genes for (**J**) mito protein complex and (**K**) fatty acid metabolism pathways. Data are represented as mean ± SD. Statistical analyses were performed using 2-way ANOVA, followed by Bonferroni’s post hoc test.

To study mechanisms of adipose loss, we collected subcutaneous WAT biopsies from the abdomen (atrophic) and dorsocervical upper neck (expanding) regions (1). Comparing these depots allows within-individual analysis of adipose redistribution in FPLD2. Histological analyses and Picrosirius red staining showed visibly increased fibrosis in abdominal WAT with disease (Figure 2A), though variable sampling limited quantification. Adipocyte size was unchanged in abdominal samples across disease states (Figure 2, B and C). Similar results were observed in the upper neck (Figure 2, D–F). Skin biopsies were similar across groups (Supplemental Figure 1H). These data indicate that lipid-laden adipocytes remain morphologically comparable across disease states.

We next examined molecular changes driving FPLD2. Combined abdomen and upper neck biopsies within each group were analyzed by bulk RNA sequencing (RNA-Seq) (Figure 2G). Due to limited sample size, developing and developed FPLD2 groups were combined to analyze broad transcriptomic effects of *LMNA* variants. Gene Set Enrichment Analysis (GSEA) revealed upregulated inflammation, intermediate filament, and muscle-related pathways (Figure 2, G and H) and downregulated mitochondrial, translational, and fatty acid metabolism pathways (Figure 2, I–K). Fibrosis- and ECM-related genes were altered, including downregulated *TMND* and upregulated *PDGFA*, *MMP7*, *MMP16*, and multiple collagen genes (Supplemental Figure 1I). Thus, WAT from participants with *LMNA* variants shows decreased metabolism and increased inflammation, suggesting these contribute to disease progression.

### Single nucleus RNA sequencing (snRNA-Seq) identifies depot differences between upper neck and abdominal adipose biopsies.

**Figure 3:**
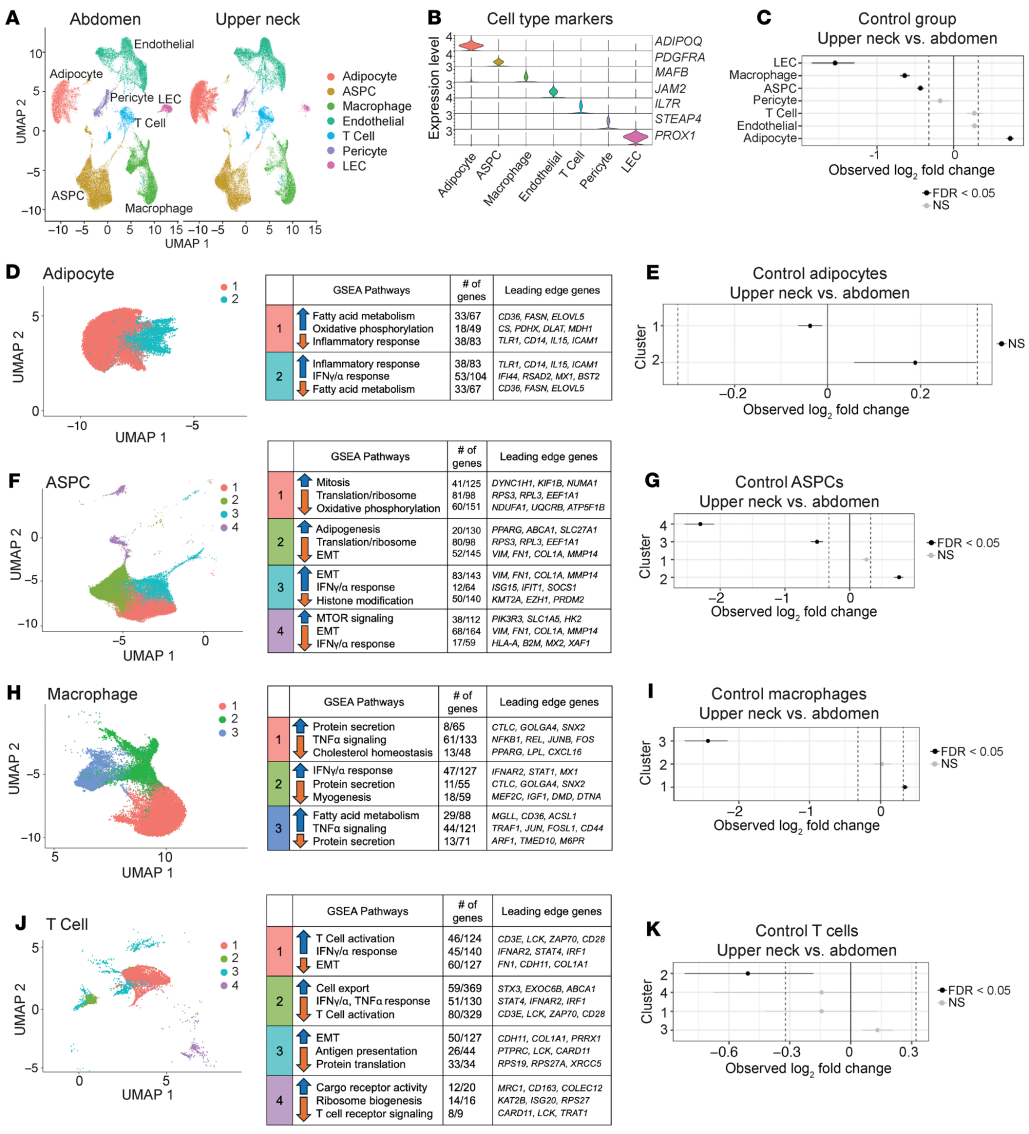
Single nucleus RNA-Seq identifies changes in cell proportions and subclustering analyses between subcutaneous abdomen and upper neck adipose tissue in unaffected patients. (**A**) Uniform manifold approximation and projection (UMAP) of abdomen and upper neck biopsies across all patient groups (A, B, and C). ASPC, adipose stem and progenitor cells; LEC, lymphatic endothelial cells. (**B**) Marker genes for each cell population. (**C**) Permutation tests to identify changes in cell proportions in the upper neck relative to the abdominal biopsies from control patients (group C); changes were statistically significant if the log_2_FC was less or greater than 0.32 and the FDR was less than 0.05. (**D**) Adipocyte subclusters and corresponding GSEA pathways. (**E**) Adipocyte subcluster changes between the upper neck and abdomen in control patients. (**F**) ASPC subclusters with GSEA pathways. EMT, epithelial-mesenchymal transition. (**G**) ASPC subcluster changes in the upper neck relative to abdomen. (**H**) Macrophage subclusters with GSEA pathways. (**I**) Subcluster changes in macrophages in upper neck relative to abdomen. (**J**) T cell subclusters with GSEA pathways. (**K**) Subcluster changes in T cells in upper neck relative to abdomen.

To define depot differences and identify cell types contributing to transcriptomic shifts (Figure 2), we performed snRNA-Seq (10x Genomics) on patient WAT biopsies (*n* = 4–5), identifying 7 major cell populations: adipocytes, adipose stem and progenitor cells (ASPCs), macrophages, endothelial cells, T cells, pericytes, and lymphatic endothelial cells (LECs) (Figure 3, A and B) (32, 33). Other smaller cell populations such as mast cells, natural killer cells, and dendritic cells were detected but grouped into broader categories for downstream analysis.

In control tissues, the upper neck depot contained more adipocytes and fewer LECs, macrophages, and ASPCs than abdominal WAT (Figure 3C). We next sought to characterize the molecular differences in cell states between depots. Adipocytes formed 2 subclusters: cluster 1 (adipogenic) and cluster 2 (pro-inflammatory) (Figure 3D), with no difference in subcluster proportions between depots (Figure 3E). ASPCs divided into 4 subclusters; the upper neck depot had more adipogenic ASPCs and fewer pro-inflammatory or mTOR-high ASPCs, suggesting higher adipogenic potential in the upper neck depot (Figure 3, F and G).

Among immune cells, lipid-associated macrophages (cluster 3) were decreased in the upper neck versus abdomen (Figure 3, H and I), and low-inflammation T cells (cluster 2) were also reduced (Figure 3, J and K). High-inflammation endothelial cells (cluster 3) were increased in the upper neck (Supplemental Figure 2, A and B), whereas high-myogenesis pericytes and high-translation LECs were decreased (Supplemental Figure 2, C–F). Thus, in healthy individuals, upper neck WAT harbors more pro-adipogenic ASPCs and fewer pro-inflammatory macrophages without altering adipocyte identity.

### snRNA-Seq analyses reveal widespread shifts in cellular identity during FPLD2 progression.

**Figure 4:**
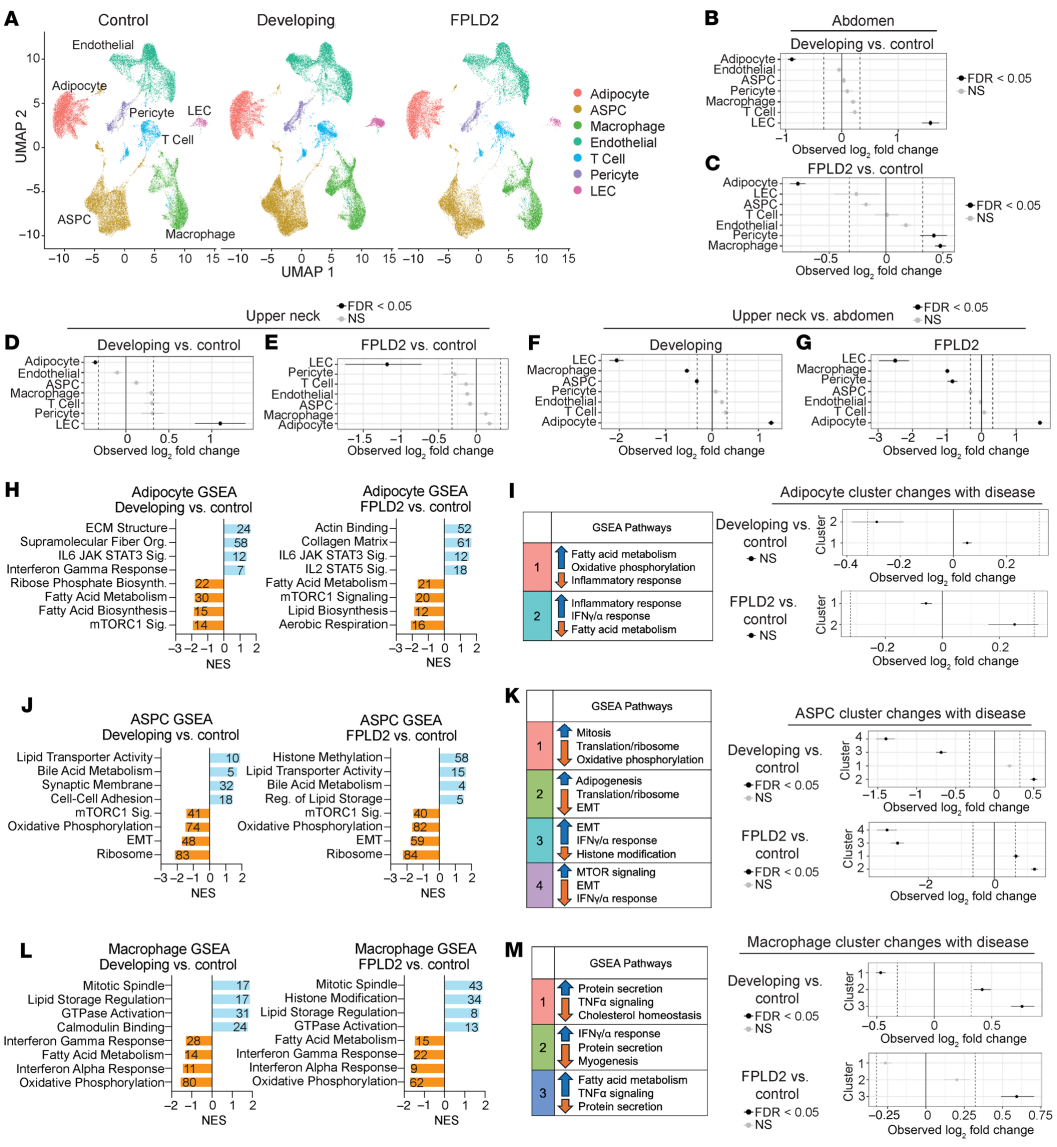
SnRNA-Seq identifies changes in cell proportions and identity with progression of FPLD2. (**A**) UMAP of combined upper neck and abdomen biopsies across disease states; cell type markers are the same as in Figure 3. Permutation tests identified changes in cell proportion from abdominal biopsies from patients with (**B**) developing FPLD2 and (**C**) FPLD2 relative to control and from upper neck biopsies in (**D**) developing FPLD2 and (**E**) FPLD2 relative to control. Cell proportion changes in the upper neck relative to abdomen were identified in (**F**) developing and (**G**) FPLD2 disease states. (**H**) All adipocytes from developing or FPLD2 biopsies were analyzed via GSEA and compared with controls to identify population-level changes in cell identity. Numbers on bar represent the number of genes driving that dataset. Sig, signaling; Org, organization. (**I**) Adipocyte subcluster analyses with corresponding GSEA pathways (same as in Figure 3) and subcluster changes with disease. (**J**) ASPC GSEA in developing or FPLD2 biopsies relative to controls. (**K**) ASPC subcluster changes with disease. (**L**) Macrophage GSEA in developing or FPLD2 biopsies relative to controls. (**M**) Macrophage subcluster changes with disease.

We next examined how cell type composition changes across disease states. We generated UMAP plots combining both depots across our 3 patient groups (Figure 4A), as well as stratified UMAPs by genotype (Supplemental Figure 3A) and sex (Supplemental Figure 3B). UMAPs combining both depots (Figure 4A) showed reduced adipocyte proportions in abdominal WAT of developing and developed FPLD2 (Figure 4, B and C), consistent with WAT atrophy. LECs increased during developing FPLD2 but declined with progression (Figure 4, B and C). Macrophages and pericytes increased in FPLD2 abdominal WAT (Figure 4C). In the upper neck, adipocytes decreased in developing FPLD2 but stabilized thereafter (Figure 4, D and E). LECs followed a similar transient pattern (Figure 4, D and E). Depot differences seen in controls (Figure 3C) persisted across genotypes (Figure 4, F and G), suggesting intrinsic regional identity.

After characterizing changes in cell type proportions, we next studied how cell identities change with FPLD2. GSEA of adipocytes showed reduced lipid metabolism and increased ECM and inflammation (Figure 4H), with leading-edge genes listed in Supplemental Table 1. These data align closely with results from bulk RNA-Seq of WAT from participants with FPLD2, which also showed suppression of fatty acid metabolism and increased inflammation (Figure 2, G–K). Adipocyte subcluster composition was unchanged (Figure 4I). ASPCs displayed decreased oxidative phosphorylation and ribosomal genes but increased lipid metabolism (Figure 4J). Pro-adipogenic ASPCs (cluster 2) were expanded, while pro-inflammatory ASPCs (cluster 3) were reduced (Figure 4K). Macrophages overall were less inflammatory (Figure 4L), though lipid-associated macrophages (cluster 3) increased (Figure 4M). Endothelial cell, T cell, pericyte, and LEC populations showed altered metabolic, translational, and inflammatory gene expression (Supplemental Figure 3, C–J). Collectively, adipocytes exhibit impaired fatty acid metabolism, ASPCs adopt a more adipogenic state, and macrophages show lipid-scavenging features during FPLD2 progression.

### Cell proportions change with FPLD2 progression in a depot-specific manner.

We next assessed how these cell proportion changes differed by depot. In FPLD2, pro-inflammatory adipocytes were enriched in the upper neck relative to the abdomen (Supplemental Figure 4A). Pro-adipogenic ASPCs increased in developing FPLD2 (Supplemental Figure 4B), possibly explaining dorsocervical WAT expansion. Lipid-associated macrophages were reduced in the upper neck (Supplemental Figure 4C), pro-inflammatory endothelial cells were increased in the upper neck (Supplemental Figure 4D), and pro-inflammatory T cells rose in developing FPLD2 (Supplemental Figure 4E). Pericytes were unchanged (Supplemental Figure 4F), and pro-adipogenic LECs were increased in early disease (Supplemental Figure 4G). Thus, depot-specific cellular shifts, particularly increased pro-inflammatory adipocytes and endothelial cells in the upper neck, may underlie the contrasting fat redistribution and metabolic features of FPLD2.

### Inducible lamin A/C knockout in adipocytes causes lipodystrophy but not metabolic dysfunction.

**Figure 5:**
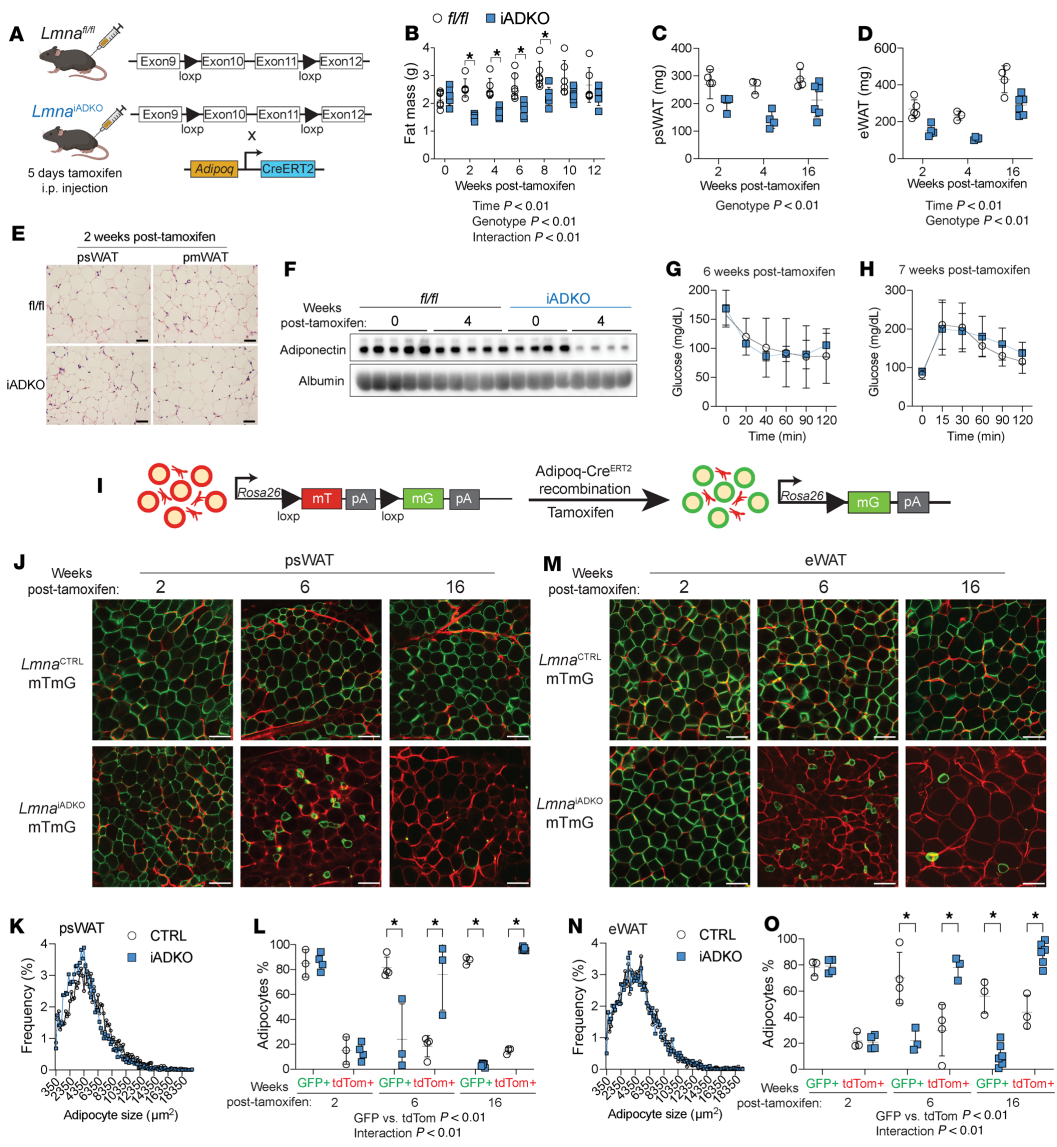
Tamoxifen-inducible adipocyte-specific *Lmna* knockout causes transient adipose tissue loss, and *Lmna*-deficient adipocytes shrink and disappear. All data from male mice besides histology from female mice. (**A**) Gene schematic of *Lmna^fl/fl^* control mice and *Lmna*^iADKO^ mice. Adult mice were administered tamoxifen intraperitoneally for 5 consecutive days to induce recombination. (**B**) Fat mass after tamoxifen administration (*n* = 6). (**C**) Posterior subcutaneous WAT (psWAT) weights and (**D**) epididymal WAT (eWAT) weights at 2, 4, and 16 weeks posttamoxifen (*n* = 3–6). (**E**) Representative histology of psWAT and parametrial WAT (pmWAT) 2 weeks posttamoxifen. Scale bar: 40 μm. (**F**) Serum adiponectin immunoblot at 0 and 4 weeks posttamoxifen (*n* = 4–5). (**G**) Insulin tolerance test 6 weeks posttamoxifen (*n* = 6). (**H**) Glucose tolerance test 7 weeks posttamoxifen (*n* = 6). (**I**) Schematic of mTmG reporter system induced by tamoxifen-mediated Cre activity. (**J**) Representative fresh confocal micrographs of psWAT (scale bar: 100 μm) and (**K**) quantification of psWAT adipocyte size at 2 weeks posttamoxifen (*n* = 3–4). (**L**) Quantification of psWAT GFP^+^ or tdTomato^+^ adipocytes. (**M**) Confocal micrographs of eWAT and (**N**) quantification of eWAT adipocyte size at 2 weeks posttamoxifen. (**O**) Quantification of eWAT GFP^+^ or tdTomato^+^ adipocytes. Data are represented as mean ± SD. *P < 0.05. Statistical analyses were performed using 2-way ANOVA, followed by Bonferroni’s post hoc test.

Bulk RNA-Seq and snRNA-Seq data from participants with FPLD2 revealed downregulation of metabolic pathways and upregulation of inflammatory signaling (Figures 2 and 4). To further investigate roles of lamin A/C in adipocyte maintenance, we generated *Lmna*^iADKO^ mice, extending our prior constitutive adiponectin-Cre (*Adipoq*-Cre) model (28). Tamoxifen was administered intraperitoneally for 5 days to *Lmna^fl/fl^* and *Lmna*^iADKO^ mice (Figure 5A). Two weeks posttamoxifen, *Lmna*^iADKO^ mice exhibited reduced fat mass (Figure 5B) without changes in body weight or lean mass (Supplemental Figure 5, A and B). Posterior subcutaneous (psWAT) and epididymal WAT (eWAT) weights decreased at 2 to 4 weeks posttamoxifen, partially recovering by 16 weeks (Figure 5, C and D). Retroperitoneal WAT decreased at 2 weeks posttamoxifen; brown adipose tissue (BAT), perirenal WAT, and liver were unchanged (Supplemental Figure 5C). No sex differences were observed (Supplemental Figure 5D), and both sexes were used throughout these mouse studies. Histology revealed no overt WAT or liver changes (Figure 5E and Supplemental Figure 5F), though slight BAT whitening and partial bone marrow adipose loss appeared by 8 weeks posttamoxifen. Uncoupling protein 1 expression was unchanged in *Lmna*^iADKO^ mouse BAT 2 weeks posttamoxifen and undetectable in psWAT (Supplemental Figure 5, F and G). Circulating adiponectin decreased at 4 weeks posttamoxifen (Figure 5F and Supplemental Figure 5H), but insulin sensitivity and glucose tolerance remained normal at 6 and 12 weeks posttamoxifen (Figure 5, G and H, and Supplemental Figure 5, I and J). *Lmna*^iADKO^ mice thus model early adipocyte loss without confounding metabolic dysfunction, ideal for mechanistic studies.

### Lamin A/C-deficient adipocytes shrink, become misshapen, and disappear from WAT.

Using the mTmG reporter system (34), we tracked *Lmna*-KO adipocytes via GFP expression (Figure 5I). Two weeks posttamoxifen, GFP^+^ adipocytes in psWAT were widespread and morphologically normal despite reduced fat mass, suggesting fewer adipocytes (Figure 5, J–L). By 6 weeks posttamoxifen, *Lmna*-KO GFP^+^ adipocytes showed shrinkage, irregular shape, and membrane budding, while tdTomato^+^ cells increased, suggesting compensatory adipogenesis (Figure 5, J and L). By 16 weeks posttamoxifen, GFP^+^ adipocytes were nearly absent in psWAT from *Lmna*^iADKO^ mice. This same pattern was observed in eWAT (Figure 5, M–O). Small GFP^+^ cells in WAT at 6 weeks posttamoxifen disappeared by 16 weeks, suggesting that KO adipocytes do not persist in *Lmna*^iADKO^ WAT (Supplemental Figure 6, A and B). Flow cytometry confirmed GFP^+^ stromal vascular cells (SVCs) were not elevated at 6 weeks posttamoxifen in *Lmna*^iADKO^ WAT, indicating no evidence of dedifferentiation of KO adipocytes (Supplemental Figure 6, D and E). These data indicate *Lmna*-KO adipocytes progressively atrophy and are cleared from tissue, supporting lamin A/C’s essential role in adipocyte maintenance.

### Lmna^iADKO^ WAT mirrors FPLD2 WAT: increased inflammation, decreased fatty acid metabolism.

**Figure 6:**
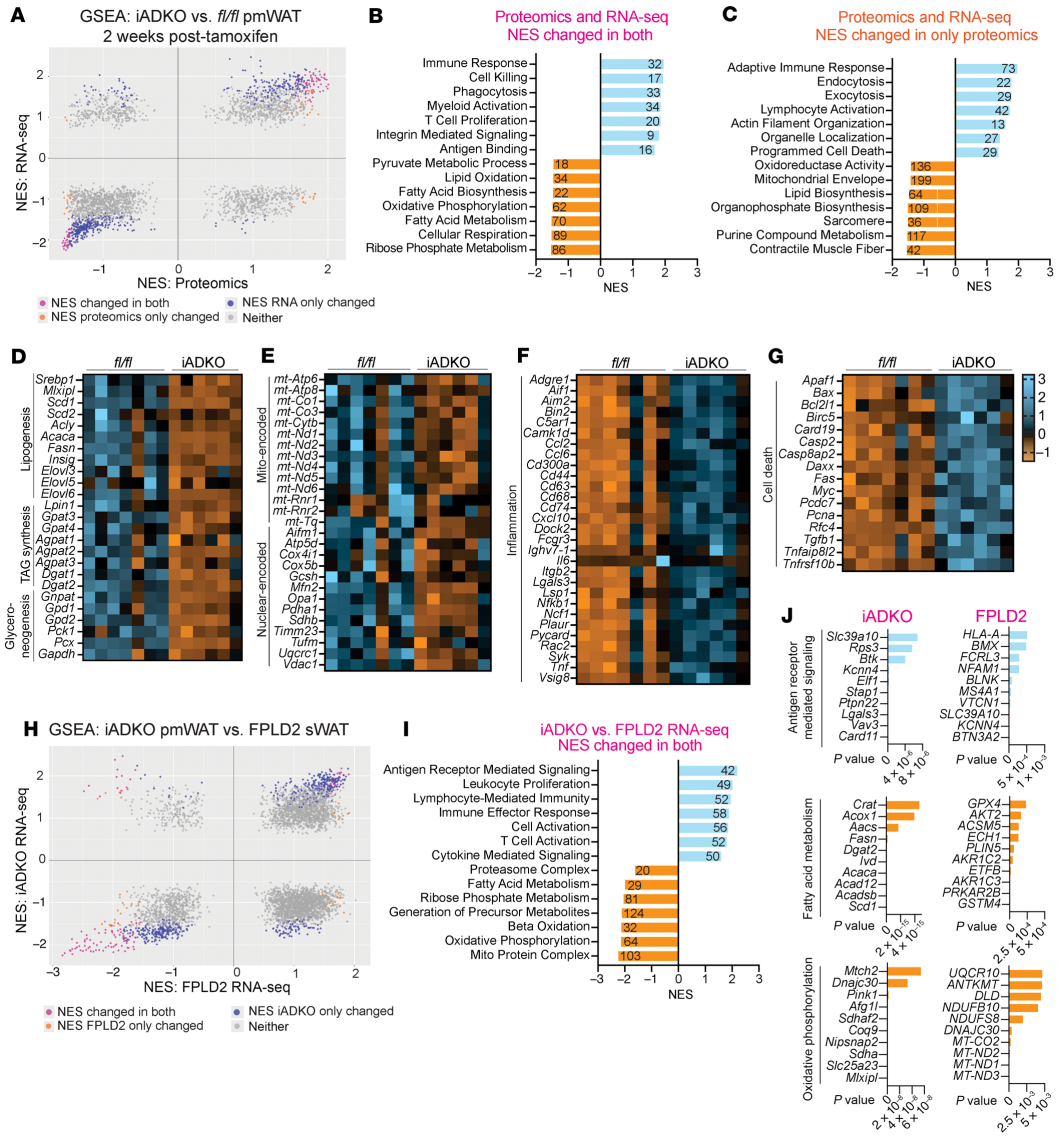
Bulk RNA-Seq and proteomics of *Lmna*^iADKO^ WAT reveals increased inflammation and reduced fatty acid metabolism and mitochondrial pathways, similar to human FPLD2 biopsies. All data from female mice. *Lmna*^iADKO^ and *Lmna^fl/fl^* pmWAT 2 weeks posttamoxifen was used for RNA-Seq (*n* = 6–7) and proteomics (*n* = 5). (**A**) Integrative GSEA on bulk RNA-Seq and proteomics. Pink dots symbolize that NES significantly changed in both RNA-Seq and proteomics datasets, orange dots that NES changed only in proteomics, and blue that NES changed only in RNA-Seq. (**B**) Highlighted GSEA pathways changed in both proteomics and RNA-Seq. Numbers on bars represent number of overlapping genes driving pathways. (**C**) Highlighted GSEA pathways changed in proteomics only. Heatmaps showing selected changes in genes driving (**D**) lipid biosynthesis, (**E**) mitochondrial function, (**F**) inflammation, and (**G**) cell death. TAG, triacylglycerol. (**H**) Integrative GSEA of *Lmna*^iADKO^ pmWAT bulk RNA-Seq compared with FPLD2 bulk RNA-Seq (Figure 2). (**I**) Highlighted GSEA pathways changed in both mouse and human RNA-Seq datasets. (**J**) Leading-edge genes for GSEA pathways related to inflammation, fatty acid metabolism, and mitochondrial function for either iADKO or FPLD2 samples.

We performed bulk RNA-Seq and proteomics on pmWAT 2 weeks posttamoxifen, prior to morphological changes, and integrated GSEAs between both datasets (Figure 6A). GSEA revealed upregulated immune processes (myeloid activation, antigen binding) and downregulated oxidative phosphorylation and fatty acid biosynthesis (Figure 6B). Proteomics specifically showed elevated cell death and suppressed muscle-associated pathways (Figure 6C). Lipogenic genes and mitochondrial genes were repressed (Figure 6, D and E), while inflammation and cell death genes increased (Figure 6, F and G). Comparison with constitutive *Lmna*^ADKO^ WAT showed concordant suppression of metabolism and increased inflammation (Supplemental Figure 7, A–D). Integration with human FPLD2 RNA-Seq verified overlapping gene expression patterns: increased inflammation and decreased mitochondrial/lipid metabolism pathways (Figure 6, H–J), highlighting lamin A/C’s role in adipocyte homeostasis.

### Lipogenic and mitochondrial protein expression is lower in Lmna^iADKO^ WAT, accompanied by decreased respiration and altered mitochondrial structure.

**Figure 7:**
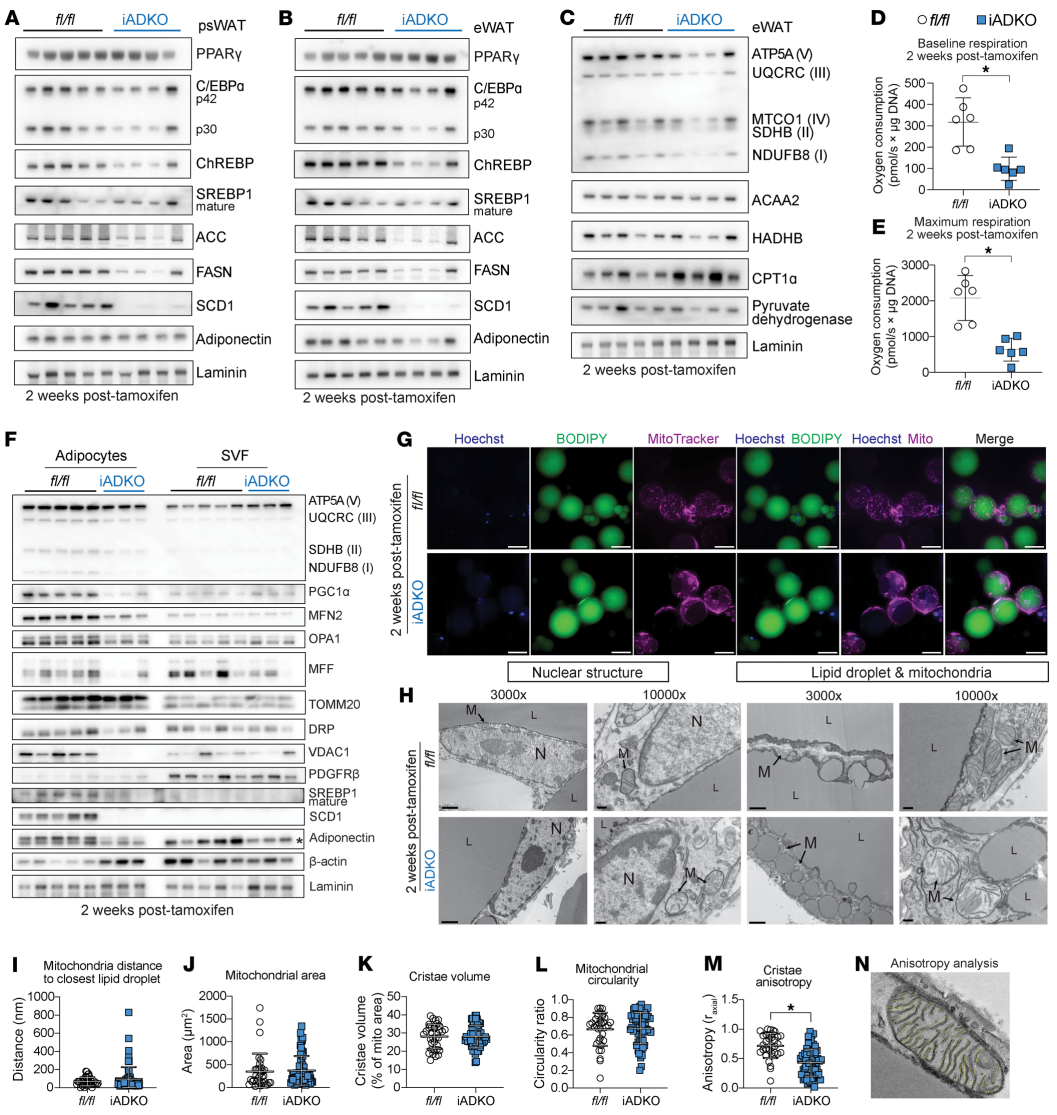
*Lmna*^iADKO^ adipocytes have reduced oxygen consumption and dysfunctional mitochondrial dynamics and structure. Data in **A** to **C** and in **H** are from male mice, and all other data are from female mice. Immunoblot analyses 2 weeks posttamoxifen of lipid metabolism and lipogenesis proteins in (**A**) psWAT and (**B**) eWAT (*n* = 4–5). Loading control = laminin. (**C**) Immunoblot of mitochondrial proteins in eWAT 2 weeks posttamoxifen (*n* = 4–5). Loading control = laminin. Oroboros Oxygraph-2k analyses of (**D**) baseline and (**E**) maximum respiration in floated adipocytes from pmWAT 2 weeks posttamoxifen (*n* = 6). (**F**) Immunoblot of mitochondrial function and dynamics proteins in floated adipocytes and stromal vascular fraction (SVF) from pmWAT 2 weeks posttamoxifen (*n* = 3–5). * indicates nonspecific band. Loading control = laminin and β-actin. (**G**) Confocal micrographs of floated adipocytes from pmWAT stained for nuclei (Hoechst), lipid (BODIPY), and mitochondria (MitoTracker Red); scale bar: 50 μm. (**H**) Transmission electron micrographs of eWAT 2 weeks posttamoxifen. Scale bar: 1 μm for original magnification, 3,000×, images; 200 nm for original magnification, 10,000×, images. N, nucleus; L, lipid droplet; M, mitochondria. Quantification of transmission electron micrographs: (**I**) distance of mitochondria to closest lipid droplet, (**J**) mitochondrial area, (**K**) cristae volume per mitochondria, (**L**) mitochondrial circularity, and (**M**) cristae anisotropy with (**N**) example of anisotropy analysis. An average of 9 adipocyte mitochondria were quantified per mouse; *n* = 5–6. Data are represented as mean ± SD. *P < 0.05. Statistical analyses were performed using Student’s *t* test.

In *Lmna*^iADKO^ psWAT, which showed no change in mass 2 weeks posttamoxifen (Figure 5C), PPARγ and C/EBPα were unchanged in protein expression, whereas ChREBP and key lipogenic enzymes (ACC, FASN, SCD1) were reduced (Figure 7A and Supplemental Figure 8A). eWAT showed similar reductions, with slightly increased PPARγ, possibly compensatory (Figure 7B and Supplemental Figure 8B). Given the well-established link between lipid metabolism and mitochondrial function (35, 36), we examined mitochondrial protein expression and saw that oxidative phosphorylation proteins were reduced in *Lmna*^iADKO^ eWAT (Figure 7C and Supplemental Figure 8C), with decreased baseline, maximal, and ATP-linked respiration; spare respiratory capacity; and proton leak in pmWAT adipocytes (Figure 7, D and E, and Supplemental Figure 8, D–F). In contrast, *Lmna*-KO adipocytes isolated from psWAT at the same time point did not exhibit changes in mitochondrial respiration (Supplemental Figure 8, G–K), suggesting that reductions in lipid metabolism proteins (Figure 7A) may precede overt mitochondrial dysfunction following *Lmna* deletion in adipocytes. Mitochondrial DNA content remained unchanged in psWAT and pmWAT (Supplemental Figure 8, L and M). Mitochondrial biogenesis and fission-fusion regulators (PGC1α, MFN2, OPA1, VDAC1) were decreased in expression in *Lmna*^iADKO^ adipocytes; TOMM20 was unchanged (Figure 7F and Supplemental Figure 8, N and O). Imaging revealed irregular mitochondrial clustering and polarization in *Lmna*-KO adipocytes compared with controls (37), a potential sign of cell damage, as previously observed in MFN2-KO cells (38) (Figure 7G). Transmission electron microscopy (TEM) of *Lmna*^iADKO^ WAT 2 weeks posttamoxifen showed small lipid droplets with surrounding mitochondria, suggesting active lipid synthesis, droplet fission, or budding (Figure 7H). Cristae were disorganized and adipocyte had potentially altered heterochromatin distribution, though overall mitochondrial area and droplet contacts were unchanged (Figure 7, H–N). Deletion of *Lmna* in adipocytes thus impairs mitochondrial function and structure, contributing to adipocyte loss.

### Adipocyte loss is not driven by increased lipolysis.

Following our TEM observations of increased small lipid droplets in *Lmna*^iADKO^ adipocytes, we investigated whether enhanced lipolysis might cause adipocyte loss in vivo. Circulating glycerol decreased at 4 weeks posttamoxifen and later, under fed and fasted conditions in *Lmna*^iADKO^ mice (Supplemental Figure 9A). Isoproterenol-stimulated lipolysis was unchanged when normalized to fat mass at 4 weeks posttamoxifen in *Lmna*^iADKO^ mice (Supplemental Figure 9, B and C). Bulk RNA-Seq showed downregulation of lipolytic genes at 2 weeks posttamoxifen (Supplemental Figure 9D), but lipolytic proteins were not suppressed (Supplemental Figure 9, E and F), indicating adipocyte loss occurs via lipolysis-independent mechanisms.

### Lmna-deficient adipocytes show cell-autonomous pro-inflammatory gene expression.

snRNA-Seq in human FPLD2 suggested immune signatures arise partly from macrophages and T cells. Spectral flow cytometry of *Lmna*^iADKO^ psWAT and pmWAT 2 weeks posttamoxifen showed no differences in total SVCs, CD45^+^ cells, adipose tissue macrophages (ATMs; CD64^+^), or identity of macrophages (CD11c^+^, TIM4^+^, CD163^+^) or T cells (CD4^+^, CD8^+^) (Supplemental Figure 10, A–N). Whole WAT from *Lmna*^iADKO^ mice had few inflammatory transcript changes (Supplemental Figure 10, O and P), but isolated *Lmna*-KO adipocytes 2 weeks posttamoxifen showed elevated *Il6*, *Il10*, *Nlrp3*, *Il1b*, and *Tnfa*, whereas SVF had minor changes (Supplemental Figure 10, Q–T), suggesting cell-autonomous inflammatory signaling upon loss of lamin A/C. Cleaved caspase-3 was undetectable at 2, 4, and 6 weeks posttamoxifen in *Lmna*^iADKO^ WAT, and cGAS-STING markers were unchanged (Supplemental Figure 10, U–W), indicating a nonclassical, asynchronous cell death mechanism.

### Fundamental role of lamin A/C in regulating lipid metabolism and inflammatory gene expression across cell types.

**Figure 8:**
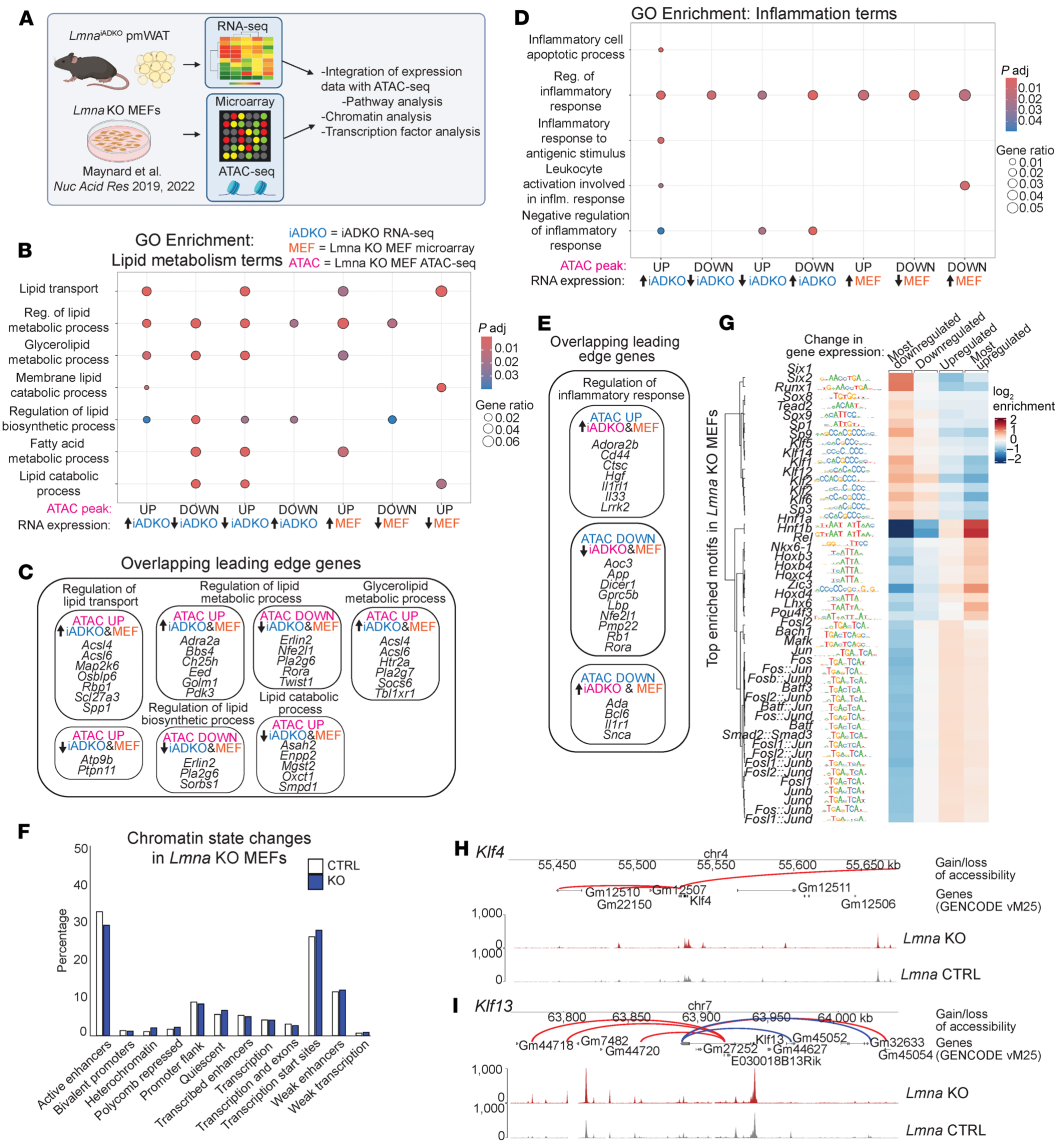
*Lmna*-KO MEFs and adipocytes have shared patterns of altered gene expression, correlated with changes in chromatin accessibility. (**A**) Schematic of dataset integration between *Lmna*^iADKO^ pmWAT RNA-Seq and *Lmna-*KO MEF microarray (39, 40) and assay for transposase-accessible chromatin sequencing (ATAC-Seq). Overlapping changed genes in RNA-Seq of *Lmna*^iADKO^ WAT (iADKO), *Lmna-*KO MEF microarray (MEF), and *Lmna-*KO MEF ATAC-Seq (ATAC) were identified, and GSEA was run to identify changes in (**B**) lipid metabolism Gene Ontology (GO) terms with their corresponding (**C**) overlapping leading-edge genes, along with (**D**) inflammatory GO terms with (**E**) corresponding overlapping leading-edge genes. (**F**) Distribution of chromatin features represented by percentage of overall chromatin composition compared between *Lmna* control (CTRL) and KO MEF ATAC-Seq. (**G**) Enriched motifs in *Lmna-*KO MEFs, stratified into 4 bins based on microarray gene expression changes, from most downregulated to most upregulated. (**H** and **I**) ATAC-Seq signal tracks and visualization of significantly enriched regions and their predicted target genes. Red lines represent enhancers with a positive fold-change upon *Lmna* KO, and blue lines represent negative.

We observed consistent gene expression changes in *Lmna*-deficient mouse adipocytes and human FPLD2 WAT: suppressed lipid metabolism and increased inflammation. To assess whether these changes are conserved across cell types, we analyzed publicly available microarray and assay for transposase-accessible chromatin sequencing (ATAC-Seq) data from *Lmna*-KO MEFs, which also showed impaired mitochondrial respiration and irregular mitochondrial localization (Figure 8A) (39, 40). Integration of *Lmna*^iADKO^ WAT RNA-Seq with *Lmna*-KO MEF datasets revealed coordinated dysregulation of lipid metabolism and inflammation at transcript and chromatin levels (Figure 8, A–E). Overlapping genes included *Acsl4*, *Acsl6*, *Rbp1*, and *Scl27a3* (lipid homeostasis) and *Il1rl1*, *Il33*, and *Cd44* (inflammation), suggesting lamin A/C directly regulates these programs across cell types.

ATAC-Seq peak distribution across chromatin features was unchanged between control and KO MEFs (Figure 8F), consistent with lamin A/C binding ~30%–40% of the genome (41, 42). Motif enrichment analysis near differentially expressed genes revealed KLF family motifs enriched in downregulated genes and HNF1A/B, HOX, and FOS/JUN motifs near upregulated genes (Figure 8G). *Klf4* and *Klf13* loci showed altered enhancer accessibility and reduced expression (Figure 8, H and I). KLF4 regulates adipocyte differentiation (44), and KLF13 has been implicated in suppressing inflammation (45), suggesting loss of lamin A/C impairs adipocyte function and enhances inflammatory signaling. Together, these data indicate that lamin A/C loss disrupts enhancer accessibility and transcriptional regulation of lipid- and inflammation-related genes across diverse cell types, highlighting a conserved role in maintaining metabolic and immune gene programs, beyond adipocytes or FPLD2.

## Discussion

In this study, we investigated mechanisms underlying lipodystrophy using human, mouse, and cell culture models to better understand FPLD2 progression and pathogenesis. We first analyzed participants with developing and developed FPLD2 and found, as expected, reduced body fat compared with healthy controls (7). Even before overt adipose loss and metabolic dysfunction, participants with developing FPLD2 displayed reduced total body fat. Only patients with developed FPLD2 had elevated HbA1c, hyperlipidemia, and FGF21, indicating that systemic metabolic dysfunction emerges later with severe adipose loss. The severity of metabolic syndrome in our FPLD2 groups may be underestimated, as many participants used metformin or lipid-lowering agents; however, some controls also showed metabolic abnormalities, making them appropriate comparators for depot-specific analyses.

Histological analyses of WAT from participants with FPLD2 revealed no significant change in adipocyte size between depots or groups, though fibrotic deposition appeared increased but heterogeneous. To address this variability and enable earlier detection, plasma biomarkers such as endotrophin, PIINP, or TIMP-1/2 should be explored (46–48). Although plasma inflammatory markers were unchanged (Table 1), identifying circulating disease markers could aid early diagnosis.

Mouse studies supported our human data: At 2 weeks posttamoxifen, analogous to developing FPLD2, adipocyte size was unchanged in *Lmna*^iADKO^ WAT (Figure 5, K and N). Weeks later, adipocytes became shrunken and misshapen, followed by compensatory adipogenesis (Figure 5, L and O). While *Lmna*^iADKO^ WAT showed partial adipose mass recovery (Figure 5, C and D), regeneration was incomplete, suggesting limited precursor capacity. Mice may regenerate adipocytes more effectively than humans, potentially explaining why mouse models incompletely recapitulate FPLD2. We propose that *LMNA*-variant precursors form adipocytes early in life, but as mature adipocytes turn over (~10-year lifespan), new adipocytes fail to efficiently replace them (49), consistent with FPLD2 onset around puberty.

Cross-species transcriptomic comparisons of human WAT and *Lmna*^iADKO^ pmWAT revealed consistent suppression of lipid metabolism and adipogenesis genes, particularly those in lipogenesis, TAG synthesis, and glyceroneogenesis. SCD1 was nearly absent in *Lmna*^iADKO^ WAT (Figure 7, A and B), and snRNA-Seq showed reduced *SCD1* in developing and FPLD2 adipocytes (log_2_FC = –0.855 and –0.497, respectively). Lipid metabolic gene suppression extended to macrophages, pericytes, and endothelial cells in human FPLD2. Key lipogenic regulator ChREBP was decreased in *Lmna*^iADKO^ WAT 2 weeks posttamoxifen (Figure 7, A and B). Prior work showed that adipocyte-specific ChREBP KO leads to reduced pmWAT but not subcutaneous WAT in 16-week-old mice (50), potentially explaining the susceptibility of gonadal WAT to be lost before psWAT in *Lmna*^iADKO^ mice (Figure 5, C and D, and Supplemental Figure 5D). Although SREBP1 protein levels were unchanged in total *Lmna*^iADKO^ WAT, it was reduced in isolated adipocytes (Figure 7F), with downstream target repression (Figure 6D and Figure 7, A and B), suggesting impaired transcriptional activity. SREBP1 has an increasingly appreciated contribution to lipogenesis in WAT (51) and has been shown to interact with lamin A, with FPLD2-causing *LMNA* variants disrupting the interaction between lamin A and SREBP1 (52). Mice with constitutively active SREBP1 in adipose tissue exhibited lipodystrophy and metabolic dysfunction, driven by reduced adipogenic gene programs, further implicating SREBP1 with impaired WAT function (53). Moreover, *Scd1* KO induces autophagic adipocyte death, underscoring the importance of intact lipogenesis (54). The mechanism by which lamin A/C regulates lipid metabolism and adipocyte survival remains to be defined.

Across datasets, mitochondrial gene expression and function were impaired. *LMNA*-variant WAT showed reduced oxidative phosphorylation across cell types, consistent with prior findings in *Lmna*-KO MEFs, hearts from *Lmna*-KO mice, and *LMNA* R482W induced pluripotent stem cells (39, 55, 56). While the sequence of lipid and mitochondrial defects is uncertain, lipogenic enzyme downregulation preceded oxidative phosphorylation disruption in *Lmna*^iADKO^ adipocytes (Figure 7A and Supplemental Figure 8, G–K). Inflammatory pathways were also consistently upregulated across datasets. However, flow cytometry on *Lmna*^iADKO^ WAT and snRNA-Seq of human WAT revealed no increase in activated macrophages or pro-inflammatory T cells. Instead, RT-qPCR showed upregulation of immune genes within *Lmna*-KO adipocytes, suggesting adipocyte-intrinsic inflammation, corroborated by IL6/JAK/STAT3 and IL2/STAT5 activation in patient adipocytes (Figure 4H and Supplemental Figure 10, Q and S). Inflammation may arise directly from lamin A/C loss or secondarily from lipogenic or mitochondrial defects. Indeed, ChREBP-KO adipocytes recruit ATMs (50), increased inflammation in human WAT biopsies is associated with decreased lipogenic markers (57), and mitochondrial *Crif1* haploinsufficiency induces cytokine release and macrophage infiltration (58).

snRNA-Seq provided insight into cellular contributions to disease progression. LECs increased in developing but declined in established FPLD2 (Figure 4, B–E), suggesting lymphatic remodeling in response to adipocyte loss. Lymphatics regulate lipid and immune transport in WAT (59–61), and neurotensin-mediated LEC/adipocyte signaling represses BAT thermogenesis (62). Elevated VEGF-D, which expands lymphatics, improves metabolic health (63). Thus, changing LEC abundance may represent a compensatory but ultimately insufficient response. ASPCs increased in both developing and developed FPLD2, suggesting attempted regeneration hindered by *LMNA* variant–driven adipogenic defects (22, 26, 64, 65). Lipid-associated macrophages also increased (Figure 4M), possibly to clear lipids from dying adipocytes, consistent with prior lipodystrophy models (66).

Our study offers a direct comparison of atrophic (abdominal) and hypertrophic (upper neck) depots in FPLD2. Adipocytes were transcriptionally similar, but stromal cells differed: Upper neck ASPCs were more pro-adipogenic, abdominal macrophages more pro-inflammatory, pericytes more adipogenic in the abdomen, and endothelial cells more inflammatory in the upper neck. These shifts may underlie depot-selective remodeling. Although prior studies reported increased fibrosis and smaller adipocytes in neck WAT (67), we saw neither. However, snRNA-Seq revealed distinct pro-inflammatory and adipogenic cell type shifts with disease progression (Supplemental Figure 4, A–D), elucidating depot-specific WAT remodeling.

Finally, we investigated the chromatin basis of transcriptional dysregulation. Lamin A/C loss alters chromatin accessibility both within and outside lamina-associated domains (39, 68). We found shared gene expression changes between *Lmna*^iADKO^ WAT and *Lmna*-KO MEFs (Figure 8), notably suppression of lipid metabolism and activation of inflammatory pathways, potentially driven by altered enhancer accessibility. Limited overlap likely reflects cell type differences and lamin A/C’s context-dependent genomic interactions (69). Some changes may stem from stress-induced chromatin remodeling (70). Though our focus was WAT and MEFs, effects in other metabolic tissues warrant study. Collectively, our findings support a model in which lamin A/C disruption broadly impairs chromatin organization, driving lipid, mitochondrial, and inflammatory dysregulation across cell types, thereby maintaining metabolic homeostasis under normal conditions.

Despite these insights, our study has several limitations. Our human cohort spans a wide age range and includes both sexes, which may introduce variability and confound interpretation. We did not obtain biopsies from leg depots, which also undergo early atrophy in *LMNA*-related lipodystrophy. Because of adipocyte fragility, we used snRNA-Seq instead of single-cell RNA-Seq, limiting detection of cytoplasmic transcripts. In mice, our adipocyte-specific *Lmna*-KO model does not genetically mirror human FPLD2; future studies should employ knockin models to assess pathogenic *LMNA* variants in WAT. Although both sexes were used, the mechanistic role of sex in lipodystrophy remains undefined. Another caveat is that tamoxifen at 100 mg/kg for 5 days can itself induce lipoatrophy (71). To minimize off-target effects, we used 50 mg/kg for 5 days, with control *Lmna^fl/fl^* mice receiving identical treatment. In summary, lamin A/C is essential for adipocyte homeostasis, survival, lipid metabolism, and mitochondrial function; its disruption drives inflammation, adipocyte shrinkage, and eventual cell loss. These findings reveal key pathways linking lamin A/C dysfunction to lipodystrophy and provide a framework for future therapies.

## Methods

### Sex as a biological variable.

For human studies, we were limited by the rarity of FPLD2 and were unable to control for sex during patient recruitment; future studies should include larger numbers of patients to allow these comparisons. Women tend to have a more severe FPLD2 phenotype, so most of the participants in our studies were women. For mouse studies, we analyzed both sexes during initial phenotyping and found that both male and female *Lmna*^iADKO^ mice had a similar pattern of fat mass loss at 2 weeks posttamoxifen, with comparable necropsy data at this time point. Therefore, we used both sexes of mice throughout this study, designated in the figure legend or by the use of eWAT for male and pmWAT for female mice.

### Human participants.

Participants carrying pathogenic LMNA variants (R482Q, *n* = 6; R482W, *n* = 4; R582L, *n* = 2; R584H, *n* = 2; R582C, *n* = 1) and their unaffected relatives were enrolled in this prospective, longitudinal observational study. Participants were classified into 3 groups: group A, young individuals (ages 15–23) with LMNA variants who were developing signs of lipodystrophy but retained residual adipose depots; group B, affected adult relatives (ages 39–63) with overt partial lipodystrophy characterized by fat loss from extremities, gluteal regions, and abdominal wall; and group C, unaffected relatives (ages 23–58) serving as negative controls.

Physical examinations included anthropometric measurements (height, weight, waist and hip circumference), blood pressure, pulse rate, and full physical exam. Fasting blood samples (≥10 hours) were collected to measure plasma glucose, insulin, HbA1c, C-peptide, leptin, adiponectin, triglycerides, HDL/LDL-cholesterol, liver enzymes (ALT, AST, GGT), kidney function (blood urea nitrogen, creatinine), C-reactive protein, FGF21, and GDF15.

A 5-hour OGTT with 75 g glucose was performed with blood sampling every 30 minutes to assess glucose, insulin, NEFA, and C-peptide. Indices of insulin resistance, including HOMA-IR and ADIPO-IR, were calculated from fasting values. Body composition was assessed using 2 complementary methods: MRI-based fat quantification (3 T, Philips Healthcare) analyzed with a custom MATLAB program and whole-body dual-energy X-ray absorptiometry (GE Lunar Prodigy, model PA +41,744). Hepatic fat was quantified on a 3 T clinical MRI system using a torso phased-array surface coil and breath-hold, single-shot, turbo-spin-echo localization sequences. Fat content was determined by 2 imaging- and 1 spectroscopy-based technique as previously published (72).

Incisional adipose tissue biopsies were obtained from affected (atrophic) and unaffected (preserved or hypertrophic) subcutaneous regions by a plastic surgeon. After sterile preparation and local anesthesia with 1% xylocaine, a skin incision was made, and adipose tissue was excised for subsequent analyses. Incisions were sutured and participants observed in the clinic for 1 hour.

### Bulk RNA-Seq from human and mouse WAT.

For human tissue, RNA was isolated from 150–200 mg adipose tissue using the RNeasy Lipid Tissue Mini Kit (QIAGEN, 74804). For mouse tissue, total RNA was extracted from WAT using RNA STAT-60 (amsbio, CS-110). Following DNase treatment, RNA underwent quality control, library preparation, and strand-specific mRNA sequencing (Beijing Genomics Institute). Over 20 million paired-end 100 bp reads were generated on the DNBSEQ platform.

Read quality was assessed with FastQC (v0.12.1) and alignment performed using STAR (v2.7.11a) with the UCSC mm39 reference genome. Postalignment quality control via FastQC ensured only high-quality data were used for differential expression analysis via DESeq2. Data visualization employed native DESeq2 functions, ggplot2, plotly, and related R packages.

Pathway analysis was conducted on ranked log_2_FC lists using GSEA (v4.3.3) and the Broad Institute MSigDB (v2024.1.Mm, M5 ontology sets). Enriched pathways were defined by FDR < 0.05, with the NES used to evaluate differential pathway magnitude (73). Plots were generated using ggplot2. All sequencing data are available through NCBI GEO.

### SnRNA-Seq from human adipose tissue.

Nuclei were isolated from 150–250 mg of tissue, minced in 1 mL nuclear extraction buffer (130-128-024, Miltenyi Biotec), and transferred into GentleMACS tubes (Miltenyi Biotec). The h_tumor_01 protocol was run 3 times on the GentleMACS, followed by 10 minutes on ice. The homogenate was passed through a 70 μm filter (Miltenyi) and washed with 3 mL nuclei extraction buffer. Samples were centrifuged at 500*g* for 10 minutes at 4°C, supernatant was removed, and the nuclear pellet was resuspended in 2 mL 1% BSA in PBS. Samples were filtered through a 30 μm filter, rinsed with 2 mL 1% BSA in PBS, and centrifuged again (500*g*, 10 minutes, 4°C). Pellets were resuspended in 500 μL 1% BSA in PBS and filtered through a prewet 20 μm filter, which was rinsed with 500 μL 1% BSA in PBS. After a final centrifugation (500*g*, 10 minutes, 4°C), nuclei were resuspended in 50 μL 1% BSA in PBS. Nuclei were counted at the University of Michigan Advanced Genomics Core using a Logos cell counter.

Probes were hybridized, and samples were pooled and processed per manufacturer instructions for the 10x Genomics Chromium Fixed RNA Profiling Reagent Kits for Multiplexed Samples (catalog 1000568). Library quality was assessed using the LabChip GXII HT (PerkinElmer) and quantified by Qubit (Thermo Fisher Scientific). Pooled libraries underwent paired-end sequencing on an Illumina NovaSeq XPlus. BCL Convert Software (Illumina) generated demultiplexed FASTQ files, and the Cell Ranger Pipeline (10x Genomics) was used for alignment and count matrix generation.

Seurat v5.1.0 was used for filtering, normalization, dimensional reduction, clustering, gene expression visualization, and differential expression analysis. Cells were excluded if they had <1,000 unique molecular identifiers (UMIs), <500 detected genes, >5% mitochondrial transcripts, or log_10_ genes per UMI < 0.80; genes were excluded if detected in <10 cells. SCTransform was used for normalization and variance stabilization, treating mitochondrial mapping percentage as a covariate. To integrate and correct for batch effects, Harmony was applied to the SCTransformed data via RunHarmony. Resulting embeddings were used for downstream dimensional reduction and clustering.

Thirty principal components were used for clustering and UMAP analysis, performed by a shared nearest neighbor modularity optimization-based algorithm. Clusters were visualized using UMAPs and annotated to cell types using known marker genes and top differentially expressed genes per cluster. Pathway analysis was conducted as described for bulk RNA-Seq.

### Animals.

*Lmna^fl/fl^* mice had *loxP* sites flanking exons 10 and 11 of the *Lmna* allele (74). Adipoq-CreERT2 mice (strain 025124) and mTmG mice (strain 007576) were from The Jackson Laboratory. Control animals were *Lmna^fl/fl^* and *Lmna*^iADKO^ mice were *Lmna^fl/fl^* *Adipoq*-Cre^ERT2+/–^. Animals described as *Lmna*^CTRL^ mTmG were *Adipoq*-Cre^ERT2+/–^ mTmG^+/–^, and *Lmna*^iADKO^ mTmG mice were *Lmna^fl/fl^* *Adipoq*-Cre^ERT2+/–^ mTmG^+/–^. Unless otherwise noted, 12- to 14-week-old mice received intraperitoneal tamoxifen (50 mg/kg; 13258, Cayman Chemical) in sterile corn oil for 5 consecutive days to induce recombination, followed by a 10-day washout. Mice were euthanized by inhaled isoflurane overdose, and death was confirmed by cervical dislocation or bilateral pneumothorax. Fat and lean mass were measured in live animals using an EchoMRI-100H. Animals were group-housed under standard conditions (22°C, 30%–60% humidity) with a 12-hour light/12-hour dark cycle and free access to water and chow. Daily care was overseen by the Unit for Laboratory Animal Medicine at the University of Michigan.

### Public data analysis from Lmna-KO MEFs.

Raw ATAC-Seq data were mined from GEO accession GSE120389 and processed using the best practice nf-core atac-seq pipeline (v2.1.1) (80) with default configurations. Microarray data were downloaded from GEO accession GSE124467 and preprocessed as described in its original publication (39).

### ATAC-Seq analysis and integration with expression data.

Differential accessibility analysis was performed using DiffBind (v3.12.0) (75). Briefly, a consensus peak set was generated, and read counting was done using dba.count() with bUseSummarizeOverlaps=TRUE. Trimmed mean of M-values and background-aware normalization was carried out with dba.normalize(). Enhancer-to-gene assignments were predicted using rGREAT (v2.6.0) (76). Chromatin state annotations for wild-type and KO peak sets were quantified by intersecting with the full-stack 100-segment ChromHMM model from Vu and Ernst 2023 (41) and visualized as grouped percentage bar plots. Genes and enhancers with concurrent chromatin and gene expression changes were stratified into 8 directionality-based clusters (e.g., increased accessibility with increased expression). GO enrichment was performed and visualized using clusterProfiler (v4.12.6) (77). For transcription factor motif analysis, peaks were ranked and binned by log_2_FC, and motif enrichment was assessed with monaLisa (v1.15.0) (78) using position weight matrix from JASPAR2020. Motifs with |log_2_ enrichment| > 2 and −log_10_ adjusted *P* > 4 were deemed significant. ATAC-Seq tracks and enhancer–gene connections were visualized using pyGenomeTracks (v3.9.0) (79).

### Statistics.

Data are shown as mean ± SD. Two-way ANOVA with Bonferroni’s post hoc test was used for human matched case-control comparisons and mouse data with 2 variables, 1-way ANOVA with Bonferroni’s post hoc test for human comparisons across disease states, and Student’s 2-tailed *t* test for 2-genotype mouse comparisons. AUC for lipid and glycemic parameters was calculated by the linear trapezoidal method. All analyses used GraphPad Prism v10, with *P* < 0.05 considered significant.

### Study approval.

The study was approved by the University of Michigan IRB (HUM 00174659); all participants provided written informed consent. Identifying tattoos in patient photos were removed with Adobe Photoshop’s Generative Fill. Written informed consent was received for the use of patient photographs. The University of Michigan approved animal studies under protocol PRO00011544 according to IACUC policies.

### Data availability.

snRNA-Seq and bulk RNA-Seq data are available through NCBI GEO: adipose from patients with FPLD2, bulk RNA-Seq, GSE311533; adipose from patients with FPLD2, snRNA-Seq, GSE310542; and *Lmna*^iADKO^ mouse adipose tissue, bulk RNA-Seq, GSE311534. Raw data values are provided in the Supporting Data Values file. Additional methods and materials are available in the Supplemental Methods.

## Author contributions

JNM, RLS, CNL, SM, EAO, and OAM conceived the studies and planned the experimental design. JNM, RLS, AN, MFF, PC, BNO, MN, MDM, DG, OB, BD, REA, ADG, MCG, HM, RMU, ZL, HH, IDKH, YC, LZ, APS, KSH, KTL, and TAH performed the experiments. JNM, RLS, AN, MFF, BNO, MN, MDM, DG, OB, BD, ADG, CNL, SM, EAO, and OAM analyzed the data. JNM and OAM wrote the manuscript, while all other authors edited and approved the final manuscript.

## Funding support

This work is the result of NIH funding, in whole or in part, and is subject to the NIH Public Access Policy. Through acceptance of this federal funding, the NIH has been given a right to make the work publicly available in PubMed Central.

- NIH to OAM and EAO (R01 DK125513), OAM (R01 DK137798, R01 DK121759), JNM (T32 HD007505; F31 DK135181), RLS (T32 DK101357; F32 DK123887), and KTL (T32 DK071212, F32 DK122654).
- Michigan Diabetes Research Center (MDRC) (P30 DK020572).
- Michigan Nutrition and Obesity Center (P30 DK089503).
- Michigan Integrative Musculoskeletal Health Core Center (P30 AR069620).
- Michigan Mouse Metabolic Phenotyping Center in Live Models (U2C-DK135066).
- MDRC Microscopy and Image Analysis Core.
- University of Michigan Bioinformatics, Microscopy, Flow Cytometry, Advanced Genomics, and Unit of Laboratory Animal Medicine Pathology Cores.
- Caswell Diabetes Institute Atypical Diabetes Program.
- University of Michigan Lipodystrophy Fund (gifted by Sopha, Baker, and Rosenblum families as well as the White Point Foundation of Turkey).

## Supplementary Material

Supplemental data

Unedited blot and gel images

Supporting data values

## Figures and Tables

**Table 1:**
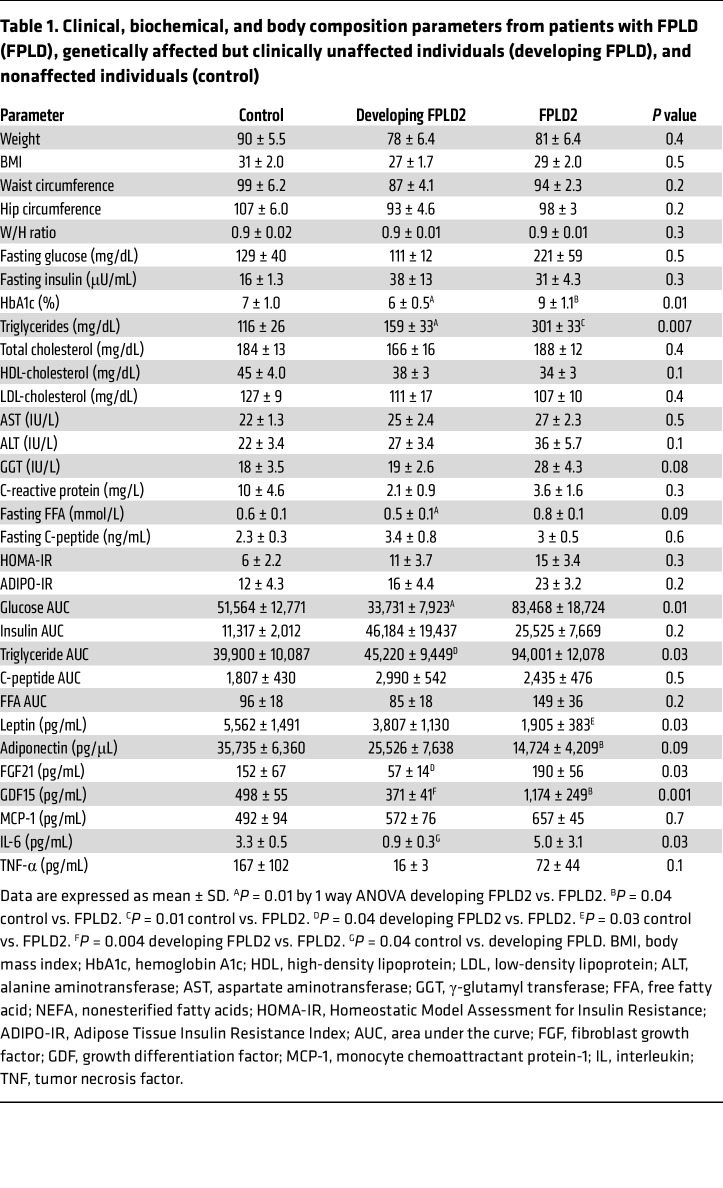
Clinical, biochemical, and body composition parameters from patients with FPLD (FPLD), genetically affected but clinically unaffected individuals (developing FPLD), and nonaffected individuals (control)

## Version 1 11/11/2025

In-Press Preview

## Version 2 01/02/2026

Electronic publication
