# Supplementary material for: Altered lipid metabolism and inflammatory programs associate with adipocyte loss in familial partial lipodystrophy 2
Source: J Clin Invest. 2025 Nov 11;136(1):e198387. doi: 10.1172/JCI198387 (PMC12721891; doi:10.1172/JCI198387)
Supplement: Supplemental data [file jci-136-198387-s287.pdf]

Supplemental Materials & Methods

Remove unwanted variation (RUV)-seq analysis on human WAT

To reduce the impact of both known and unknown confounding factors in the human samples, we incorporated known covariates into the DESeq2 model and accounted for hidden sources of variation using the RUVseq latent variable method (73). The analysis followed a multi-step approach: (1) an initial differential expression (DE) analysis was conducted using DESeq2, with RIN and fasting insulin included as known covariates. Genes that were not significantly different between conditions (p-value > 0.05) in this analysis were considered stable and used as empirical control genes in the RUVg function from RUVseq to estimate latent factors representing unmodeled variability. (2) These latent variables were then added to the DESeq2 model as additional covariates (alongside RIN and fasting insulin, where applicable) for a second round of DE analysis. The number of latent factors was chosen based on optimal separation of conditions and minimal distortion in relative log expression (RLE) plots. The final list of differentially expressed genes for each cell type was generated by applying a 5% false discovery rate (FDR) threshold to the output of the second DESeq2 run.

Quantification of human adipocyte size and fibrosis

Human adipose tissue sections were stained with hematoxylin & eosin and analyzed using the Aivia AI-based image analysis software (Leica Microsystems). Pixel Classifier profiles were trained to identify adipocytes. After optimization, adipocytes were counted, and area was quantified with Aivia's Recipe Console tool. At least 100 adipocytes were quantified per patient, with a median of 553 adipocytes measured per patient (1).

Glucose and insulin tolerance tests

For glucose tolerance tests, mice were fasted overnight (16 hours) and administered an intraperitoneal injection of glucose in water (1 mg/kg body weight). For insulin tolerance tests,

mice were fasted 4 hours and administered an intraperitoneal injection of insulin in saline (0.75 U insulin/kg body weight) (Eli Lilly). Blood was collected from the tail vein, and glucose concentrations were monitored using a glucometer and blood glucose strips (Bayer AG).

mTmG imaging and analyses

Tissues were collected from mice and placed in light-protected tubes with cold PBS on ice before imaging. Whole mount tissues were imaged in an imaging chamber in 1x PBS with an inverted confocal microscope (Nikon A1si) and 20x oil objective. mTmG images were analyzed using Fiji and the Cell Counter plugin to count red (tdTomato+) and green (GFP+) adipocytes. At least 300 adipocytes were counted over four to seven images per individual mouse.

Lipolysis measurements *in vivo*

Mice were bled in fed or fasted (~12-16 hours) states at baseline and after injection with tamoxifen. Blood was collected from the tail vein, allowed to coagulate on ice for 2 hours, and was centrifuged at 2000 x g for 20 minutes at 4°C to collect serum. For stimulated lipolysis, mice were administered an intraperitoneal injection of saline (control) or 10 mg isoproterenol/kg body weight to stimulate lipolysis. Blood was collected at baseline, and 30 and 60 minutes after injection, and serum isolated as described. Serum glycerol concentrations were measured via colorimetric assay (F6428, Sigma-Aldrich).

Serum measurements

Blood was collected from tail vein or at sacrifice via inferior vena cava and was allowed to coagulate on ice for 2 hours. After centrifugation at 2000 x g for 20 minutes at 4°C, serum was collected and stored at -80°C. ELISA was used to measure circulating concentrations of leptin

(900-K76K, Invitrogen), and triacylglycerols were measured via colorimetric assay (10010303, Cayman Chemical). Serum adiponectin concentrations were measured via immunoblot.

Mouse and human histology

After collection, adipose tissue, skin, and/or liver were fixed for 72 hours in 10% neutral-buffered formalin at 4°C. Tissues were rinsed with PBS and dehydrated in a graded series of ethanol washes, processed, paraffin-embedded through the University of Michigan Orthopedic Research Laboratory Histology Core facility, and sectioned at 5 μM thickness. Sections were stained with hematoxylin & eosin as previously described(75) or with Picrosirius Red according to manufacturer protocol (24901, Polysciences). Sections were imaged using an inverted Zeiss microscope with a 20x objective.

Immunoblot analyses

Immunoblot analyses were performed as previously described (2).

Adipocyte fractionation for immunoblot and recombination analyses

WAT depots were excised from mice, minced with scissors, and digested with 533.3 U/mL collagenase type I (LS004197, Worthington). Digestion was done in Krebs-Ringer-HEPES (KRH) buffer, pH 7.4 containing 1 g/L glucose and 500 nm adenosine for 30 minutes at 37°C shaking horizontally at 100 rpm. Adipocytes were separated from the stromal vascular fraction by filtering through 300 μm filters and centrifuging at 100 x g for 7 minutes. Floating adipocytes were transferred to 5 mL tubes and washed 3 times with KRH buffer by removing the infranatant from beneath the adipocyte layer with a 5 mL syringe and 21G needle. The stromal vascular fraction (SVF) was pelleted by centrifugation at 500 x g for 7 minutes and washing 3 times with KRH buffer. Adipocyte and SVF fractions were then lysed for either protein or DNA collection.

Adipocyte fractionation for respirometry analyses

Adipocyte fractionation was done as described above, except that collagenase digestion was done in Hank's Balanced Salt Solution (HBSS) (14025-092, Gibco). Adipocyte fractions were washed 3 times with STE-BSA buffer (250 mM sucrose, 5 mM Tris, 2 mM EGTA, 4% fatty acid free BSA, pH 7.4). After isolation, adipocyte metabolism was analyzed with an Oroboros-2k.

Respirometry of adipocytes

Oxygen consumption of adipocytes was measured with high-resolution respirometry using an Oxygraph-2k (OROBOROS Instruments, Innsbruck, Austria). Floated adipocytes (40 µL) from pmWAT were pipetted into 2 mL mitochondrial respiration medium buffer (MIR05) with the stirrer set to 750 rpm. The following respiratory parameters were measured: basal respiration (5 mM pyruvate, 1 mM malate, 5 mM succinate), proton leak respiration (1 µM oligomycin), and maximal respiration (1 µM FCCP steps until no further increase in oxygen consumption was observed). Non-mitochondrial oxygen consumption (2.5 µM antimycin A) was subtracted from the other respiratory states. ATP-linked respiration was calculated by subtracting leak respiration from baseline, and spare capacity was calculated by subtracting baseline from maximal respiration.

DNA content of adipocyte suspensions was quantified by quantitative PCR targeting genomic DNA using primers for glucagon. Samples were diluted 1:5 in digestion buffer (50 mM KCl, 0.45% NP-40, 0.45% Tween-20, 0.2 mg/mL Proteinase K) and incubated at 65°C for 1 hour at 1000 rpm. Proteinase K was inactivated at 65°C for 10 minutes, and 2.25 volumes phenol:chloroform:isoamyl alcohol were added. Samples were centrifuged at 16000 x g for 10 minutes, and the aqueous phase was collected and used for qPCR (StepOnePlus System, Applied Biosystems) using qPCRBIO SyGreen Mix (PB20.12-01, Innovative Solutions). DNA

concentrations were determined by comparing samples with a mouse genomic DNA standard dilution series of known concentration.

Quantitative RT-PCR

M-MLV Reverse Transcriptase (Invitrogen, Carlsbad, CA, USA) was used to transcribe 1 μg RNA to cDNA. DNA was amplified with qPCRBIO SyGreen Mix (Innovative Solutions, Beverly Hills, MI, USA) and detected with a StepOnePlus System (Applied Biosystems, Foster City, CA, USA). All primers were validated with cDNA titration curves prior to use; qPCR product specificities were confirmed by melting curve analysis and gel electrophoresis. Gene expression was calculated using a cDNA titration curve within each plate and normalized to the geomean of reference genes *Tbp, Gapdh,* and *Hprt*, and calculated as mRNA transcript change relative to control samples.

Quantification of mitochondrial DNA

DNA was isolated from floated adipocytes using the Wizard Genomic DNA Purification Kit (A1120, Promega). DNA amount was quantified using a NanoDrop spectrophotometer. DNA was amplified using RT-qPCR with primers identifying nuclear genes *Gcg* and *Hbb*, or mitochondrial-encoded genes *Mt-Cyb* and *Mt-Cox1-Tms1*.

MitoTracker staining of primary adipocytes and imaging

Primary adipocytes were isolated using the protocol for respirometry analyses. Adipocytes were then stained in PBS with BODIPY 493/503 for 30 minutes (D3922, Invitrogen), Hoechst 33342 for 10 minutes (62249, ThermoFisher), and MitoTracker Deep Red FM for 30 minutes (M22426, Invitrogen) and washed with PBS twice. Adipocytes were imaged using an inverted spinning disk confocal (Nikon W1-SoRa).

Flow cytometry analysis of SVF for delipidated adipocytes

SVCs were isolated from psWAT and eWAT as described above from Adipoq-CreERT2$^{+/-}$ mTmG$^{+/-}$ or *Lmna*$^{fl/fl}$ Adipoq-CreERT2$^{+/-}$ mTmG$^{+/-}$ mice. Cells were kept on ice in the dark, stained with propidium iodide (P1304MP, Invitrogen) and immediately brought to the flow cytometer (5-Laser Cytek Aurora Spectral Analyzer with autofluorescence subtraction) for analysis of fluorescence (GFP vs. tdTomato positive) in SVF cells. Single stained ear mesenchymal stem cells and unstained SVF were used as controls.

Flow cytometry analysis of SVF for inflammation

PmWAT and psWAT depots were collected and weighed. For isolation of SVF, minced WAT was digested with 1 mg/ml collagenase (Type II, C2-BIOC, Sigma–Aldrich) in HBSS (-/-) (14175095, Invitrogen), and tissues were incubated at 37°C for 30 minutes with constant shaking. Cell suspensions were passed through a 100 µm filter and centrifuged at 500 x g for 10 minutes at 4°C to separate floating adipocytes from pelleted SVF. For flow cytometry, SVF were incubated in 0.5 ml red blood cell lysis buffer (155mM NH_4Cl, 10mM $KHCO_3$, 0.1mM EDTA, 1N HCl) for 5 minutes at room temperature and resuspended in PBS/0.5% BSA prior to incubation in Fc Block (anti-CD16/32) for 5 minutes on ice. For extracellular staining (30 minutes), cells were incubated at 4°C with antibodies to CD45, CD64, CD11c, TIM4, and CD163 for ATMs and to CD3, CD4, and CD8 for T cells. The following antibodies were used: anti-CD45 (clone 30-F11), anti-CD64 (X54-5/7.1), anti-cd11c (N418), anti-CD163 (S15949F), anti-TIMD4 (RMT4-54), anti-cd3 (145-2C11), anti-cd4 (RM4-5) and anti-cd8 (53-6.7). Stained cells were washed twice with FACS buffer and fixed in 2% paraformaldehyde. After separation with a Cytek Aurora flow cytometer, data were analyzed using manual compensation and FlowJo software.

Transmission electron microscopy (TEM) of mouse WAT

Mice were anesthetized and pmWAT immediately dissected, cut into 1 mm^3 pieces and placed into 2.5% glutaraldehyde, 2% formaldehyde in 0.1M sodium cacodylate buffer (CB) at 4°C for 2 hours. Samples were washed 3 times with 0.1 M CB for 15 minutes. Then samples were placed into 1.5% potassium hexacyanidoferrate(II) + 2% osmium tetroxide in 0.1 M CB for 1 hour. Samples were then washed 3 times with 0.1 M CB for 5 minutes and washed 3 times with 0.1 M acetate buffer (AB) for 5 minutes. Samples were placed into 2% uranyl acetate in 0.1 M AB for 1 hour. Samples were washed twice with 0.1 M AB for 5 minutes and washed with deionized water for 5 minutes. After a dehydrated series of 30, 50, 70, 80, 90, 95, and 100% ethanol for 15 minutes each at 4°C, samples were placed in acetone for 15 minutes at 4°C, 2:1 acetone:resin for 1.5 hours, 1:1 for 3 hours, and 1:2 for 16 hours. Samples were embedded in 100% resin under vacuum for 48 hours, and resin polymerized at 70°C for 24 hours. Sections of 70 µm were imaged on a JOEL JEM-1400 Plus LaB6 transmission electron microscope.

TEM image analysis for general mitochondria morphology

Mitochondrial morphology was quantified in Fiji/ImageJ following the workflow of Lam et al. 2021 with minor adaptations (3). Briefly, 8-bit images were used and pixel size was calibrated from scale bars (Set Scale). For each image, mitochondria were manually outlined using the Freehand Selection tool to create per-mitochondrion ROIs (ROI Manager). Within each ROI we measured cross-sectional area, perimeter, Feret's diameter, circularity, and aspect ratio (Analyze → Measure). To minimize bias from neighboring structures, ROIs excluded outer-membrane overlaps with adjacent organelles. Contrast was standardized across images (Process → Enhance Contrast; saturated = 0.2–0.5%), and no deconvolution or non-linear filtering was applied. Measurements were exported from the Results table as CSV for downstream statistics.

Cristae orientation anisotropy analysis (manual, line-based)

Cristae parallelism was quantified as an axial anisotropy index computed from manually traced cristae orientations. In Fiji, the Straight-line tool was used to draw lines along the long axis of clearly resolved cristae within each mitochondrion ROI; each line was added to the ROI Manager (shortcut: t). For each line, Fiji's Measure function returned the angle (degrees relative to the x-axis). Angles were exported (Results → Save As) and analyzed in R (v. ≥4.0). Because orientations have 180° symmetry (a line at 0° is equivalent to 180°), we used the axial (doubled-angle) circular statistic to compute anisotropy as the axial mean resultant length, following standard circular statistics approaches (4, 5).

$$r_{\text{axial}} = \sqrt{\left(\frac{1}{N}\sum_i \cos(2\theta_i)\right)^2 + \left(\frac{1}{N}\sum_i \sin(2\theta_i)\right)^2}$$

where θ_i are cristae angles in radians and N is the number of traced cristae. r_{axial} ranges from 0 (random orientations) to 1 (perfectly parallel). The corresponding mean axial orientation was reported modulo 180°. For each mitochondrion, we traced ≥10 cristae whenever possible; when cristae were sparse or oblique, we traced all discernible cristae and noted the count. We report r_{axial} per mitochondrion (mean of cristae within that mitochondrion).

R Equation code for anisotropy analysis

```
# Axial anisotropy (0..1) and mean axial orientation (deg, modulo 180)
anisotropy_axial <- function(angles_deg) {
  th <- angles_deg * pi/180
  cx <- mean(cos(2*th)); sy <- mean(sin(2*th))
  r  <- sqrt(cx^2 + sy^2)
  mu <- atan2(sy, cx)/2 * 180/pi
  mu <- (mu + 180) %% 180
```

```
  list(r = r, mean_deg = mu, n = length(angles_deg))
}
```

Tandem mass tag proteomics from mouse adipose tissue

Protein digestion and TMT labeling and liquid chromatography-mass spectrometry analysis was performed as previously described (6). Data were analyzed with Proteome Discoverer (v2.4; ThermoFisher). MS2 spectra were searched against SwissProt mouse protein database (#55336 entries, Mus musculus ALL_UniProtKB 2021_03.fasta, downloaded on 08/06/2021) using the following search parameters: MS1 and MS2 tolerance were set to 10 ppm and 0.6 Da, respectively; carbamidomethylation of cysteines (57.02146 Da) and TMT labeling of lysine and N-termini of peptides (229.16293 Da) were considered static modifications; oxidation of methionine (15.9949 Da) and deamidation of asparagine and glutamine (0.98401 Da) were considered variable. Identified proteins and peptides were filtered to retain only those that passed ≤1% FDR threshold. Quantitation was performed using high-quality MS3 spectra (Average signal-to-noise ratio of 10 and <50% isolation interference).

Primers for quantitative RT-PCR

Gene symbol	Forward primer (5’-3’)	Reverse primer (5’-3’)
Gcg	AGGGCCATCTCAGAACC	GCTATTGGAAAGCCTCTTGC
Hbb	GAAGCGATTCTAGGGAGCAG	GGAGCAGCGATTCTGAGTAGA
Mt-Cyb	CATTTATTATCGCGGCCCTA	TGTTGGGTTGTTTGATCCTG
Mt-Cox1-Tms1	GCCTTTCAGGAATACCACGA	CCAATTTTAGGGGGTTCGAT
Gapdh	ATGTTCCAGTATGACTCCACTCACG	GAAGACACCAGTAGACTCCACGACA
Hprt	TCATTATGCCGAGGATTTGGA	GCACACAGAGGGCCACAAT
Tbp	ACCTTATGCTCAGGGCTTGG	GCCGTAAGGCATCATTGGAC
Scd1	GATAGAGCAAGTCCCCGCTG	CCTGCATTAACCCCCTTCAC
Adipoq	CATTCCGGGACTCTACTACTTCT	GAGGCCTGGTCCACATTCTT
Il6	CCAGAGATACAAAGAAATGAT	ACTCCAGAAGACCAGAGGAAAT
Il10	GAGGCGCTGTCATCGATTT	CACCTTGGTCTTGGAGCTTATT
Saa3	CGCAGCACGAGCAGCAGGAT	TGGCTGTCAACTCCCAGG
Tgfb1	TTGCTTCAGCTCCACAGAGA	TGGTTGTAGAGGGCAAGGAC
Ccl2	CTTCTGGGCCTGCTGTTCA	CCAGCCTACTCATTGGGATCA
Nos2	AATCTTGGAGCGAGTTGTGG	CAGGAAGTAGGTGAGGGCTTG
Nlrp3	CCGTCTACGTCTTCTTCCTTTC	CGCAGATCACAGTCCTCAAATA
Il1b	GAGGACATGAGCACCTTCTTT	GCCTGTAGTGCAGTTGTCTAA
Casp1	GGCCCCAGGCAAGCCAAATCT	CAGTCCTGGAAATGTGCCATC
Tnfa	CATCTTCTCAAAATTCGAGTGACAA	TGGGAGTAGACAAGGTACAACCC

Antibodies for immunoblot

Antibody Name	Manufacturer	Catalog #
Adiponectin	Sigma-Aldrich	A6354
Albumin	ThermoFisher	PA5-27707
SREBP1	ThermoFisher	PA1-337
ChREBP	Novus Bio	NB400-135
ACC	Cell Signaling	3662
FASN	Abcam	ab22759
SCD1	Cell Signaling	2438
Laminin	Novus Bio	NB300-144
PPARγ	Millipore Sigma	MAB3872
LMNA	Cell Signaling	4777
C/EBPα	Cell Signaling	8178
ACAA2	Abcam	ab128911
OXPHOS Rodent Antibody Cocktail	Abcam	ab110413
HADHB	Novus Bio	NBP1-54750
CPT1α	Abcam	ab128568
Pyruvate dehydrogenase	Cell Signaling	3205
Mitochondrial Dynamics Sampler Kit	Cell Signaling	74792
VDAC1	Abcam	ab15895
PDGFRβ	Proteintech	13449-1-AP
PGC1α	Abcam	ab191838
β-actin	Cell Signaling	4970
Cleaved caspase 3	Cell Signaling	9664
Total caspase 3	Cell Signaling	9662

Antibodies for flow cytometry

Marker	Fluorophore	Isotype	Clone	Product Info	Laser
CD45	Efluor 450	Mouse / IgG1, kappa	30-F11	ebio 45-0451-80	405
CD64	PE	Mouse (NOD/Lt) IgG1, κ	X54-5/7.1	BD pharmingen 558455	561
CD11c	APC-Cy7	Armenian Hamster IgG1, λ2	N418	Biolegend 117334	640
Tim4	PerCP-efluor 710	Mouse / IgG1, kappa	RMT4-54	Thermofisher 46-5866-82	488
CD163	PECy7	Rat / IgG2a, kappa	S15949F	Biolegend 156707	561
CD11b	BV711	Rat IgG2b, κ	M1/70	Biolegend 101242	405
CD3	APC	Armenian hamster / IgG	145-2C11	eBioscience 17-0031-82	640
CD4	BV650	Rat IgG2b, κ	RM4-5	Biolegend 100555	405
CD8	FITC	Rat / IgG2a, kappa	53-6.7	eBioscience 11-0081-85	488

References for Supplemental Materials and Methods

1. Parlee, S. D., et al. Quantifying size and number of adipocytes in adipose tissue. *Methods Enzymol.* 2014;537:93–122.

2. Bagchi, D. P. et al. Wnt/β-catenin signaling regulates adipose tissue lipogenesis and adipocyte-specific loss is rigorously defended by neighboring stromal-vascular cells. *Mol. Metab.* 2020;42:101078.

3. Lam J, et al. A Universal Approach to Analyzing Transmission Electron Microscopy with ImageJ. *Cells*. 2021;10:2177.

4. Fisher, N. I. Statistical Analysis of Circular Data. Cambridge, UK: Cambridge University Press; 1993.

5. Zar, J. H. Biostatistical Analysis (4th ed.). Upper Saddle River, NJ.: Prentice Hall; 1999.

6. Ewels, P. A. et al. The nf-core framework for community-curated bioinformatics pipelines. *Nat. Biotechnol.* 2020;38:276–278.

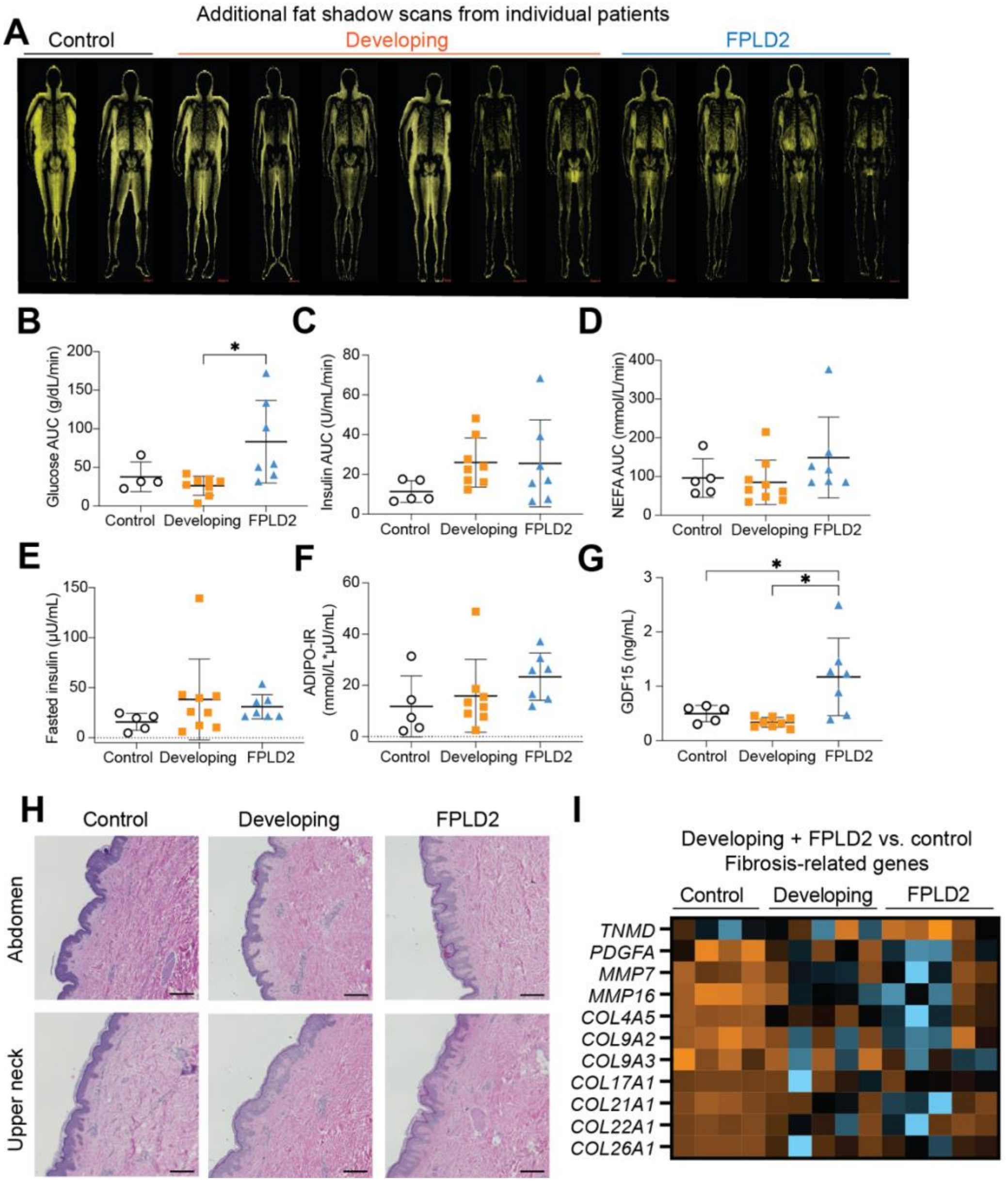

Supplemental Figure 1. Patients with FPLD2 have impaired glucose tolerance and increased GDF15 concentrations with otherwise comparable clinical characteristics to controls. (**A**) Additional fat shadow scans from all patients. (**B**) Area under the curve (AUC) from an oral glucose tolerance test (OGTT). (**C**) Insulin AUC during OGTT. (**D**) NEFA AUC during OGTT. (**E**) Fasted insulin concentrations. (**F**) Adipose tissue insulin resistance index (ADIPO-IR). (**G**) Growth differentiation factor 15 (GDF15) concentrations. (**H**) Histological images of skin from subcutaneous adipose tissue biopsies. (**I**) Heatmap of fibrosis-related

genes from bulk RNA-seq from human biopsies with accompanying z-scores. Scale bar = 50 μm.

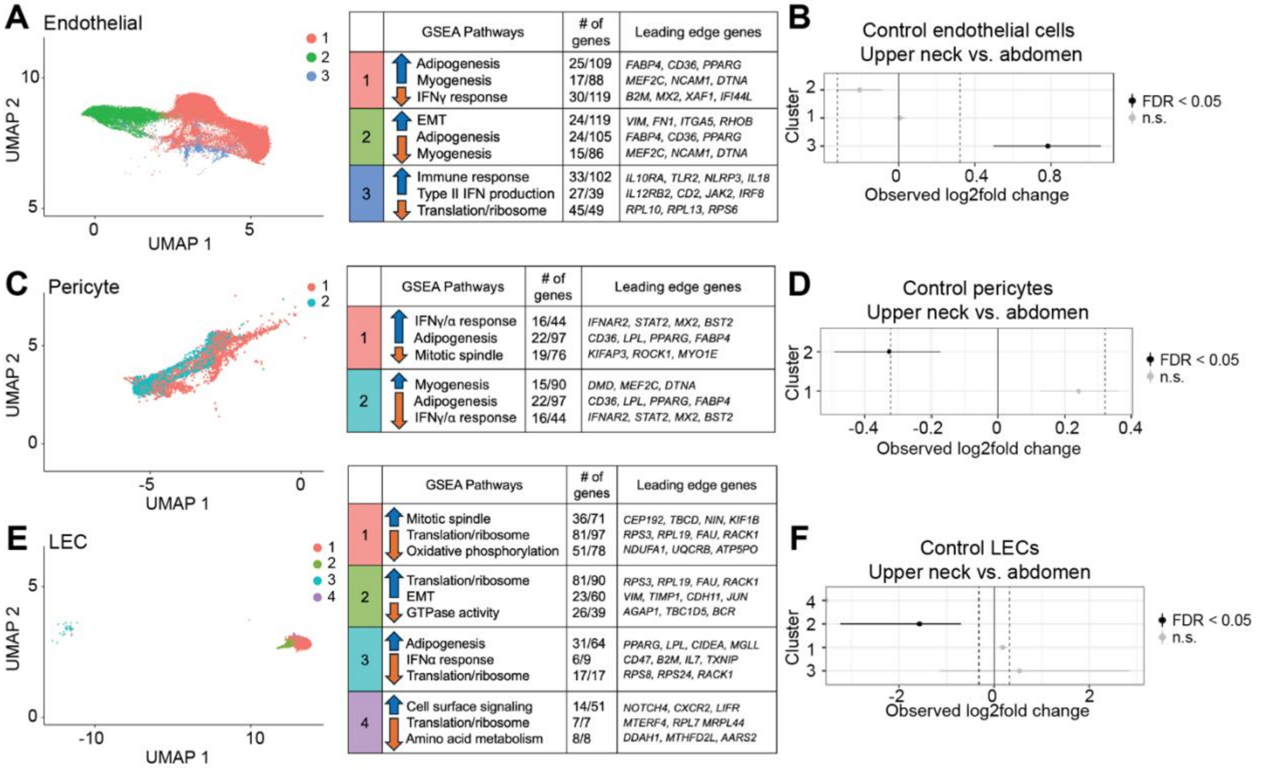

Supplemental Figure 2. Single nuclei RNA-sequencing identifies changes in cell proportions and clusters in upper neck and abdominal subcutaneous adipose tissue biopsies from control patients. (**A**) Endothelial subclusters with all disease states and biopsy location combined and their GSEA pathways. (**B**) Endothelial subcluster changes in the upper neck relative to abdomen in control biopsies. (**C**) Pericyte subclusters with GSEA pathways. (**D**) Subcluster changes in pericytes in the upper neck versus the abdomen. (**E**) LEC subclusters with GSEA pathways. (**F**) Subcluster changes in LECs in the upper neck relative to the abdomen.

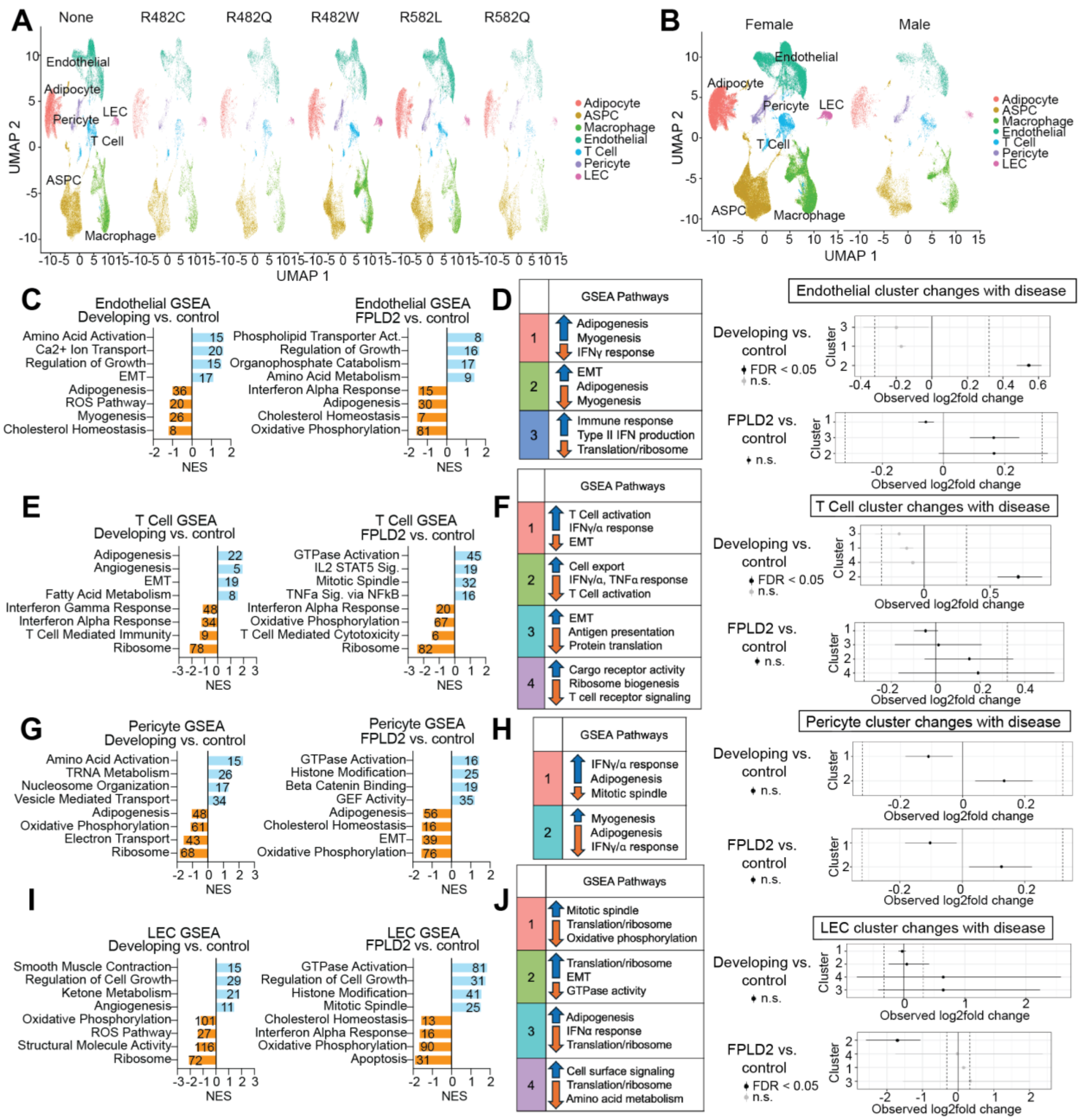

Supplemental Figure 3. Single nuclei analyses reveal cell proportion changes and identity with disease state. (**A**) UMAP projection of combined upper neck and abdomen biopsies from patients with different *LMNA* variants or separated by (**B**) female and male individuals. (**C**) All endothelial cells from developing or FPLD2 biopsies were analyzed via GSEA and compared to controls to identify population-level changes in cell identity. (**D**) Endothelial

subclusters with corresponding GSEA pathways and subcluster changes with disease. (**E**) T cell GSEA in developing or FPLD2 biopsies relative to controls. (**F**) T cell subcluster changes with disease. (**G**) Pericyte GSEA in developing or FPLD2 biopsies relative to controls. (**H**) Pericyte subcluster changes with disease. (**I**) LEC GSEA in developing or FPLD2 biopsies. (**J**) LEC subcluster changes with disease.

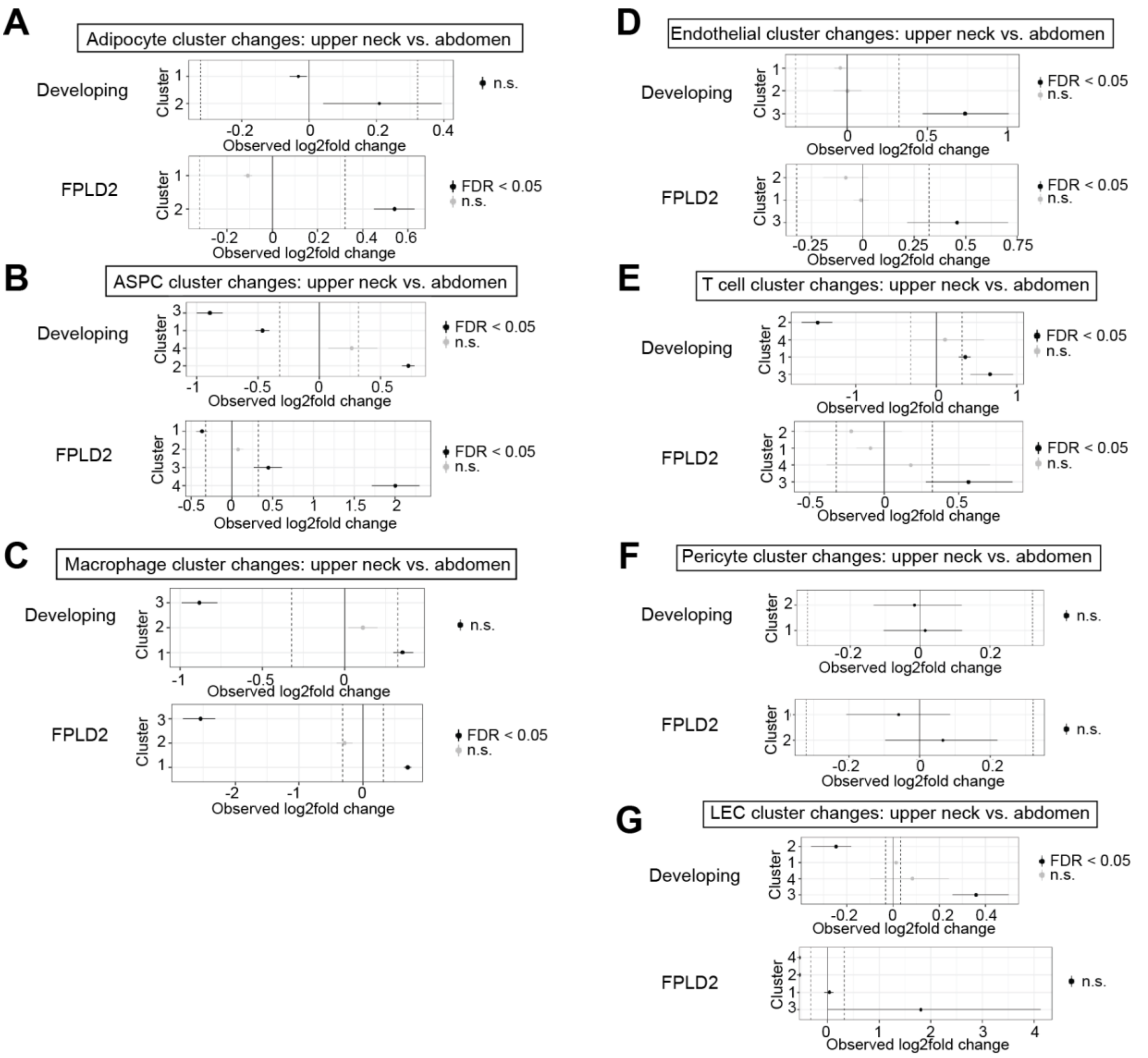

Supplemental Figure 4. Subcluster analyses reveal depot-specific shifts in cellular identity. Subcluster changes in the upper neck relative to the abdomen in biopsies from patients with developing and developed FPLD2 in (**A**) adipocytes, (**B**) ASPC, (**C**) macrophages, (**D**) endothelial cells, (**E**) T cells, (**F**) pericytes, and (**G**) LECs.

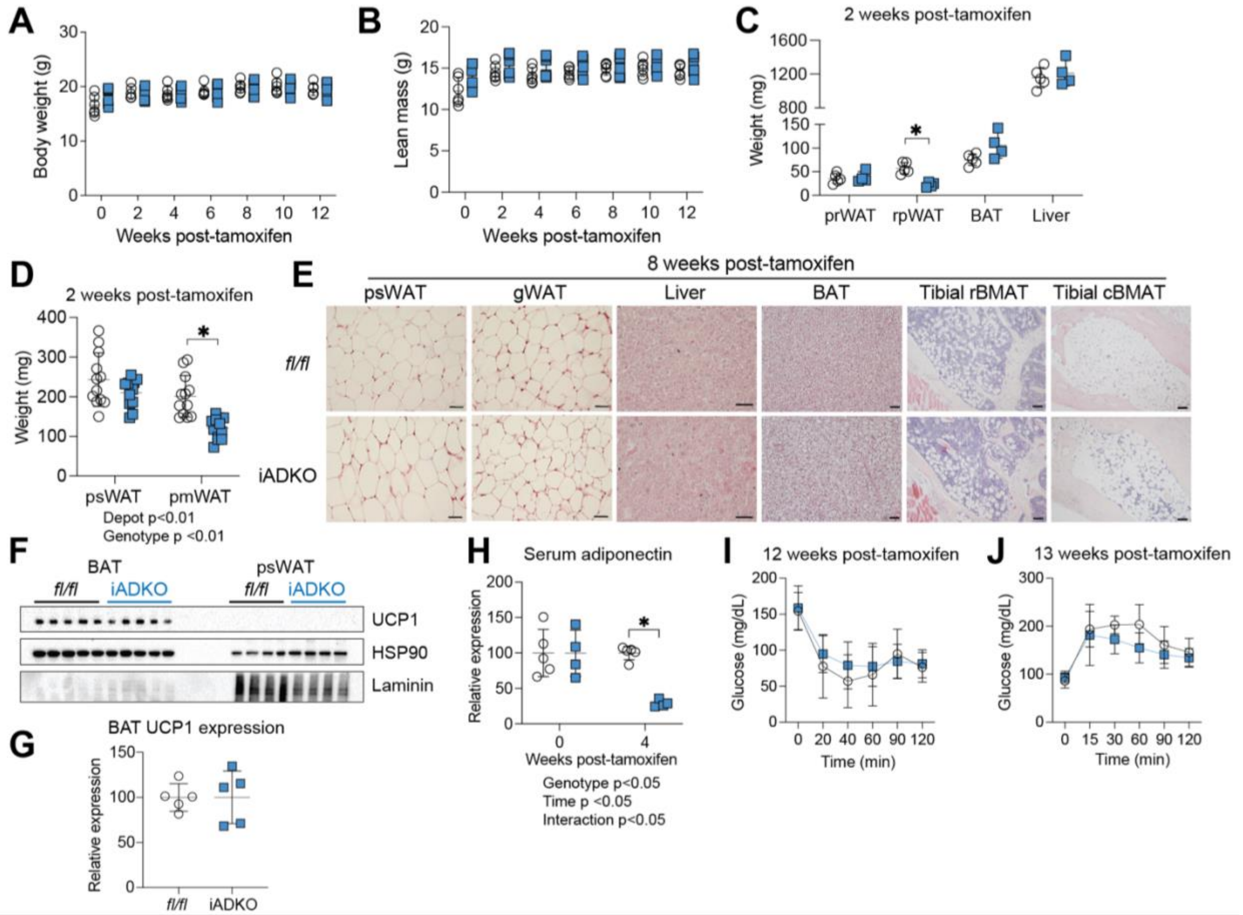

Supplemental Figure 5. *Lmna*^{iADKO} mice do not have changes in body weight, lean mass, or develop metabolic dysfunction. All data from male mice except histology from female mice in D. (**A**) Body weight and (**B**) lean mass post-tamoxifen over time (n = 6). (**C**) Weights of perirenal white adipose tissue (prWAT), retroperitoneal white adipose tissue (rpWAT), brown adipose tissue (BAT), and liver two-weeks post-tamoxifen (n = 6). (**D**) psWAT and pmWAT weights of female mice two-weeks post-tamoxifen (n = 12). (**E**) Histology of psWAT, gWAT, liver, BAT, and tibial regulated bone marrow adipose tissue (rBMAT) and constitutive bone marrow adipose tissue (cBMAT). Scale bar = 40 μm. (**F**) UCP1 expression in BAT and psWAT (n = 4-5) and (**G**) quantification of BAT UCP1 expression two weeks post-tamoxifen. Loading controls = HSP90 and laminin. Expression is normalized to HSP90. (**H**) Quantification of serum adiponectin immunoblot four-weeks post-tamoxifen (n = 4-5), expression normalized to serum

albumin. (**I**) Insulin tolerance test 12 weeks post-tamoxifen (n = 6). (**J**) Glucose tolerance test 13 weeks post-tamoxifen (n = 6). Data are represented as mean ± SD. *$P < 0.05$. Statistical analyses were performed using two-way ANOVA with a Bonferroni post-hoc test.

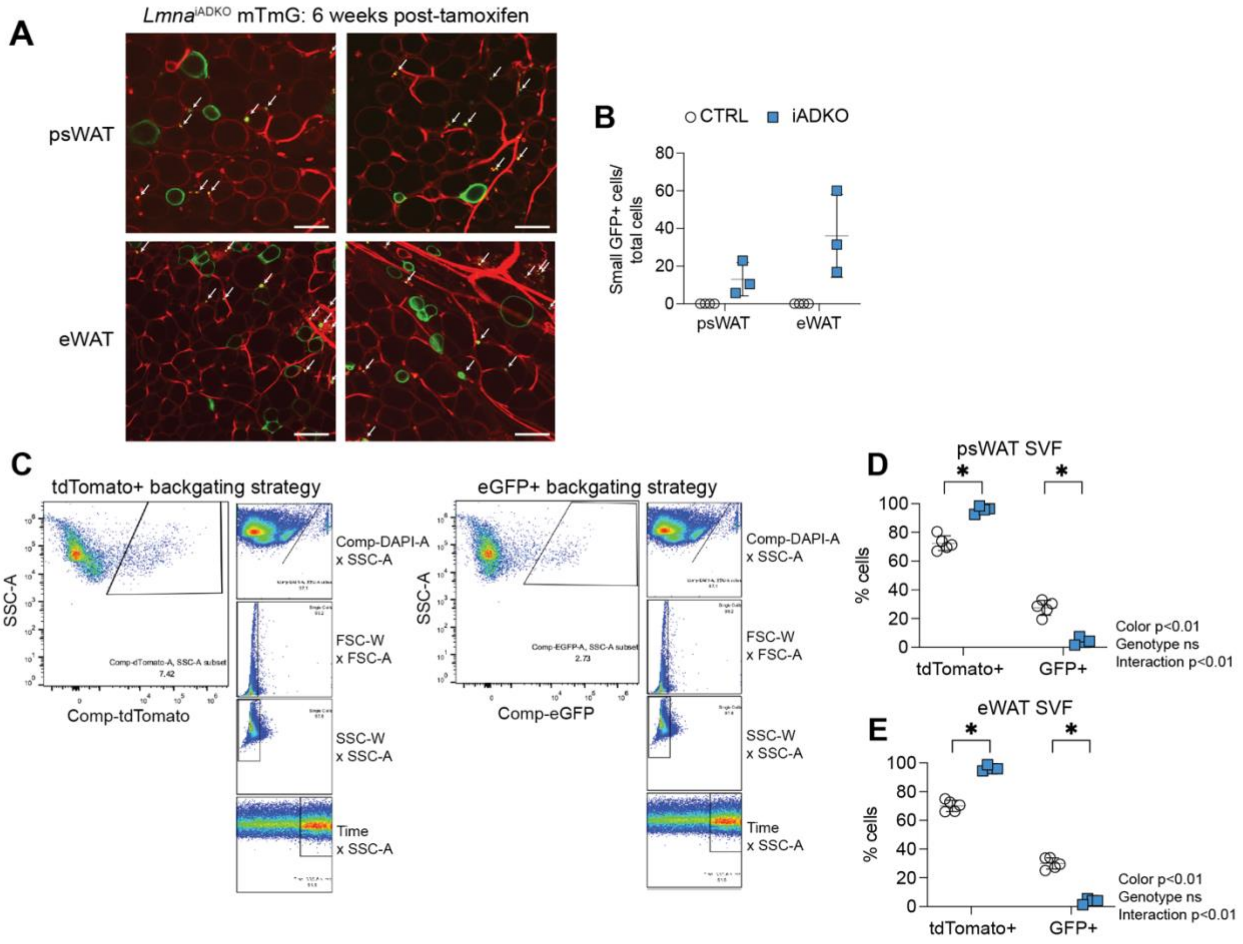

Supplemental Figure 6. *Lmna* knockout adipocytes do not de-differentiate and reside in adipose tissue after adipocyte shrinkage. All data from male mice. (**A**) Fresh confocal micrographs of small GFP-positive cells only present in *Lmna*^{iADKO} mTmG psWAT and eWAT six-weeks post-tamoxifen. Scale bar = 100 µm. (**B**) Quantification of confocal micrographs. (**C**) Backgating strategy to identify tdTomato+ and GFP+ stromal vascular cells (SVC) in *Lmna*^{iADKO} mTmG WAT six-weeks post-tamoxifen. Flow cytometry analyses of tdTomato+ and GFP+ SVCs in (**D**) psWAT and E, eWAT.

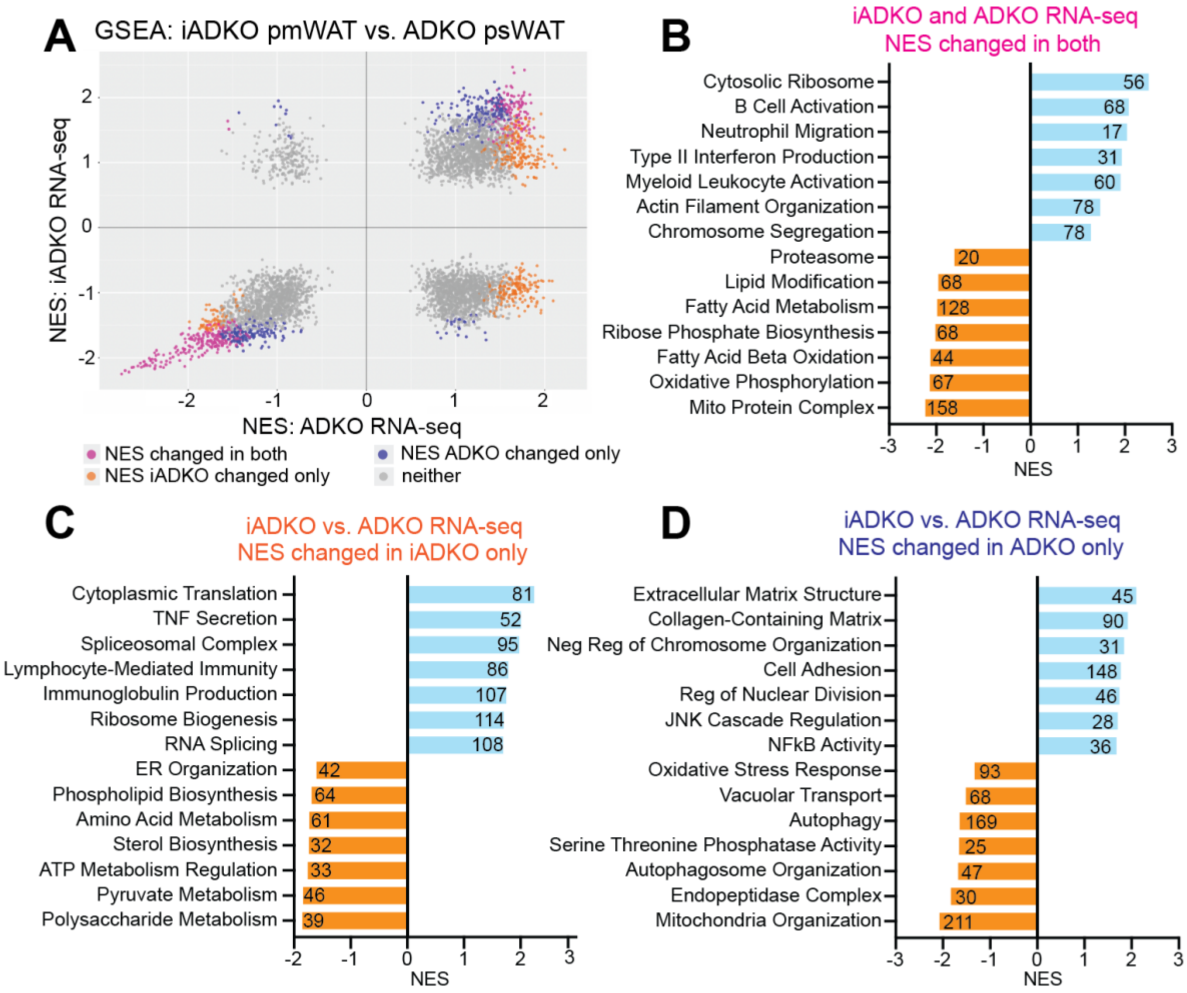

Supplemental Figure 7. Integrative RNA-seq analyses of *Lmna*^{iADKO} compared to *Lmna*^{ADKO} WAT reveals conserved patterns of gene expression across *Lmna* knockout models and depots. (**A**) Integrative GSEA comparison of RNA-seq *Lmna*^{iADKO} pmWAT two-weeks post-tamoxifen compared to *Lmna*^{ADKO} psWAT from four-week-old female mice (n = 5-6). Pink dots symbolize that NES were significantly changed in both inducible and constitutive datasets, orange dots = NES only changed in *Lmna*^{iADKO}, blue = NES only changed in *Lmna*^{ADKO}. (**B**) Overlapping GSEA pathways in both *Lmna*^{iADKO} and *Lmna*^{ADKO} RNA-seq. Numbers on bars represent number of overlapping genes driving pathways. GSEA pathways changed in only (**C**) *Lmna*^{iADKO} or (**D**) *Lmna*^{ADKO} WAT.

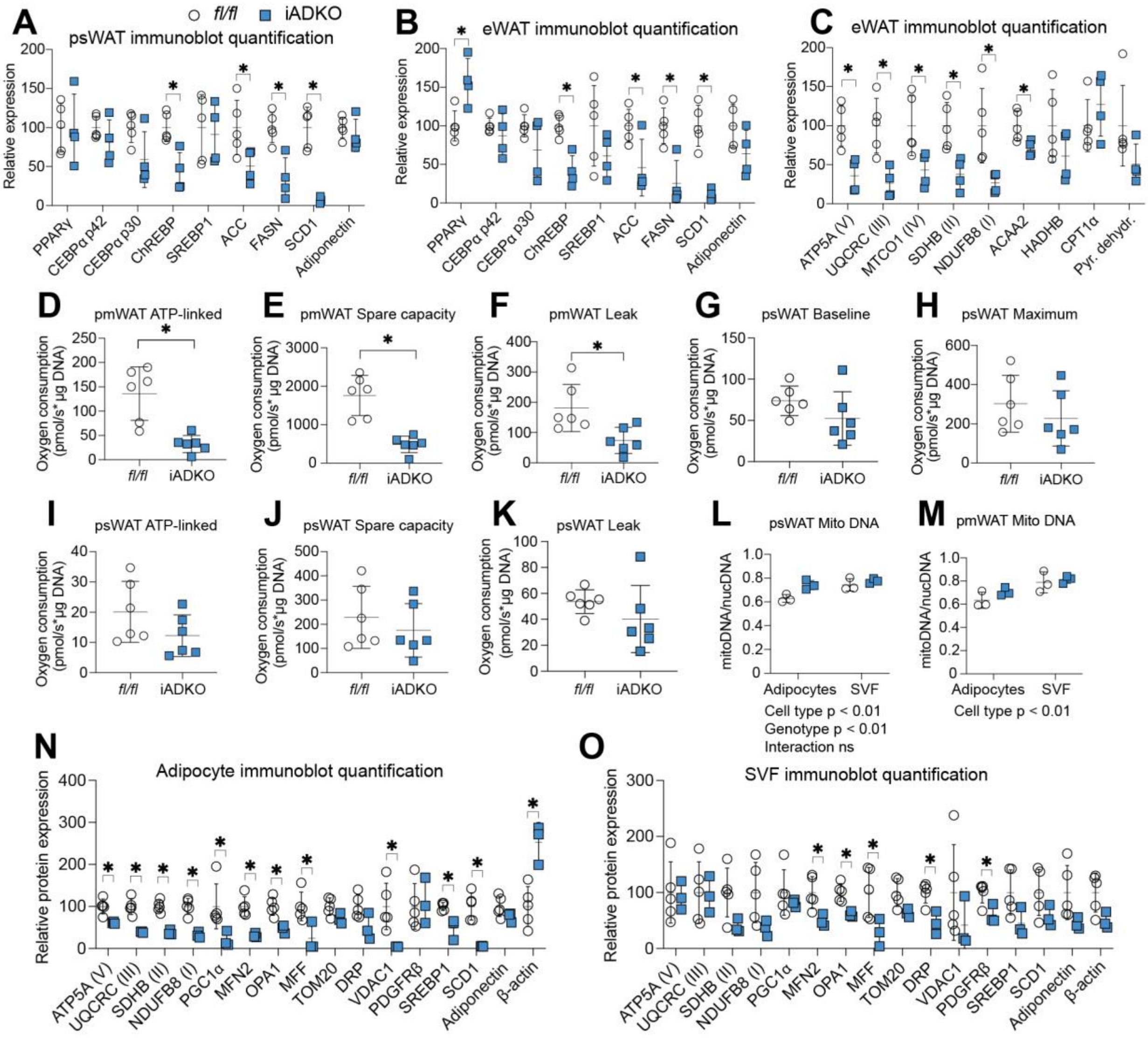

Supplemental Figure 8. Lipogenic and mitochondrial proteins are repressed in *Lmna*$^{\mathbf{iADKO}}$ WAT, and *Lmna* KO pmWAT adipocytes but not psWAT adipocytes have reduced respiration. Quantification of immunoblots from Fig. 7A-C for (**A**) psWAT lipid metabolism proteins, (**B**) eWAT lipid metabolism proteins, and (**C**) eWAT mitochondrial proteins two-weeks post-tamoxifen (n = 4-5). Oroboros Oxygraph 2k analyses of isolated adipocytes from pmWAT two-weeks post-tamoxifen of (**D**) ATP-linked respiration, (**E**) spare capacity, and (**F**) leak. Respiration analyses from psWAT adipocytes of (**G**) baseline respiration, (**H**) maximum respiration, (**I**) ATP-linked respiration, (**J**) spare capacity, and (**K**) proton leak (n = 6).

Mitochondrial DNA quantification for (**L**) psWAT and (**M**) pmWAT two-weeks post-tamoxifen (n = 3). Immunoblot quantification from (**N**) adipocytes and (**O**) SVF mitochondrial dynamic proteins from Fig. 7F (n = 3-5). Data are represented as mean ± SD. *P < 0.05. Statistical analyses were performed using two-way ANOVA with a Bonferroni post-hoc test.

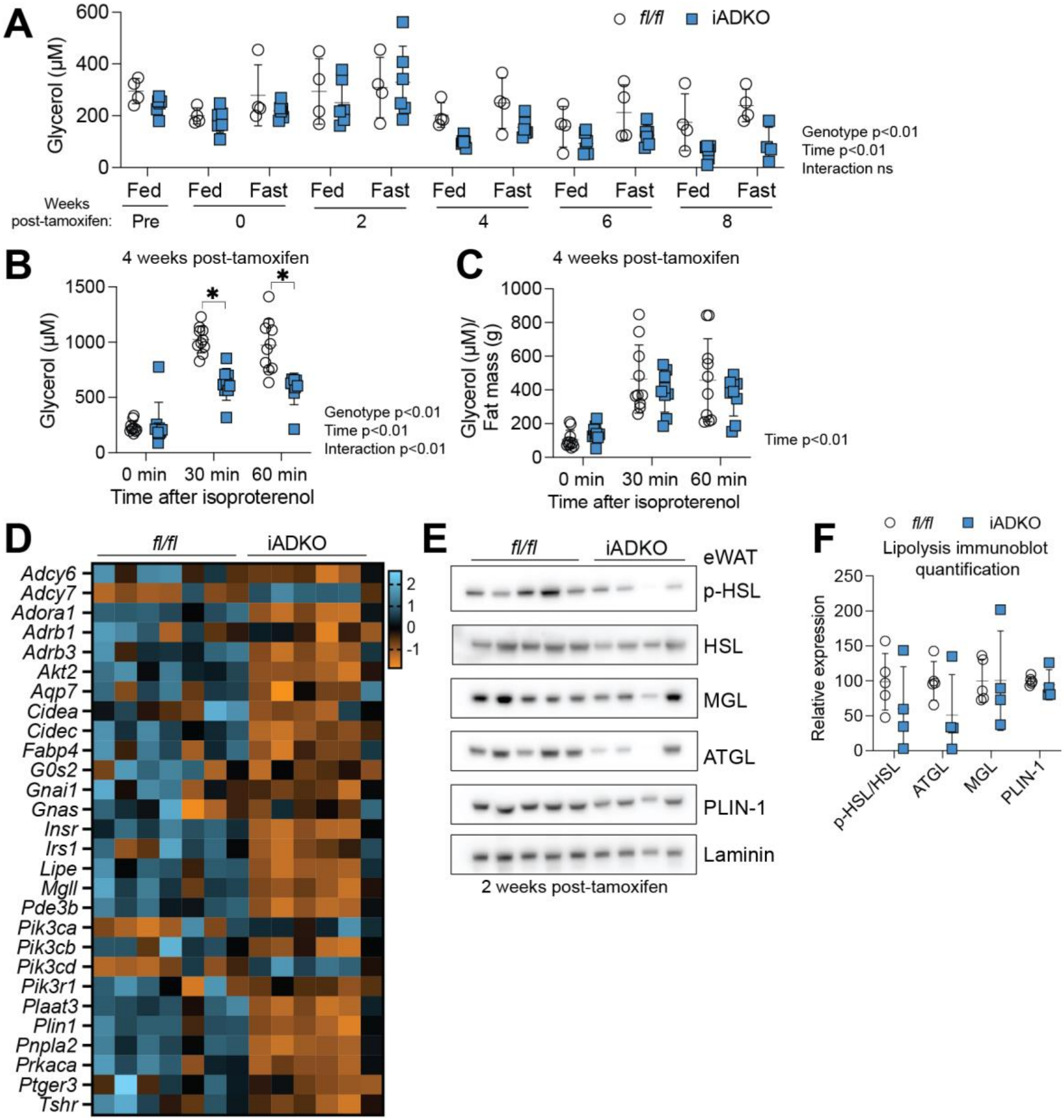

Supplemental Figure 9. Lipolysis is not increased in *Lmna*^{iADKO} WAT. (**A**) Circulating fasted and fed glycerol concentrations in male mice post-tamoxifen (n = 4-6). (**B**) Stimulated lipolysis assay following i.p. isoproterenol injection four-weeks post-tamoxifen (n = 9-10). (**C**) Glycerol concentrations from stimulated lipolysis assay normalized to fat mass. (**D**) Heatmap of lipolytic genes from *Lmna*^{iADKO} RNA-seq from pmWAT two-weeks post-tamoxifen. (**E**) Immunoblot of

lipolysis protein expression from eWAT two-weeks post-tamoxifen and (**F**) quantification of immunoblot (n = 4-5). Loading control = laminin.

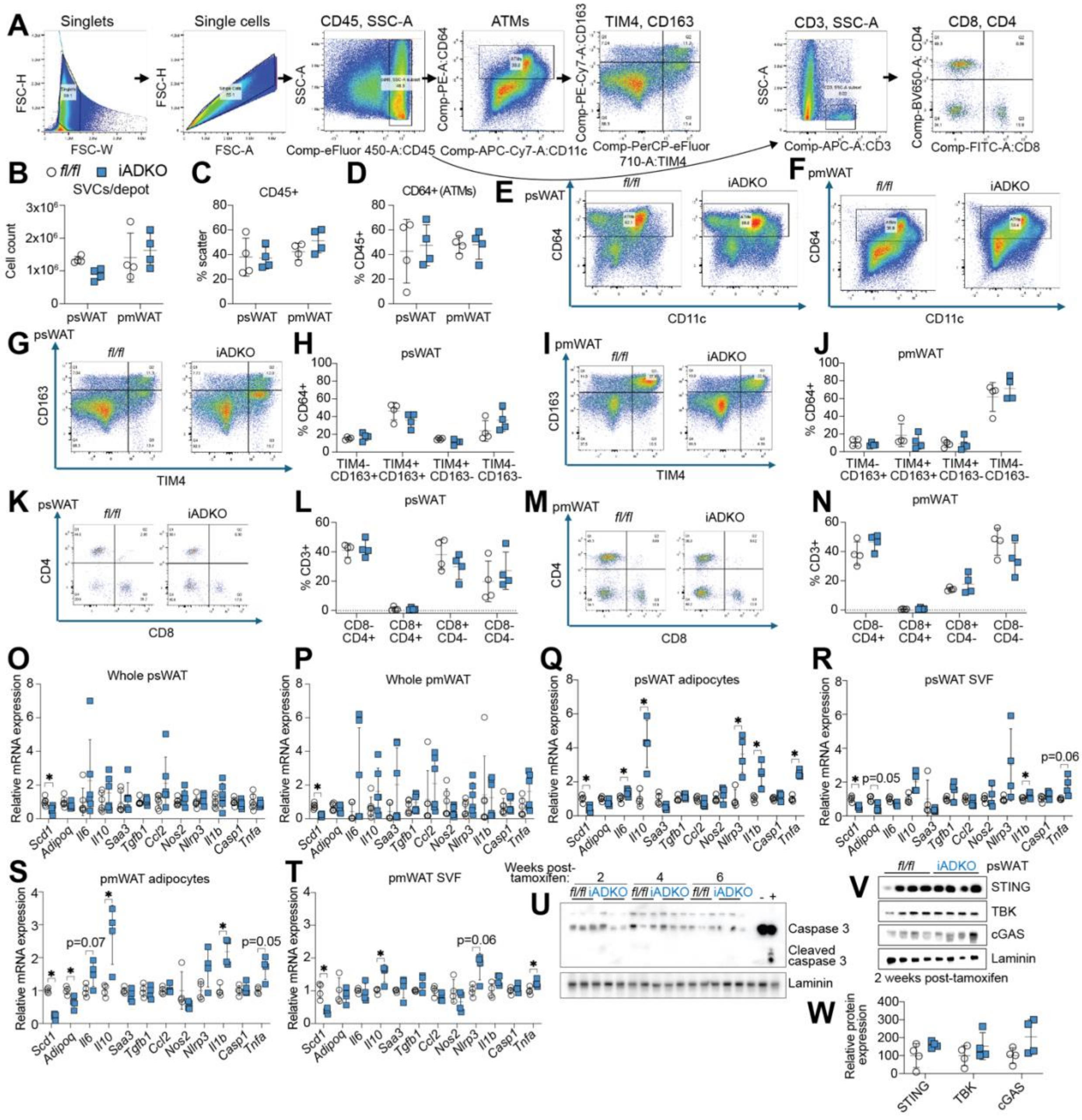

Supplemental Figure 10. Macrophages and T cells are not activated in *Lmna*iADKO WAT, and *Lmna* KO adipocytes have upregulated expression of pro-inflammatory transcripts. (**A**) Spectral flow cytometry gating strategy to identify macrophage and T cell populations from psWAT and pmWAT two-weeks post-tamoxifen (n = 4). (**B**) SVC number per depot. (**C**) CD45+ cells percentage. Representative density plot from CD45+ cells for CD64+ and CD11c+ adipose tissue macrophages (ATMs) from (**D**) psWAT and (**E**) pmWAT, with (**F**) quantification of CD64+

ATMs. Density plot from CD64+ cells for CD163+ and TIM4+ cells from (**G**) psWAT with (**H**) quantification, and from (**I**) pmWAT with (**J**) quantification. Density plot from CD3+ T cells that are CD4+ and CD8+ cells from (**K**) psWAT with (**L**) quantification, and from (**M**) pmWAT with (**N**) quantification. RT-qPCR analyses for inflammatory transcripts from (**O**) psWAT, (**P**) pmWAT, (**Q**) psWAT adipocytes, (**R**) psWAT SVF, (**S**) pmWAT adipocytes, and (**T**) pmWAT SVF (n = 4). (**U**) Immunoblot for cleaved and total caspase 3 in pmWAT post-tamoxifen (n = 3). Loading control = laminin. (**V**) Immunoblot for cGAS-STING pathway markers and (**W**) quantification from psWAT (n = 4). Loading control = laminin.

Supplemental Table 1. Most significant leading-edge genes from GSEA pathways associated with lipid metabolism, mitochondrial function, or inflammation; related to Figure 4 and Supplemental Figure 3.

Cell type	Disease state	GSEA pathway	Leading edge genes
**Adipocyte**	Developing vs. control	IL6/JAK/STAT3 Signaling	*PDGFC, CD36, CSF2RA, IFNGR2, STAT3, TNFRSF21, STAT2, FAS, CD38, IL1R1*
		Fatty Acid Metabolism	*ME1, G0S2, FASN, ADIPOR2, ELOVL5, PPARA, ACSL1, MGLL, GPD2, EPHX1*
	FPLD2 vs. control	IL6/JAK/STAT3 Signaling	*CD36, PDGFC, CD44, MAP3K8, IL1R1, STAT3, LEPR, IL4R, IL6ST, OSMR*
		Fatty Acid Metabolism	*FASN, G0S2, ME1, PPARA, IDH1, ADIPOR2, MGLL, DECR1, ELOVL5, GLUL*
		Aerobic Respiration	*MT-CO2, MT-ATP6, NT-ND3, MT-CYB, MT-CO3, MT-CO1, MT-ND4, MT-ND2, MT-ND1*
**ASPC**	Developing vs. control	Lipid Transporter Activity	*ABCA6, ABCA9, ABCA10, ABCA8, ABCA1, BLTP1, ABCB11, PRELID2, APOL3, SLCO3A1*
		Oxidative Phosphorylation	*GPX4, ATP1B1, COX4I1, LDHA, ATP5F1E, SLC25A6, COX5B, COX6A1, MGST3, COX7C*
	FPLD2 vs. control	Lipid Transporter Activity	*ABCA10, ABCA8, ABCA9, ABCA6, ABCA1, SLCO3A1, ABCC1, PRELID2, VMP1, ATP8B4*
		Oxidative Phosphorylation	*ATP5F1E, COX4I1, ATP1B1, SLC25A6, GPX4, COX7C, COX6A1, ATP5MC3, UQCRB, COX6B1*
**Macrophage**	Developing vs. control	Lipid Storage Regulation	*MSR1, PPARG, LPL, TTC39B, ACACB, PLIN2, CPT1A, C3, NR1HE, SREBF2*
		Interferon Alpha Response	*B2M, CD74, HLA-C, IFI30, IFITM3, BST2, TXNIP, IFITM2, PSME1, LPAR6*
		Oxidative Phosphorylation	*ATP5F1E, ATP6V1F, COX4I1, SLC25A6, COX6B1, COX8A, UQCRB, SLC25A5, COX7C, ATP6V0C*
	FPLD2 vs. control	Lipid Storage Regulation	*PPARG, NR1H3, TTC39B, SCARB1, SPT1A, MSR1, ACACB, SREBF2, C3, PLIN2*
		Interferon Alpha Response	*B2M, CD74, IFI30, HLA-C, TXNIP, IFITM3, BST2, IFITM2, PSME1 (only 9 in list)*
		Oxidative Phosphorylation	*ATP5F1E, COX4I1, ATP6V1F, COX8A, SLC25A6, ATP6V0C, UQCRB, COX7C, SLC25A5, COX6B1*
**Endothelial**	Developing vs. control	Adipogenesis	*PPARG, CD36, GBE1, FABP4, SORBS1, ITIH5, COL4A1, LPL, LAMA4, CYP4B1*
	FPLD2 vs. control	Interferon Alpha Response	*B2M, IFITM3, HLA-C, CD74, BST2, IFI27, IFITM2, IFITM1, LPAR6, LY6E*
		Adipogenesis	*FABP4, PPARG, UBC, SORBS1, LPL, CD36, LIPE, CAVIN2, ITIH5, COX6A1*
		Oxidative Phosphorylation	*ATP5F1E, COX7C, COX4I1, COX5B, COX6A1, COX6B1, NDUFA1, COX7A2, SLC25A6, ATP5PF*
**T Cell**	Developing vs. control	Adipogenesis	*APOE, LPL, PFKFB3, SORBS1, PTGER3, PLIN2, ITIH5, ADCY6, PHLDB1, FABP4*
		Interferon Alpha Response	*B2M, LPAR6, SELL, IFITM2, SAMD9, CD47, GBP22, SP110, PSMB9, TRIM5*
	FPLD2 vs. control	IL2/STAT5 Signaling	*AHNAK, CD44, ADAM19, CSF1, PHTF2, GLIPR2, TRAF1, PHLDA1, HYCC2, BHLHE40*
		Interferon Alpha Response	*B2M, SELL, IFITM2, LPAR6, PSME1, HLA-C, CD74, TXNIP, PSMA3, CD47*
		Oxidative Phosphorylation	*COX7C, SLC25A6, COX4I1, COX5B, COX6B1, FOX6A1, NDUFA1, COX7B, UQCRB, TIMM9*

**Pericyte**	Developing vs. control	Adipogenesis	*COL15A1, SPARCL1, MGST3, CD151, FABP4, PPARG, ITSN1, COX8A, UBC, NDUFB7*
		Oxidative Phosphorylation	*MGST3, ATP5F1E, COX8A, NDUFB7, UQCR11, SLC25A11, NDUFB2, NDUFA4, LDHB, IDH3B*
	FPLD2 vs. control	Adipogenesis	*SPARCL1, FABP4, UBC, CD151, COL15A1, COX6A1, CHCHD10, APOE, CAVIN1, SNCG*
		Oxidative Phosphorylation	*SLC25A6, ATP5F1E, COX6A1, COXIRI1, NDUFA4, COX5B, COX7C, ATP5MF, COX8A, NDUFB7*
**LEC**	Developing vs. control	Oxidative Phosphorylation	*POLR2F, NDUFS6, SDHD, SLC25A6, UQCRH, ALDH6A1, COX5B, COX8A, SLC25A5, HSD17B10*
	FPLD2 vs. control	Interferon Alpha Response	*B2M, IFITM3, TXNIP, IFITM2, HLA-C, PSME1, TRIM25, IFI30, PSME2, PROCR*
		Oxidative Phosphorylation	*SLC25A6, COX7C, UQCRB, COX6A1, COX5B, COX7A2, SLC25A5, COXIRI1, ISCU, ATP5MC2*
