# Supplementary material for: Altered lipid metabolism and inflammatory programs associate with adipocyte loss in familial partial lipodystrophy 2
Source: J Clin Invest. 2025 Nov 11;136(1):e198387. doi: 10.1172/JCI198387 (PMC12721891; doi:10.1172/JCI198387)

Raw unedited blot for Figure 7A, B
PPARg

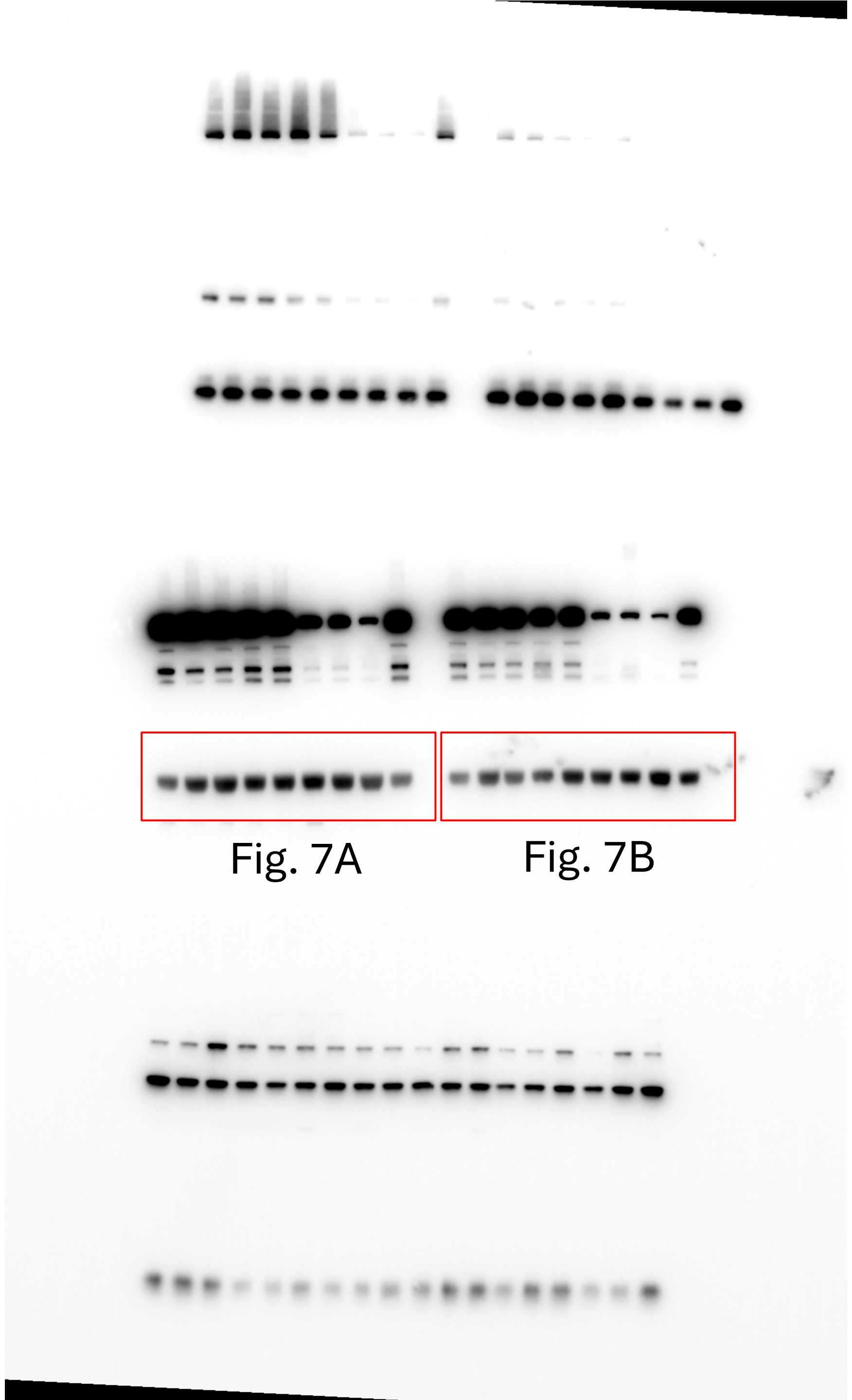

Raw unedited blot for Figure 7A, B
C/EBPa

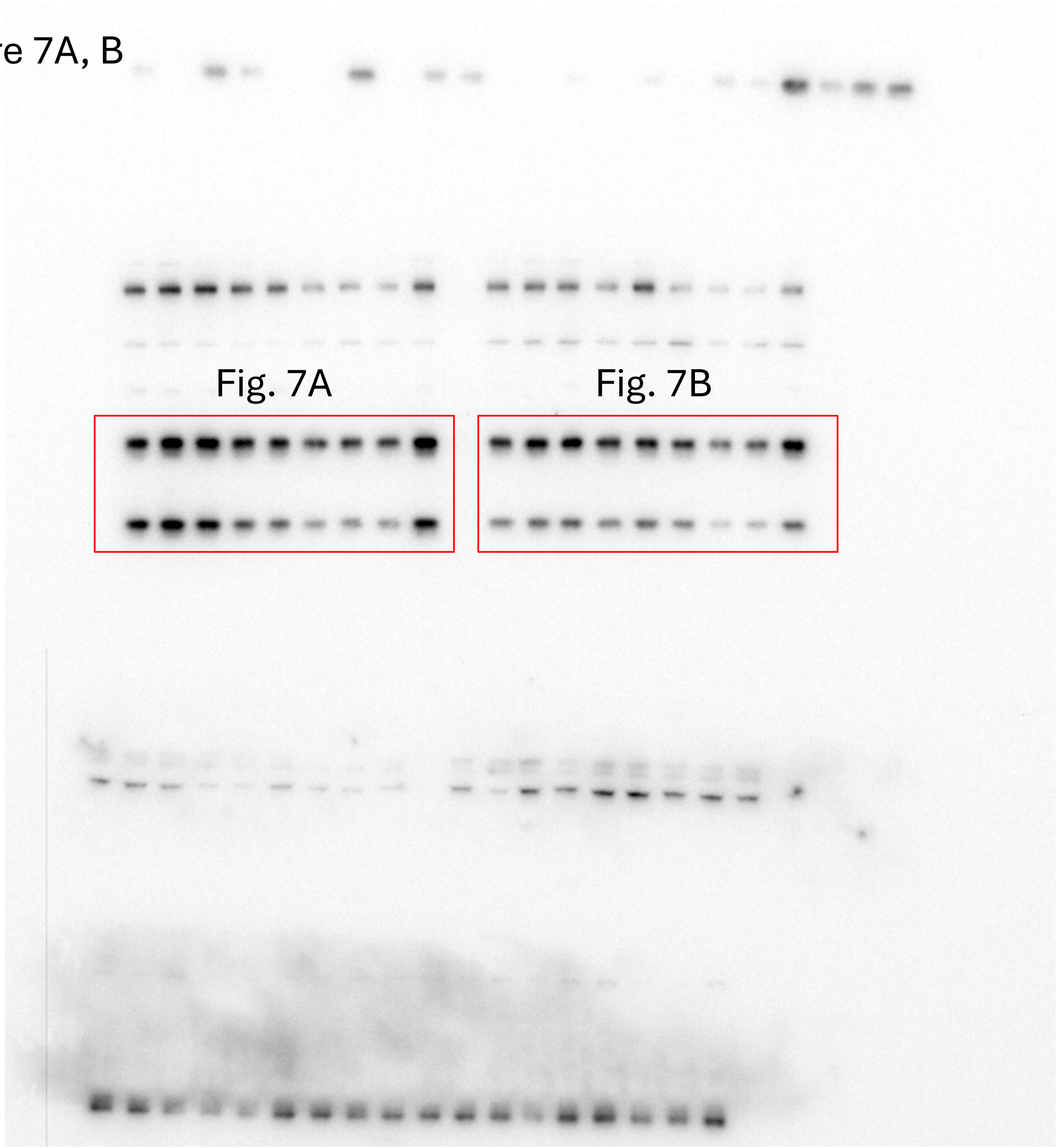

Raw unedited blot for Figure 7A, B
ChREBP

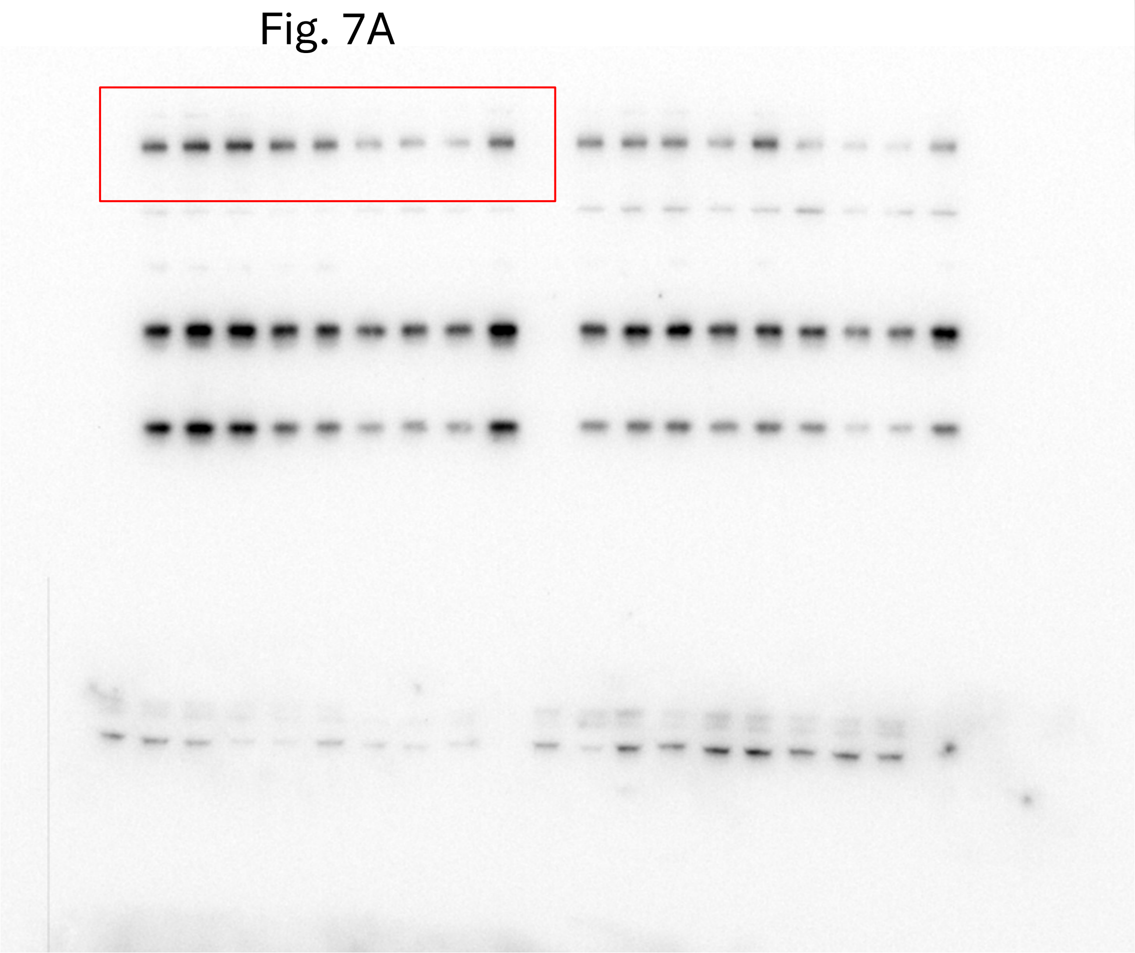

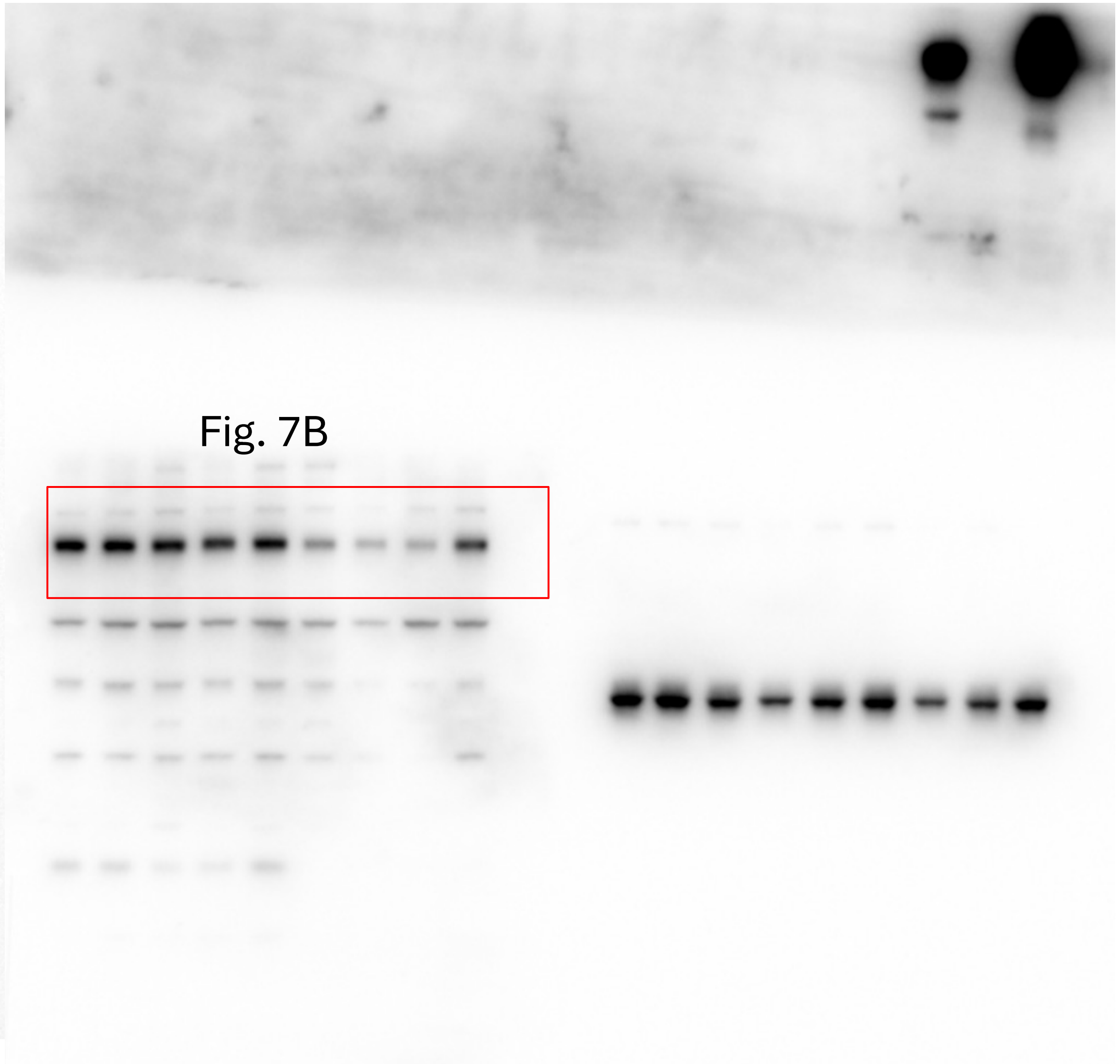

Raw unedited blot for Figure 7A, B
SREBP

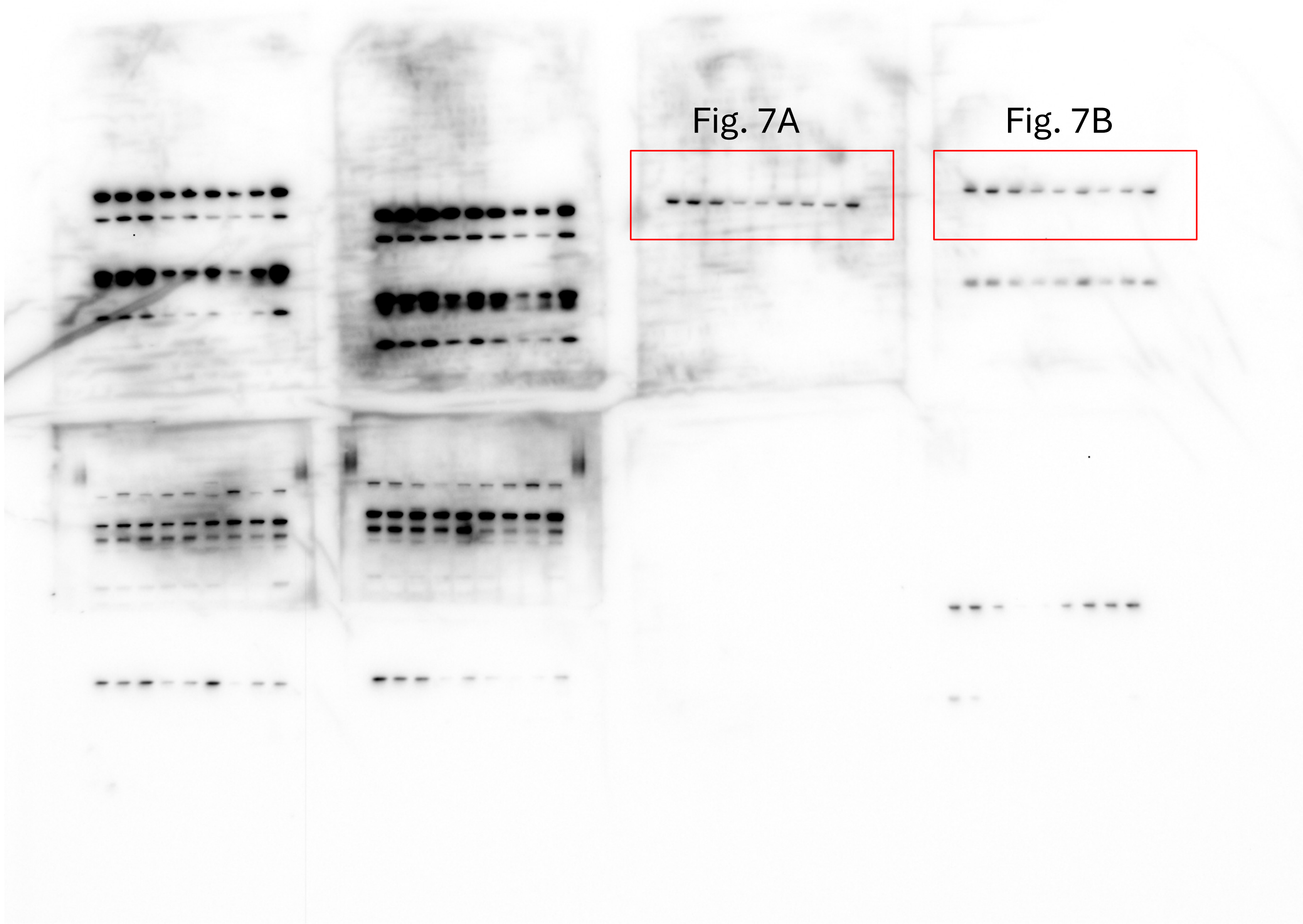

Raw unedited blot for Figure 7A, B
ACC

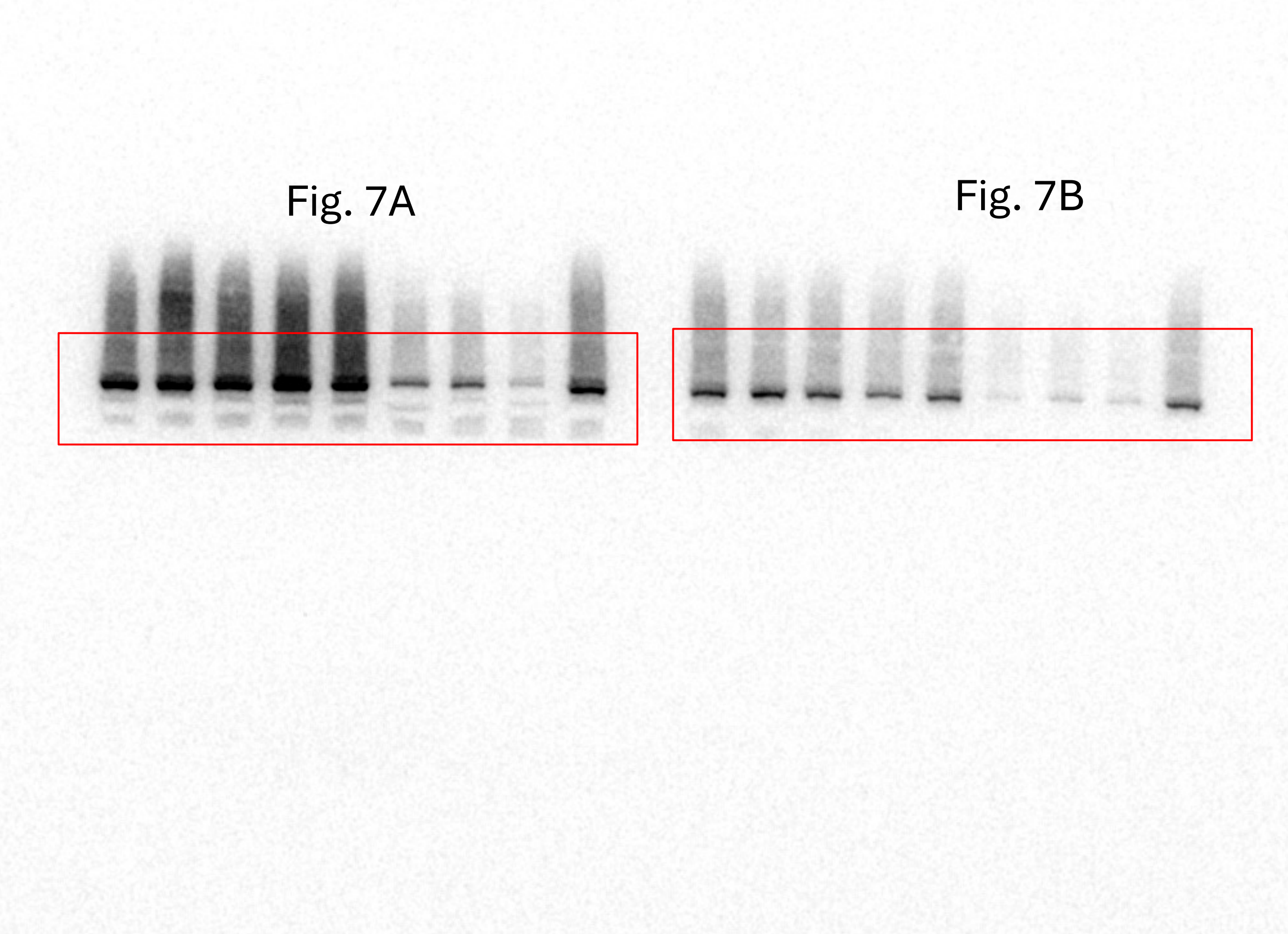

Raw unedited blot for Figure 7A, B
FASN

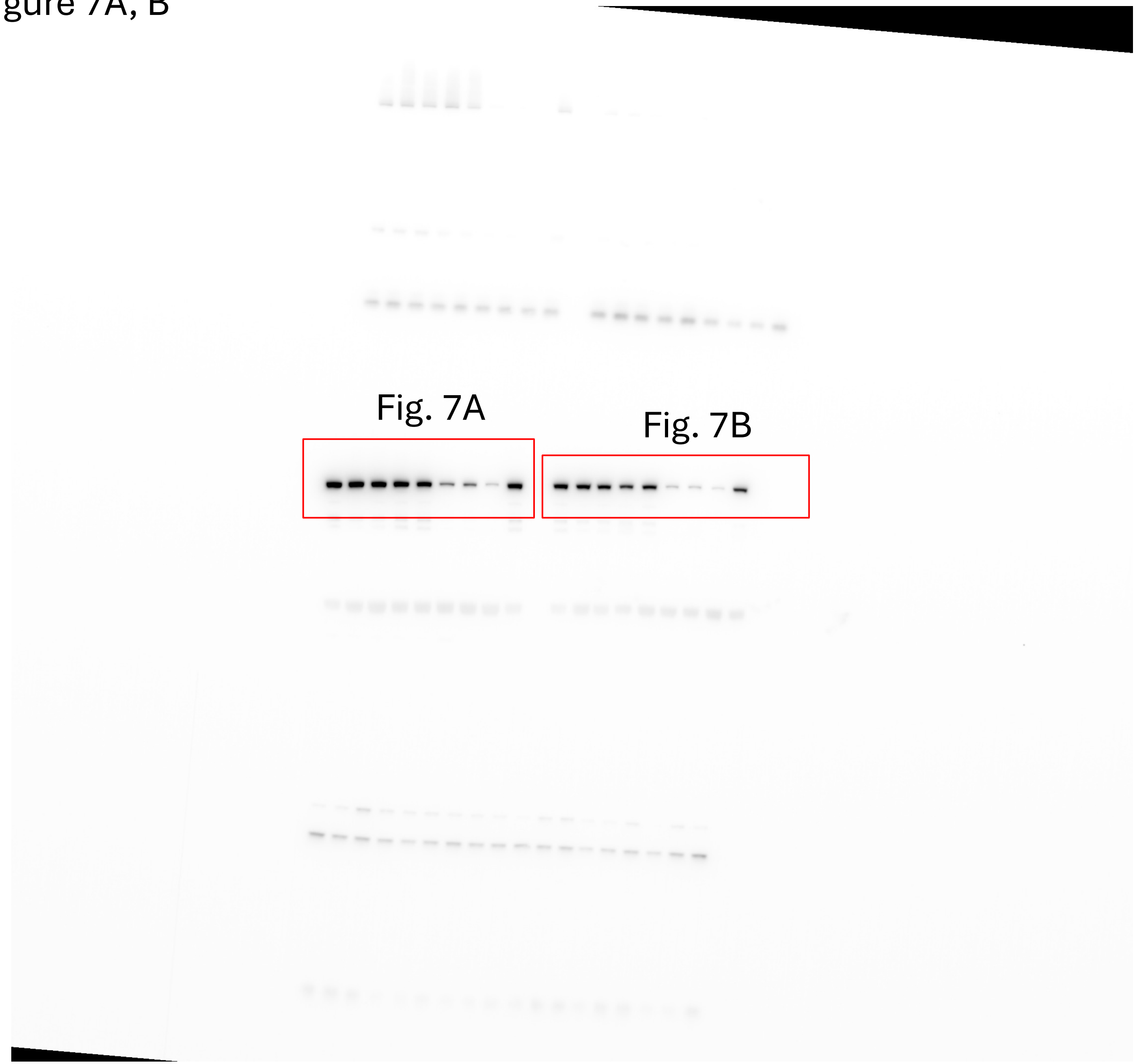

Raw unedited blot for Figure 7A, B
SCD1

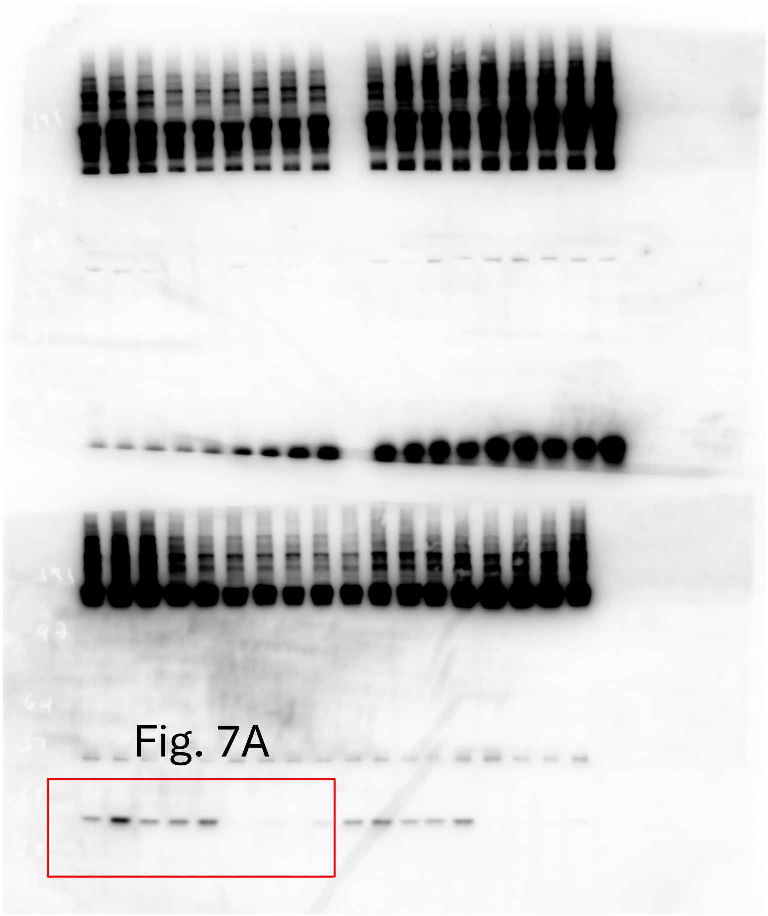

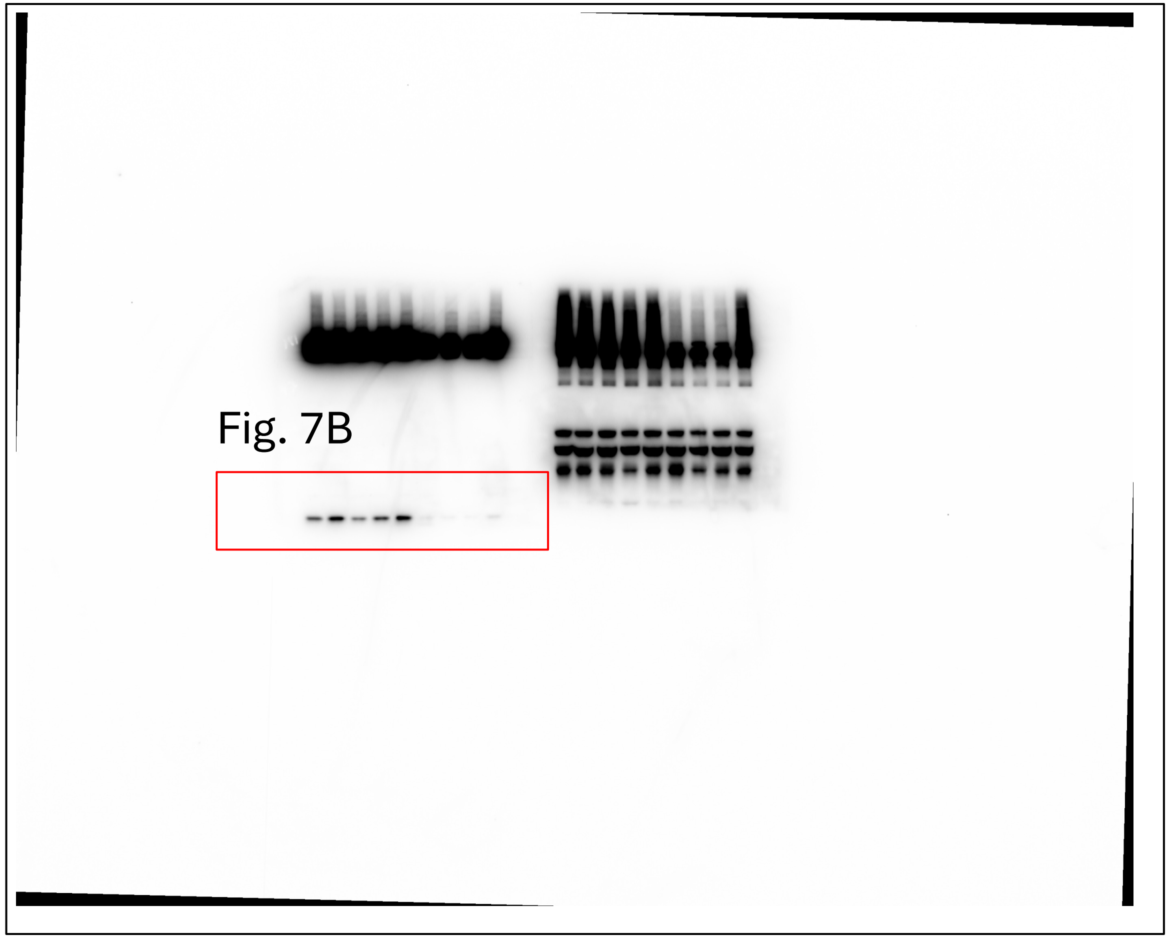

Raw unedited blot for Figure 7A, B
Adiponectin

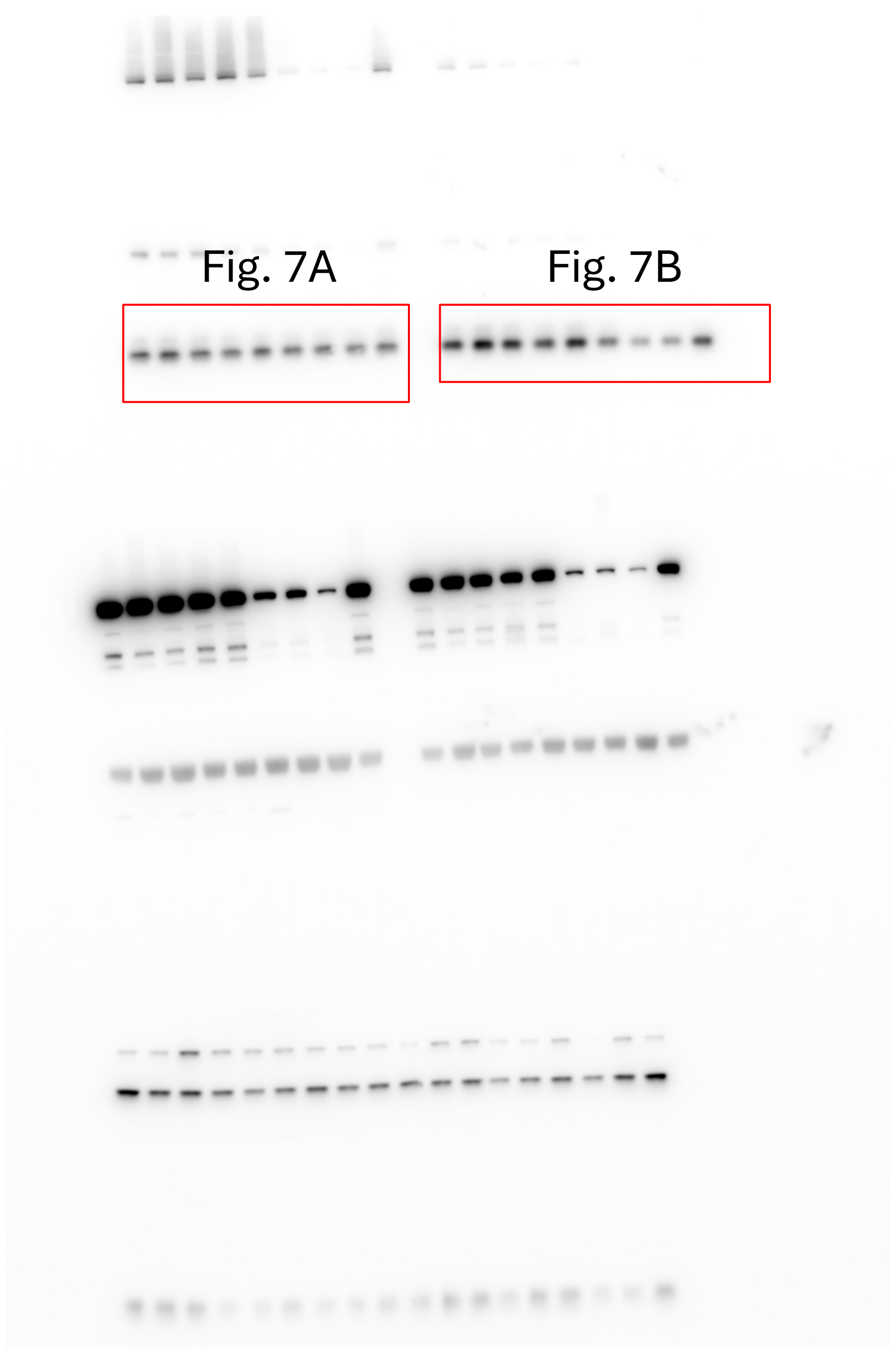

Raw unedited blot for Figure 7A, B
Laminin

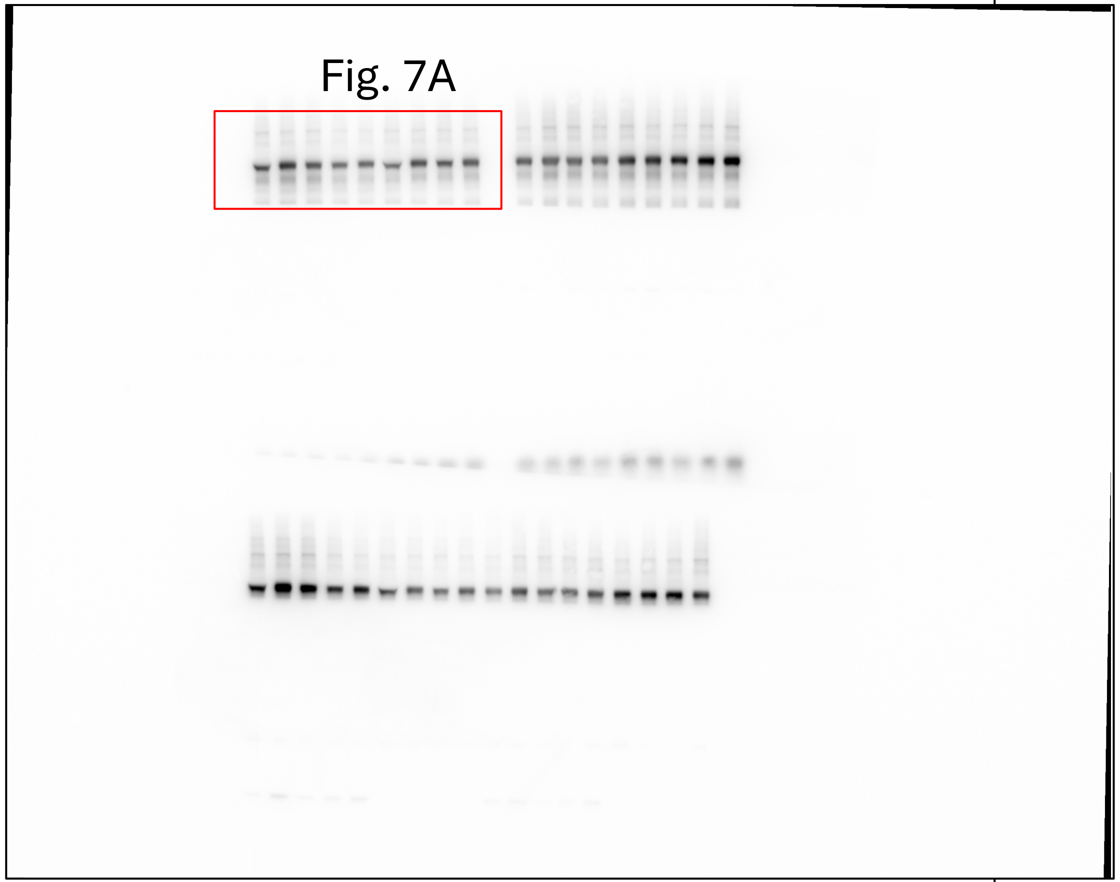

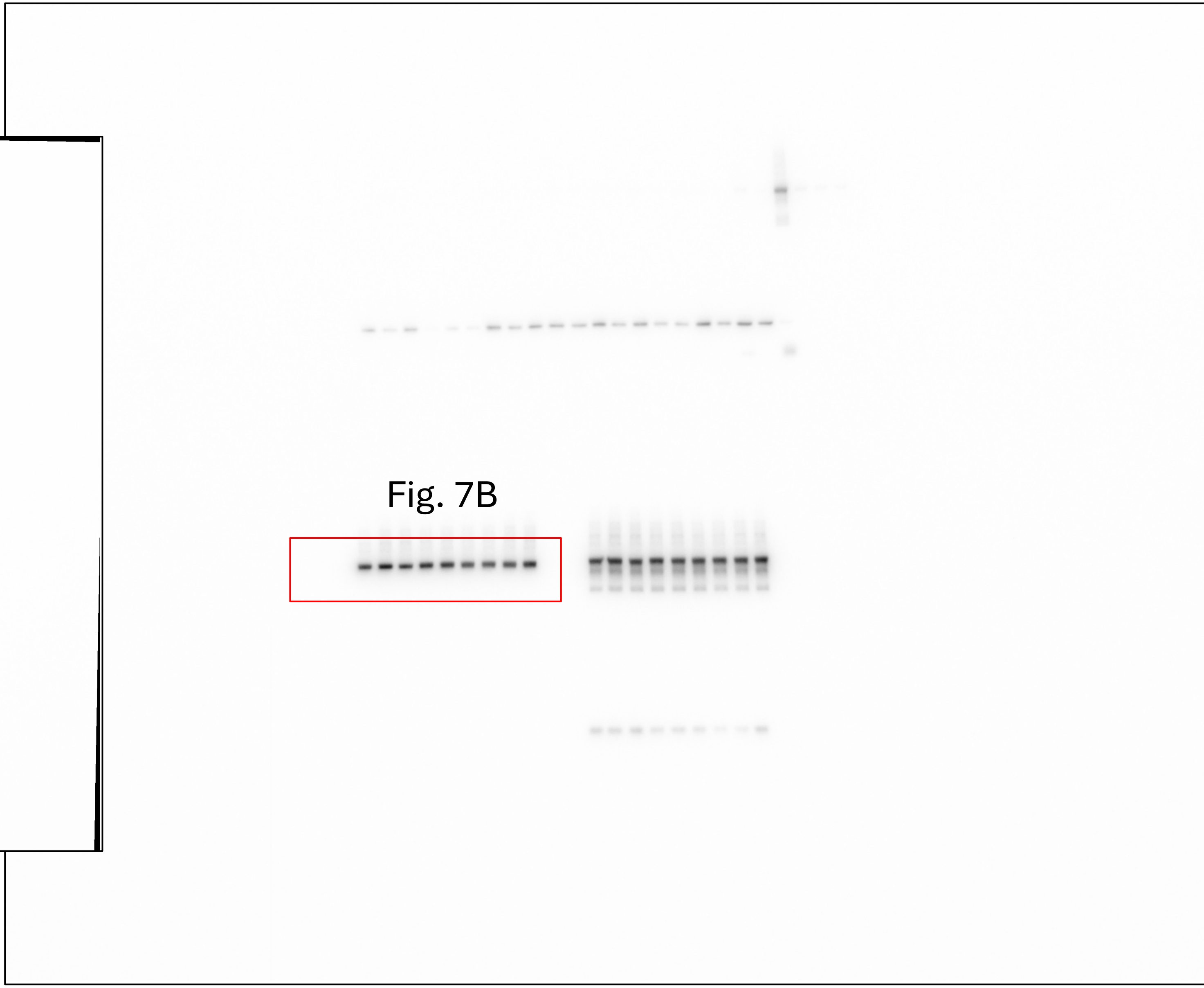

Raw unedited blot for Figure 7C
OXPHOS complexes

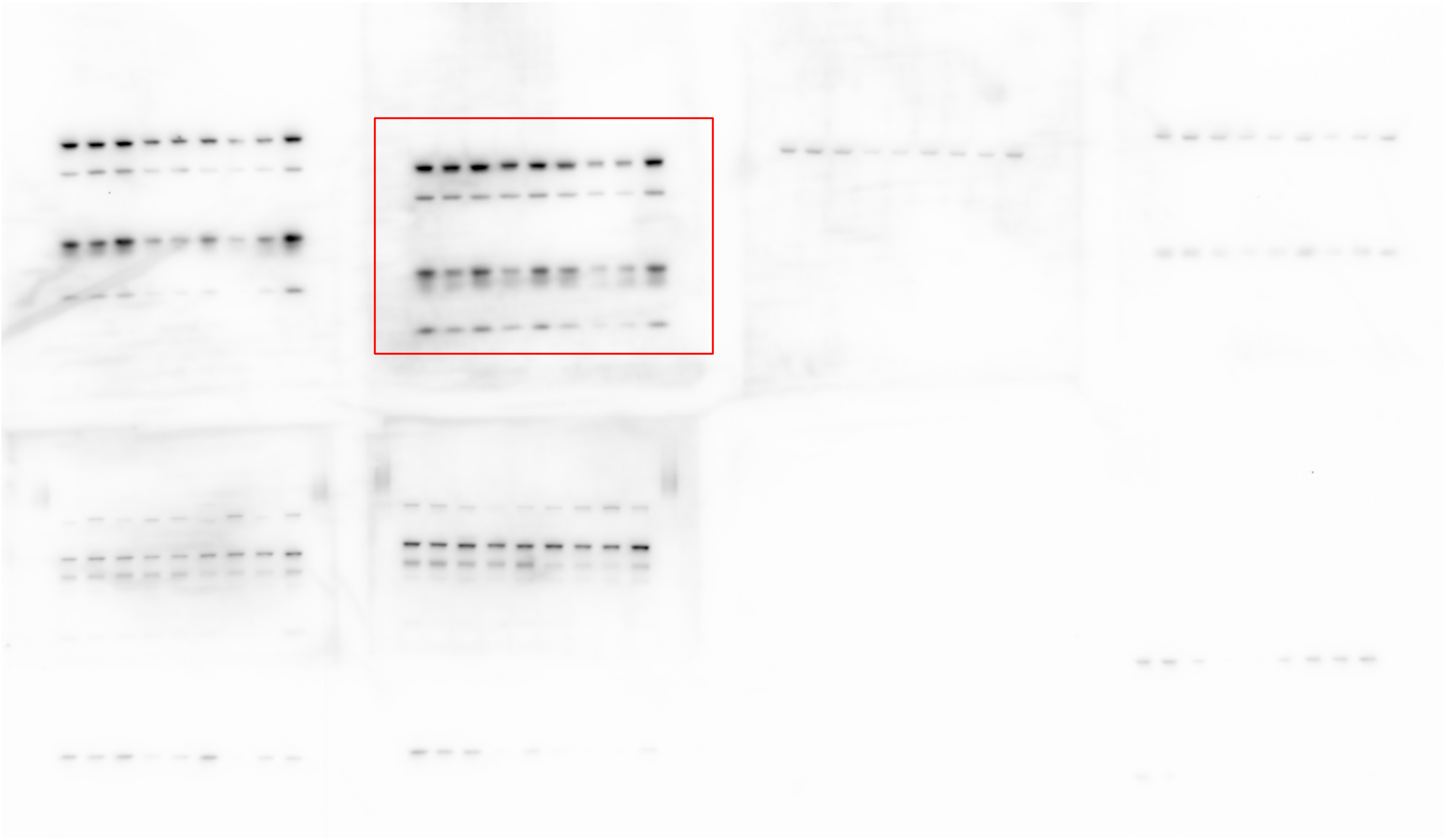

Raw unedited blot for Figure 7C
ACAA2

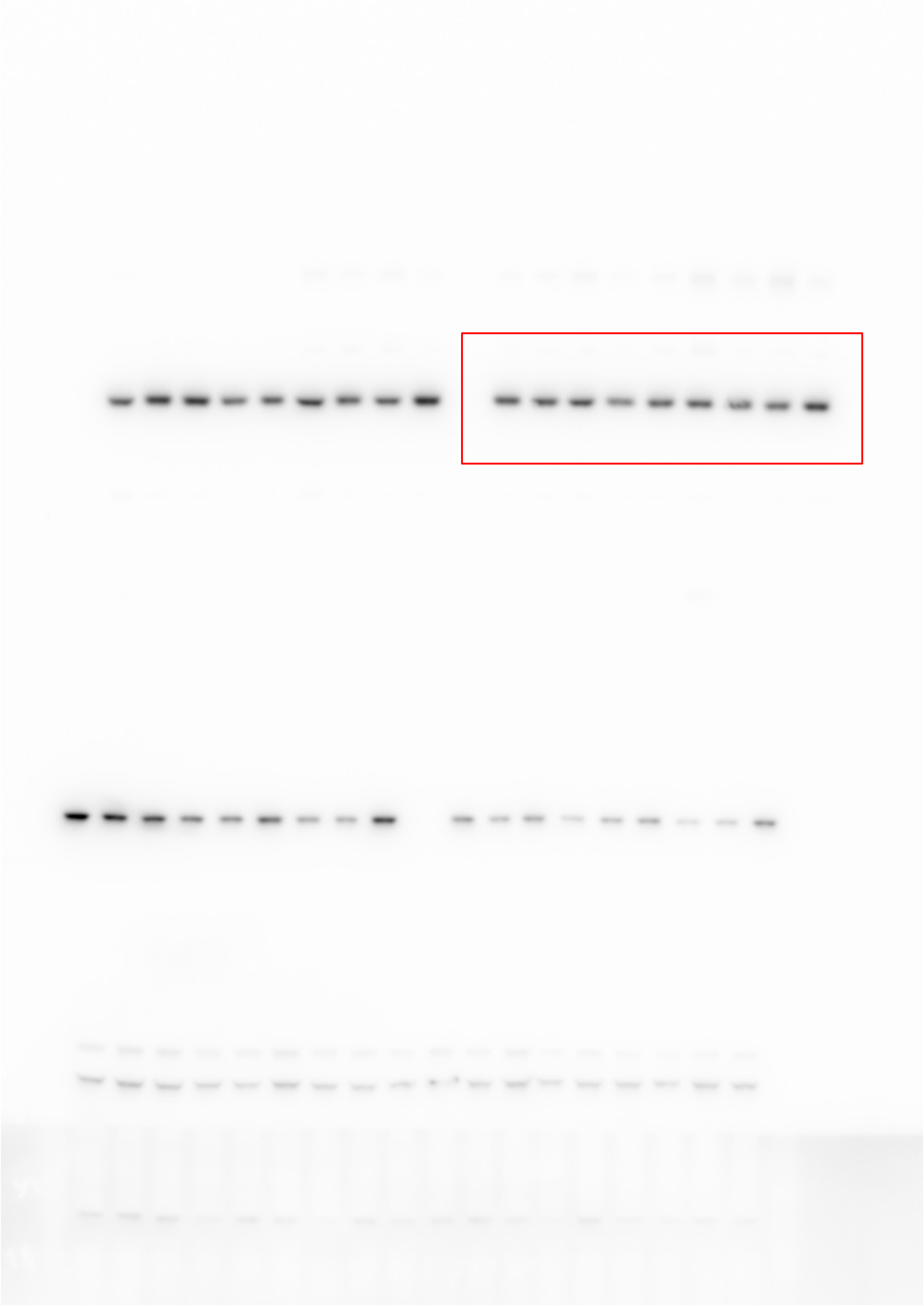

Raw unedited blot for Figure 7C
HADHB

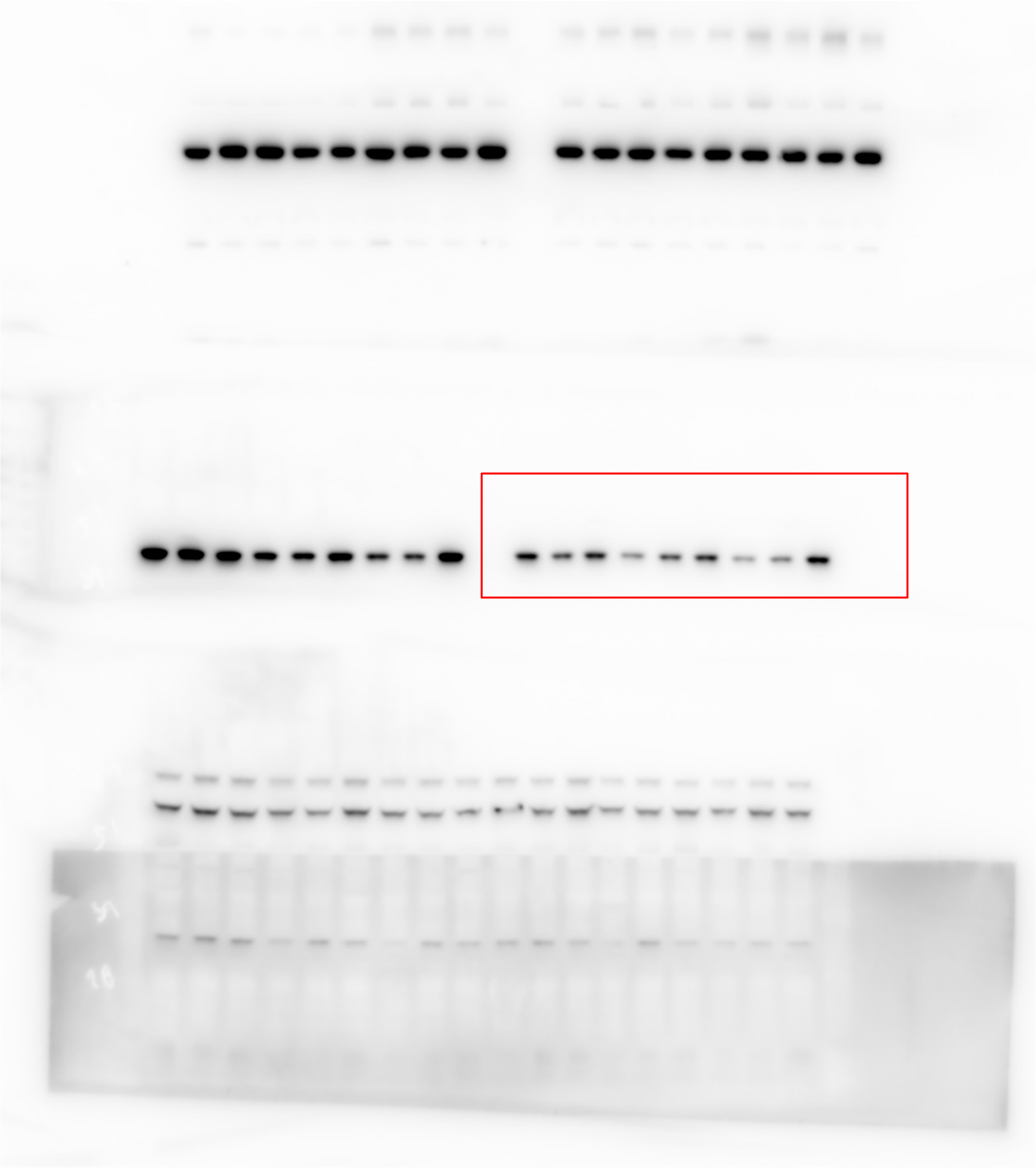

Raw unedited blot for Figure 7C
CPT1a

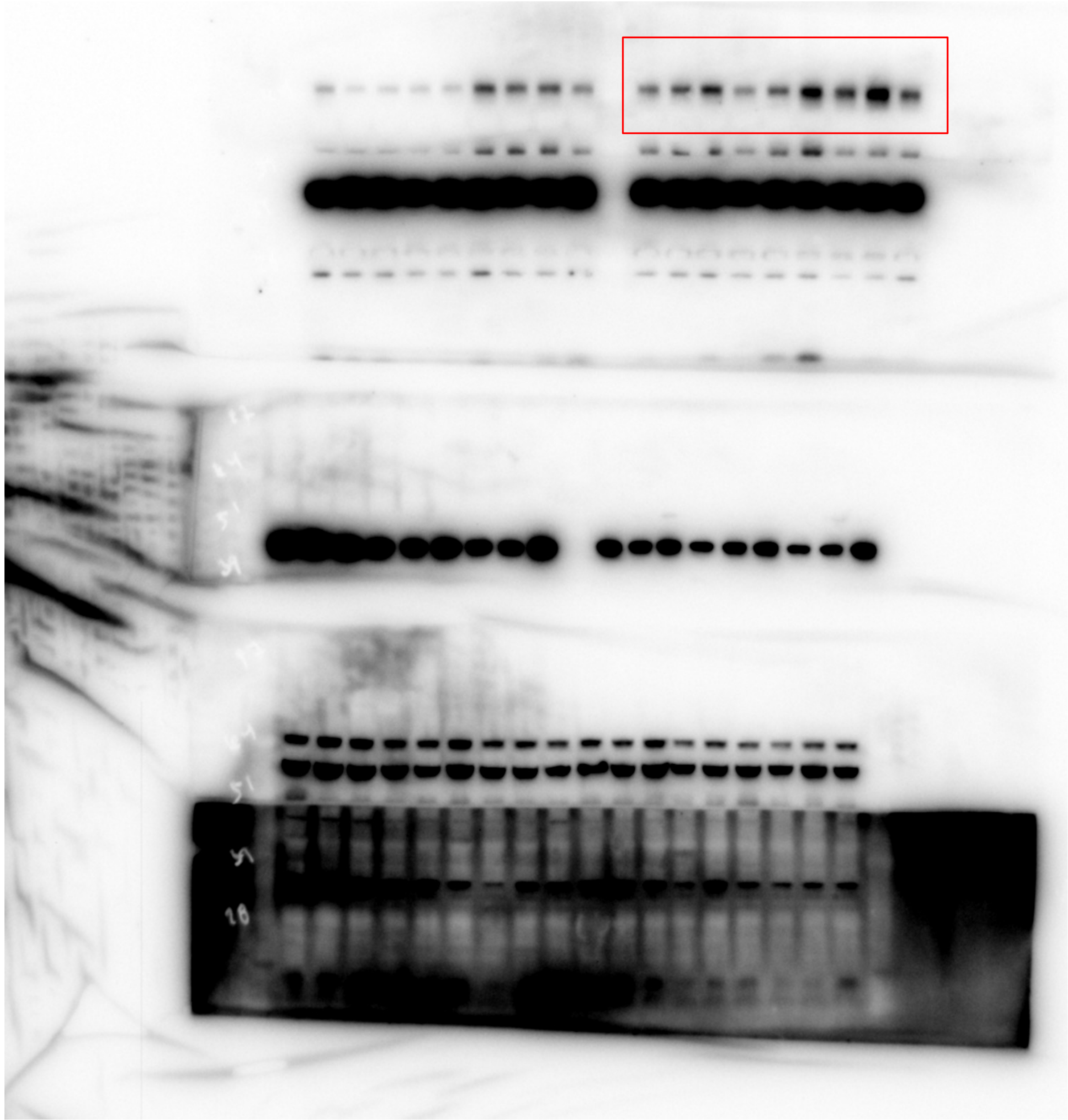

Raw unedited blot for Figure 7C
Pyruvate dehydrogense

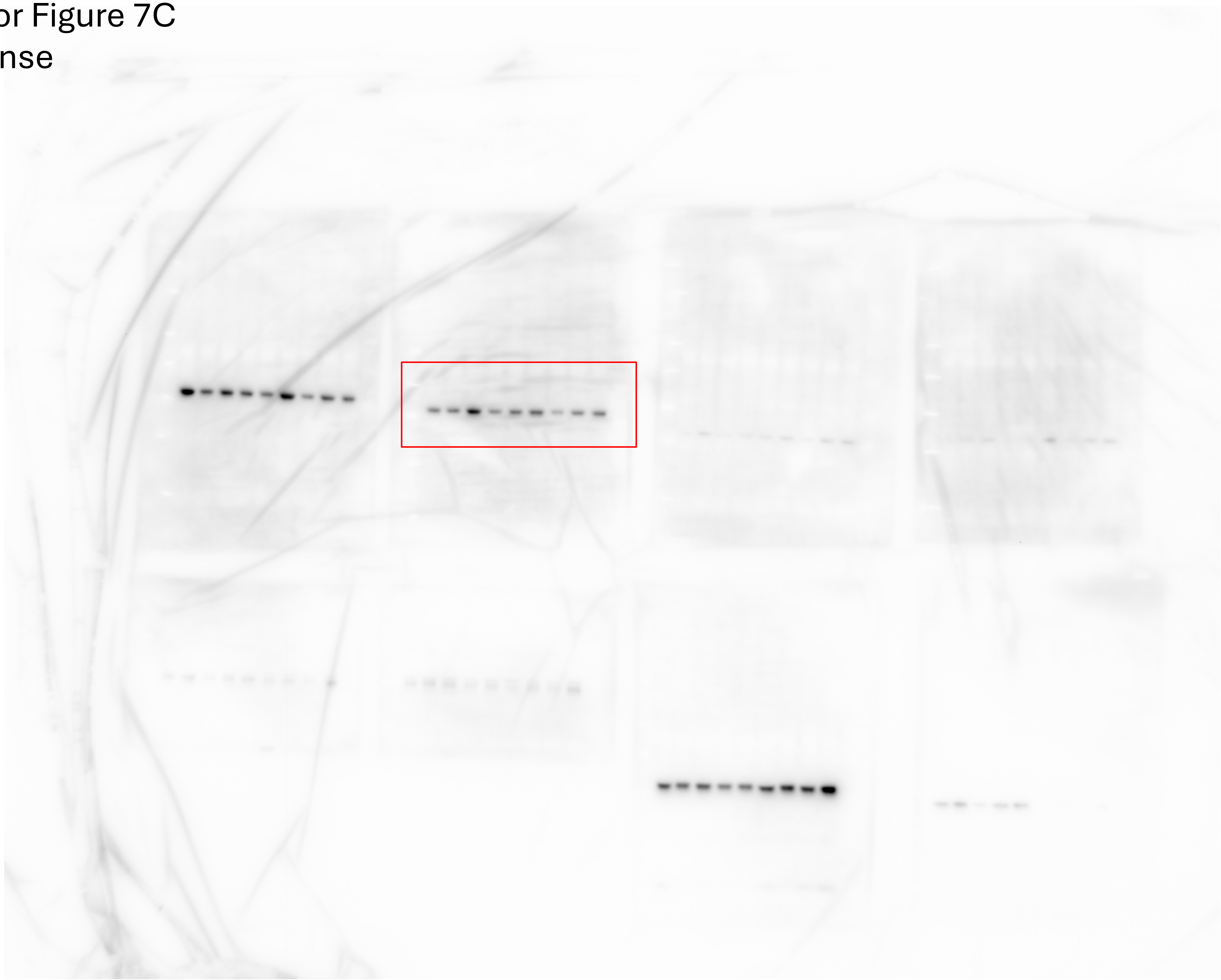

Raw unedited blot for Figure 7C
Laminin

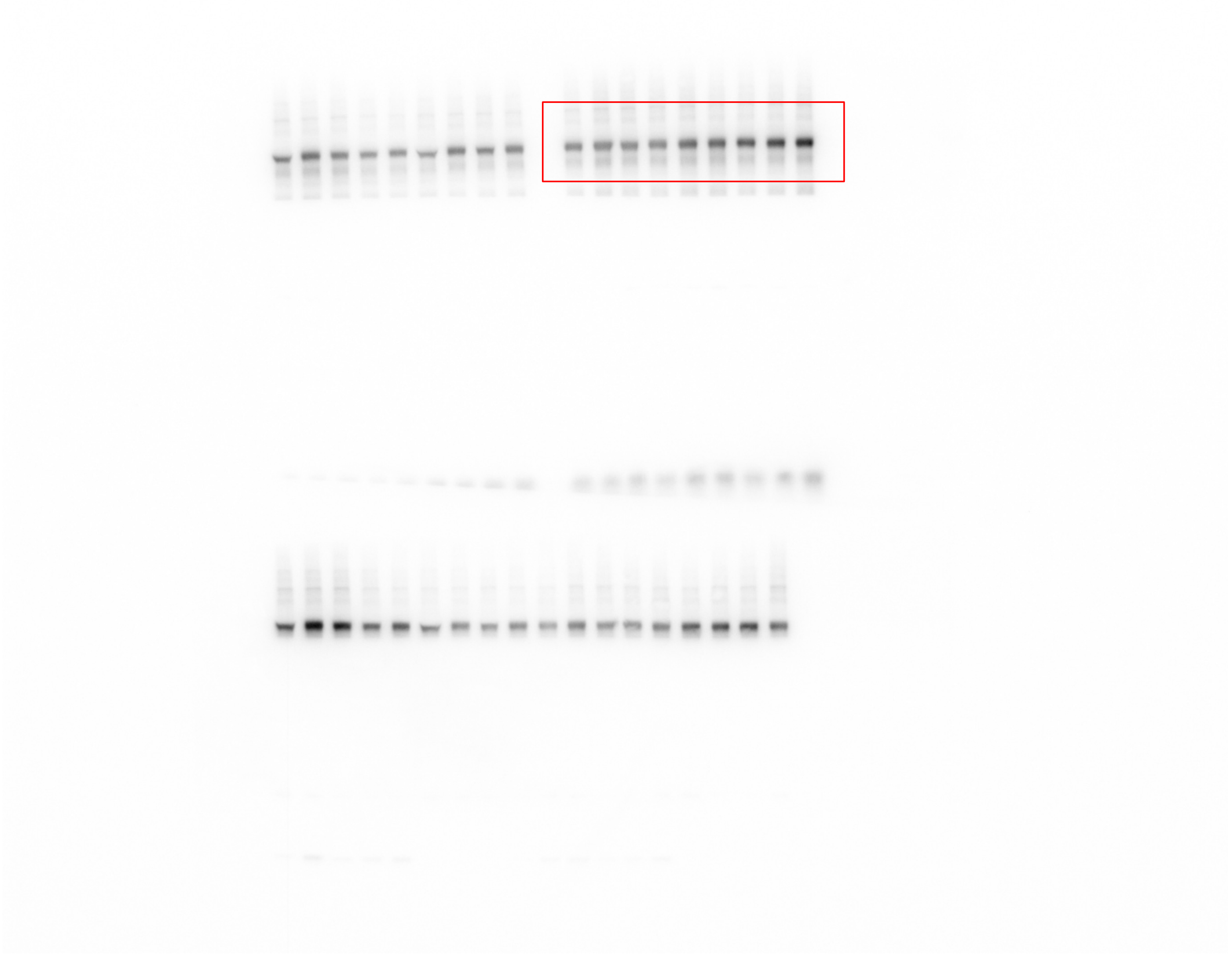

Raw unedited blot for Figure 7F
OXPHOS complexes

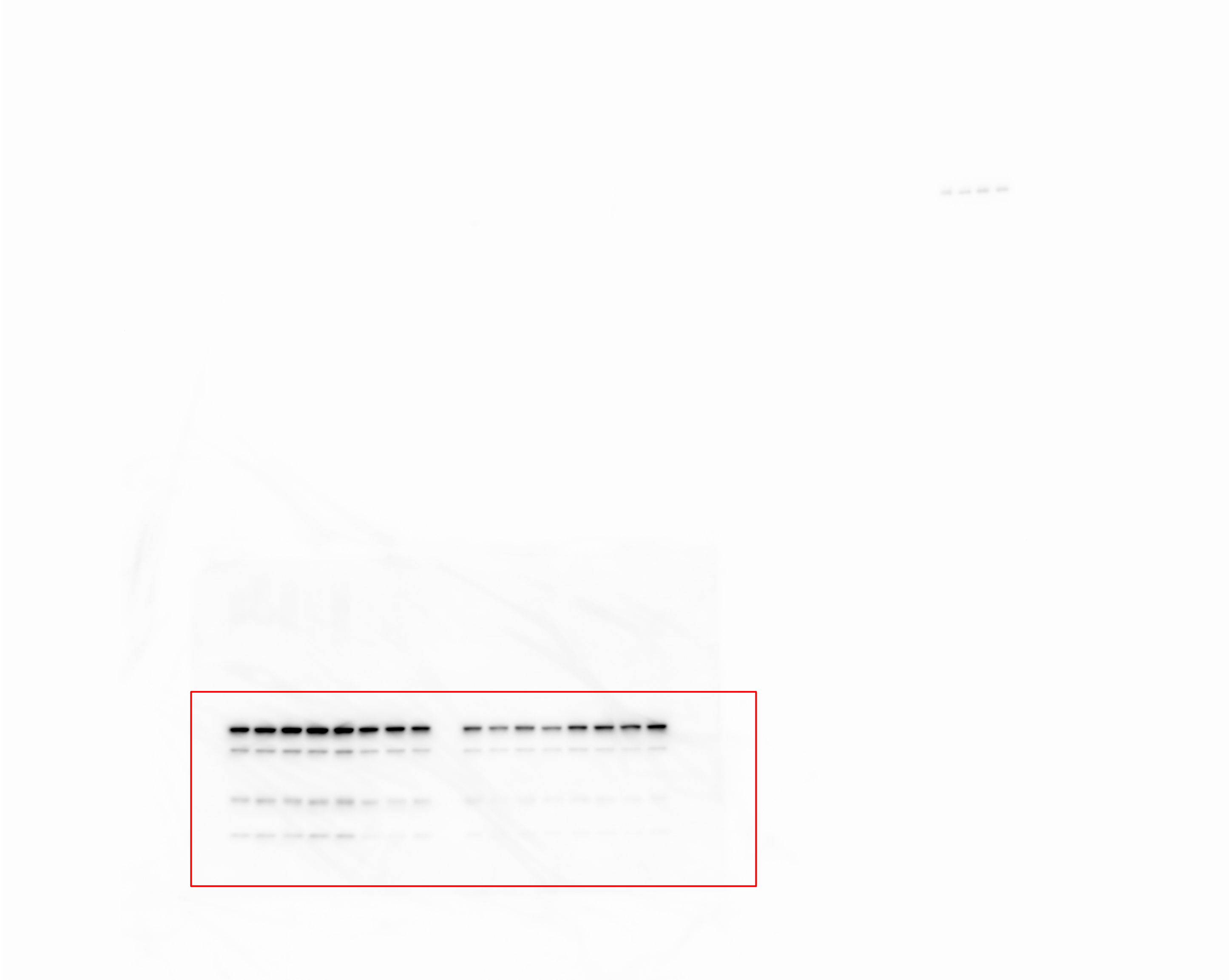

Raw unedited blot for Figure 7F
PGC1a

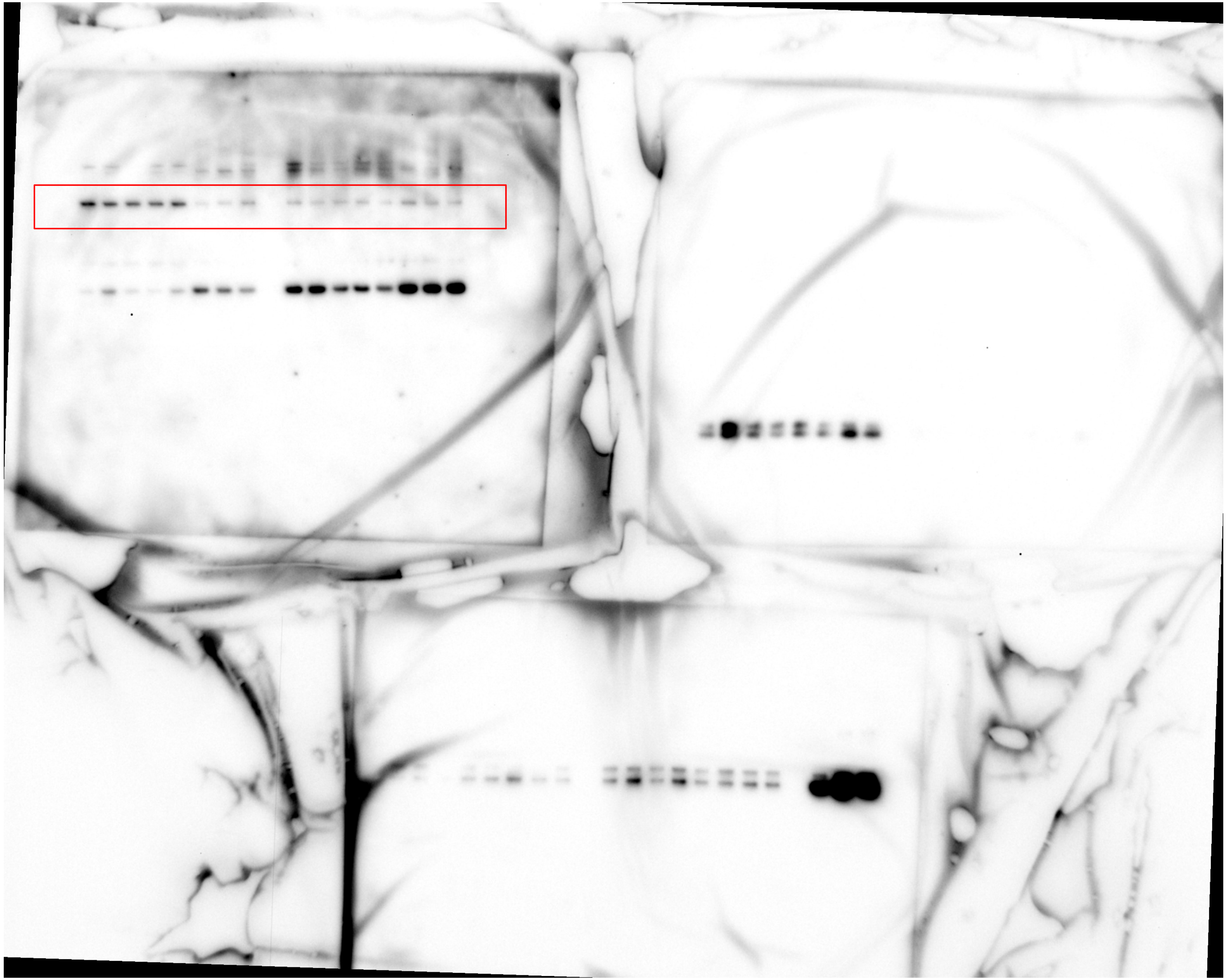

Raw unedited blot for Figure 7F
MFN2

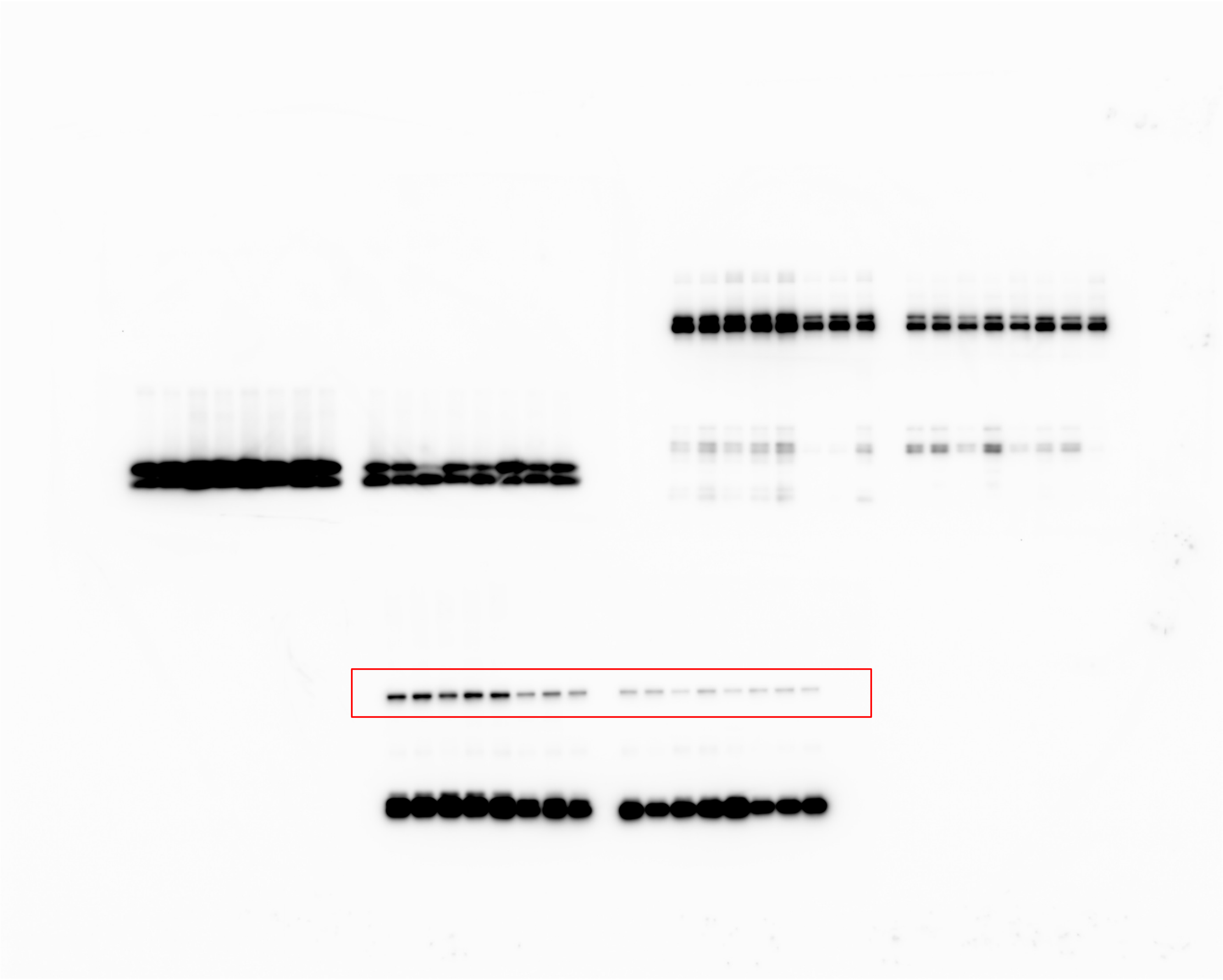

Raw unedited blot for Figure 7F
OPA1

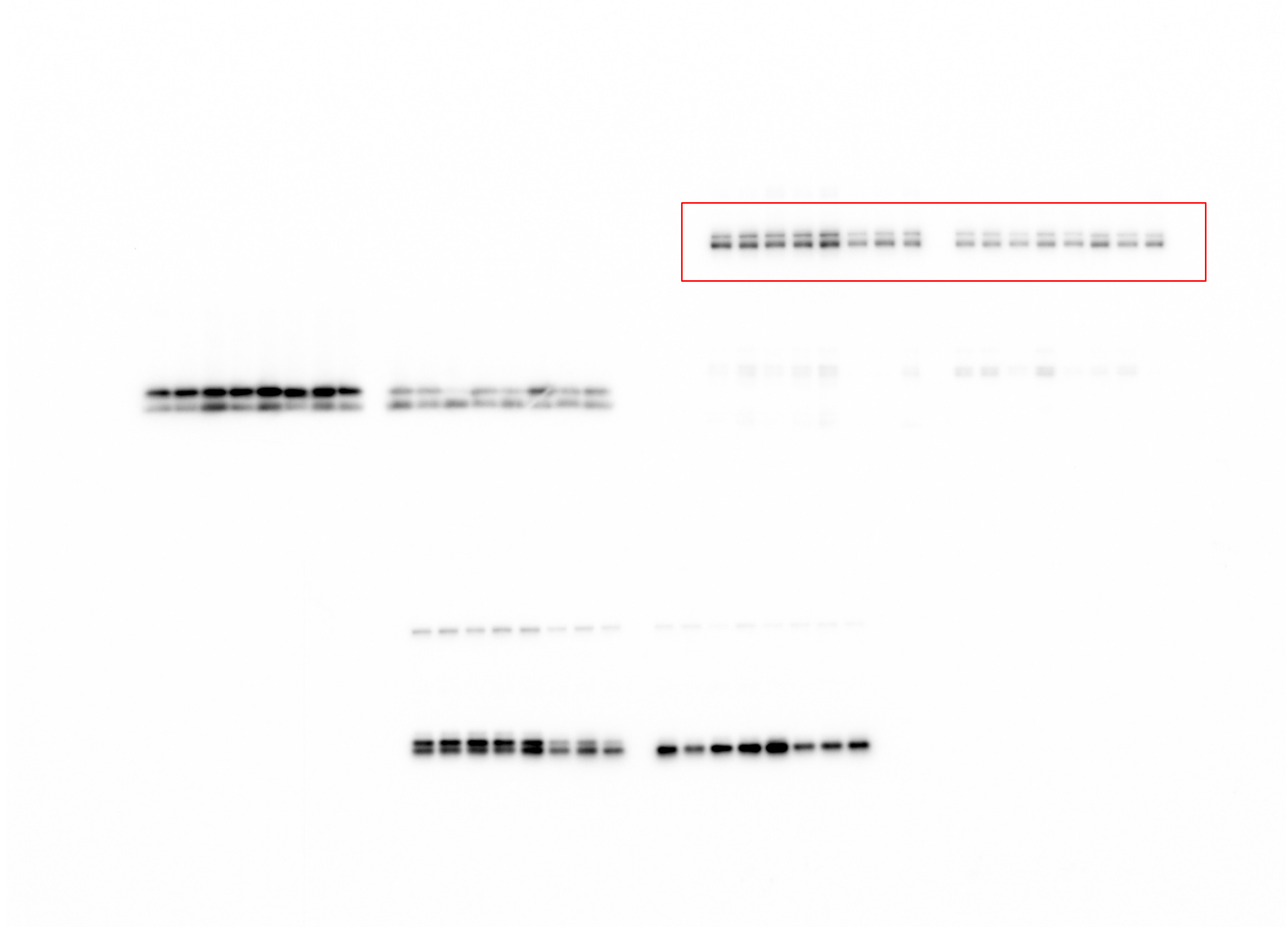

Raw unedited blot for Figure 7F
MFF

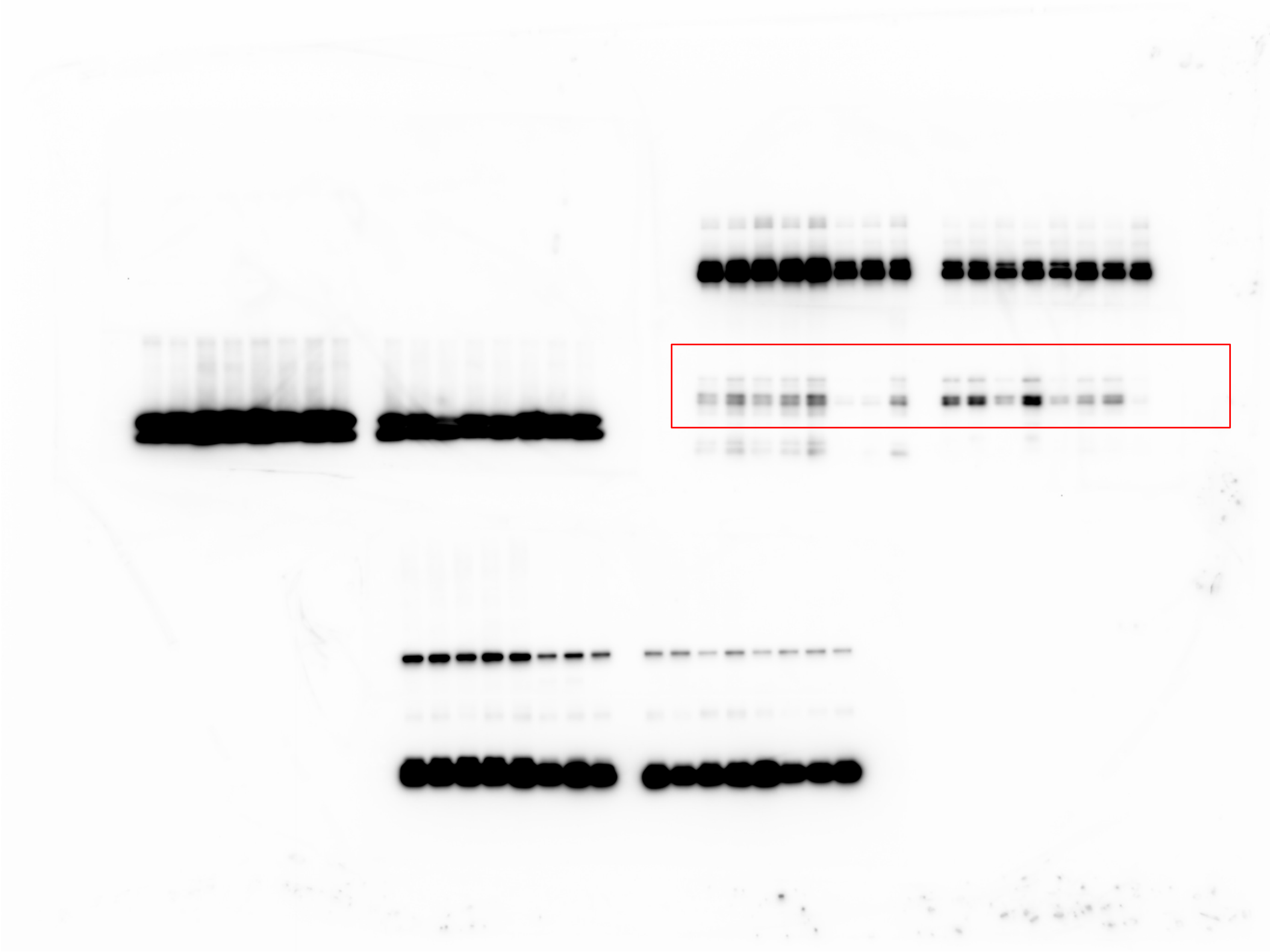

Raw unedited blot for Figure 7F
TOM20

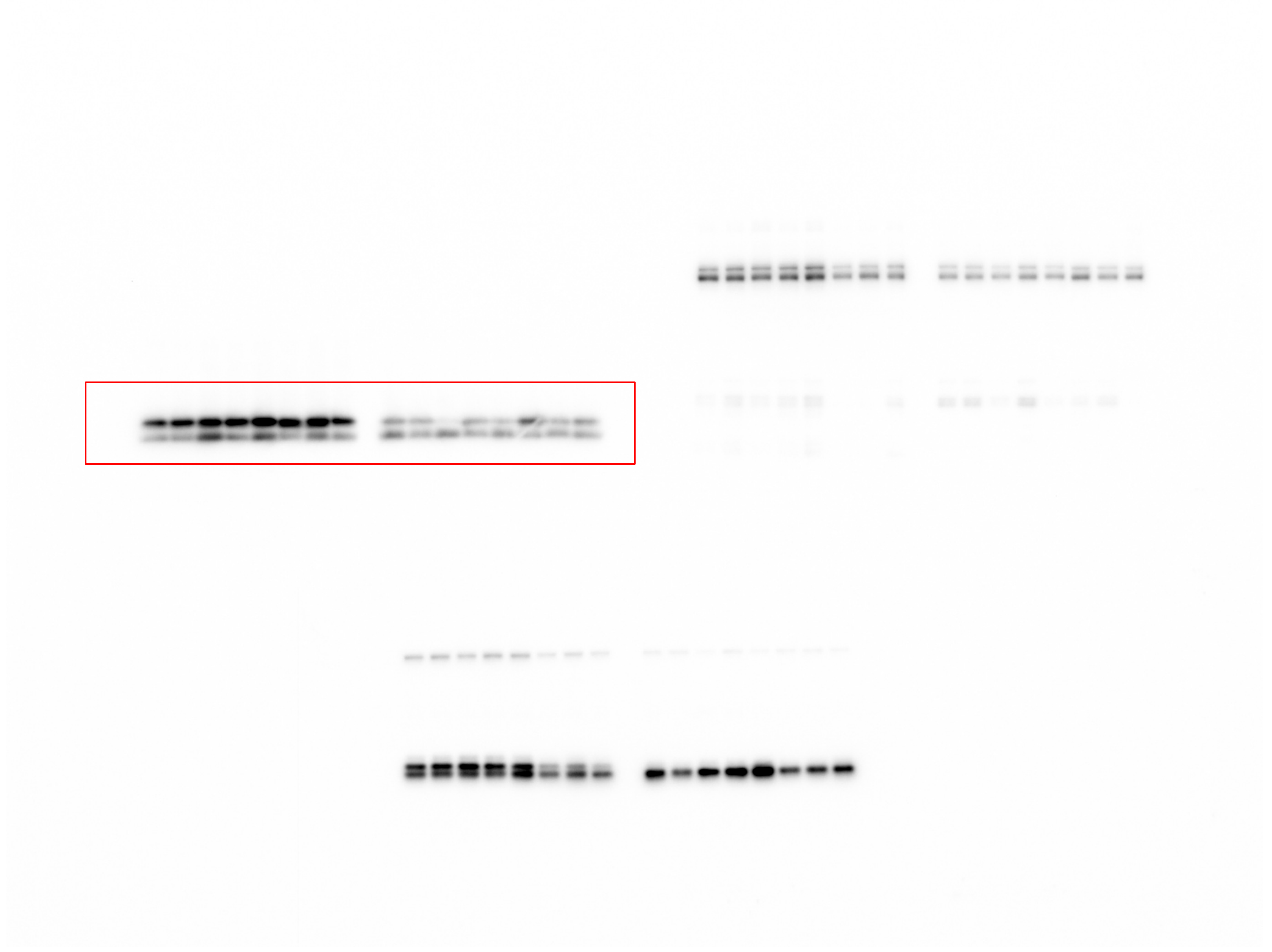

Raw unedited blot for Figure 7F
DRP

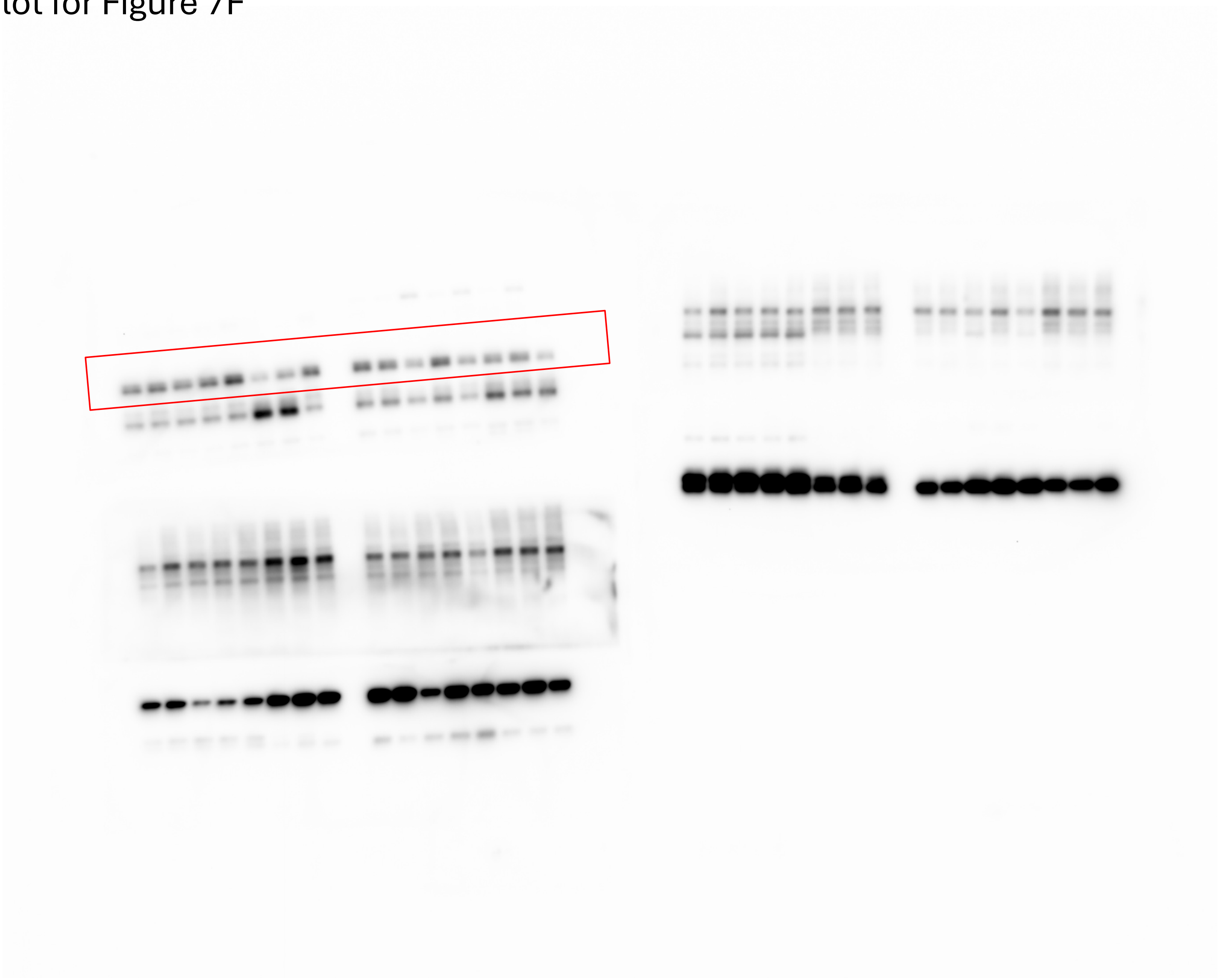

Raw unedited blot for Figure 7F
VDAC1

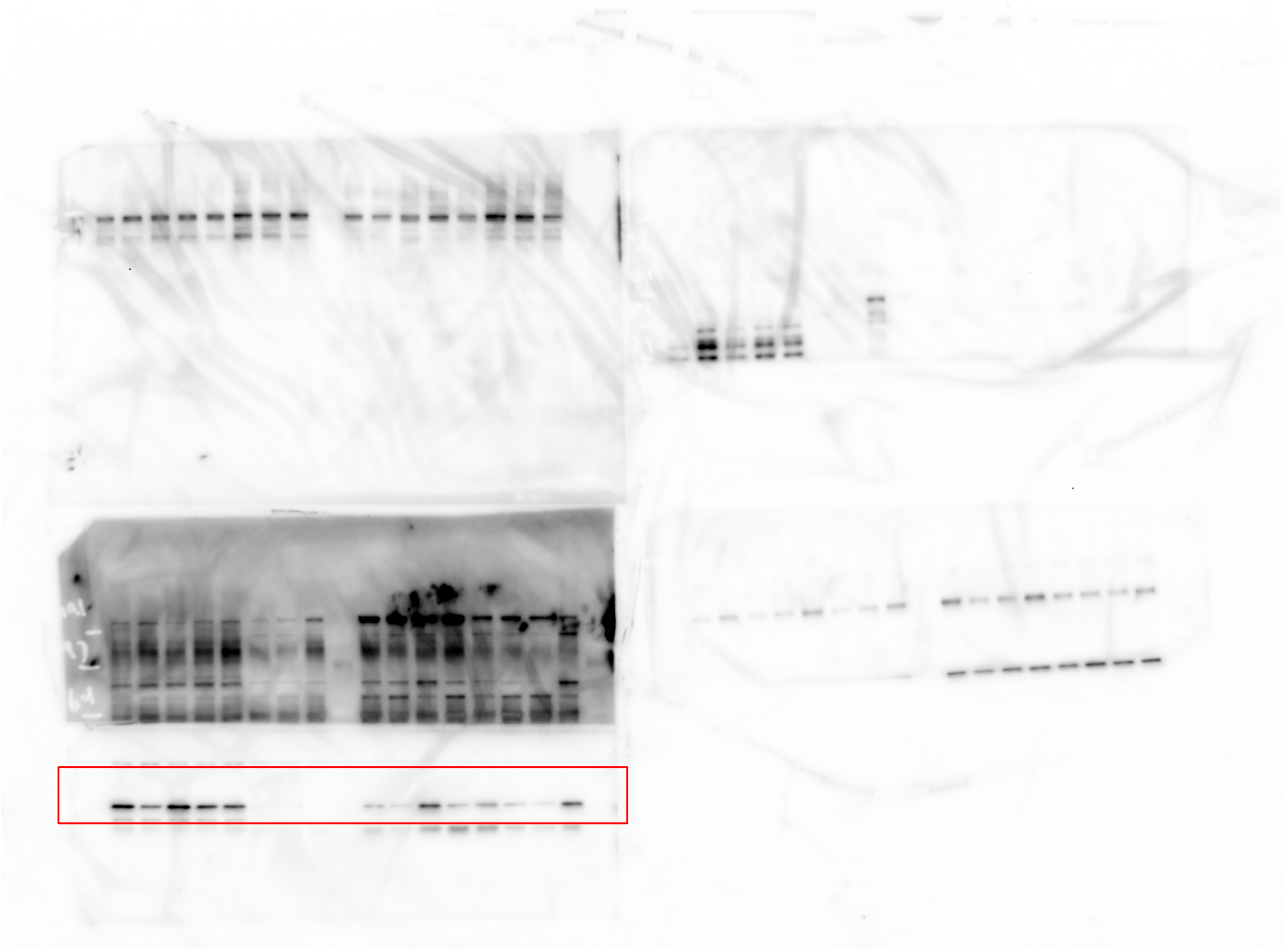

Raw unedited blot for Figure 7F
PDGFRB

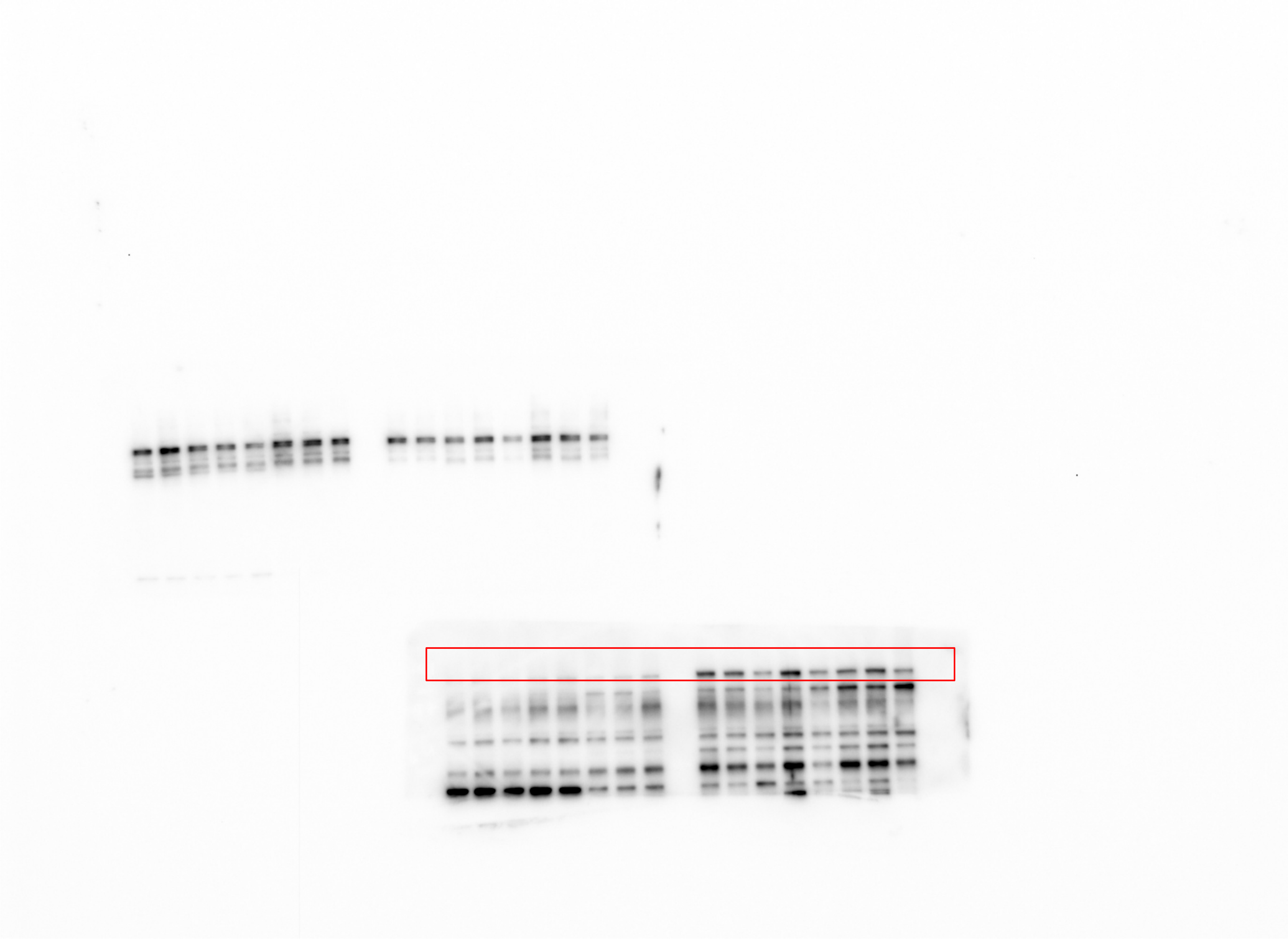

Raw unedited blot for Figure 7F
SCD1

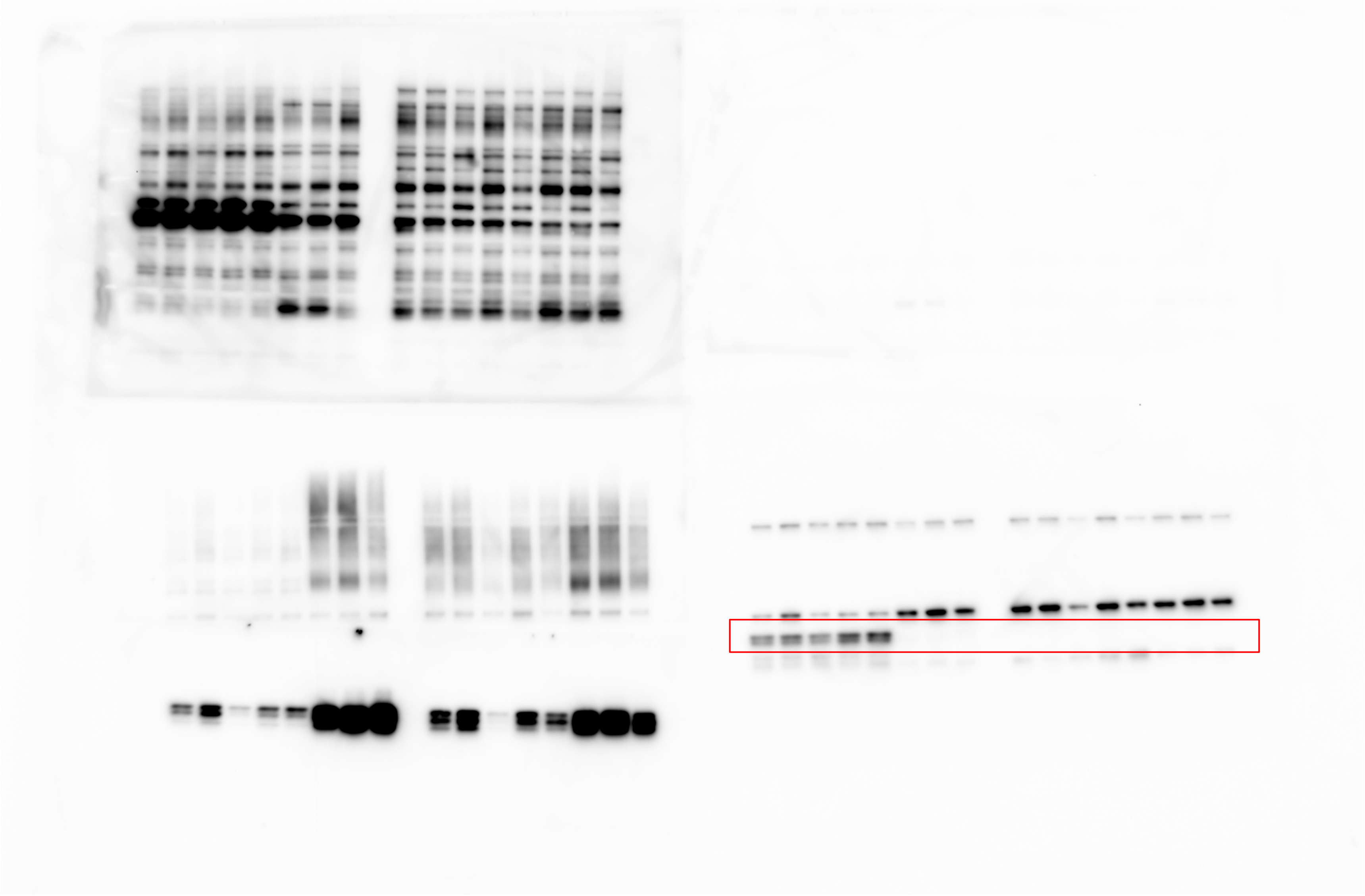

Raw unedited blot for Figure 7F
Adiponectin

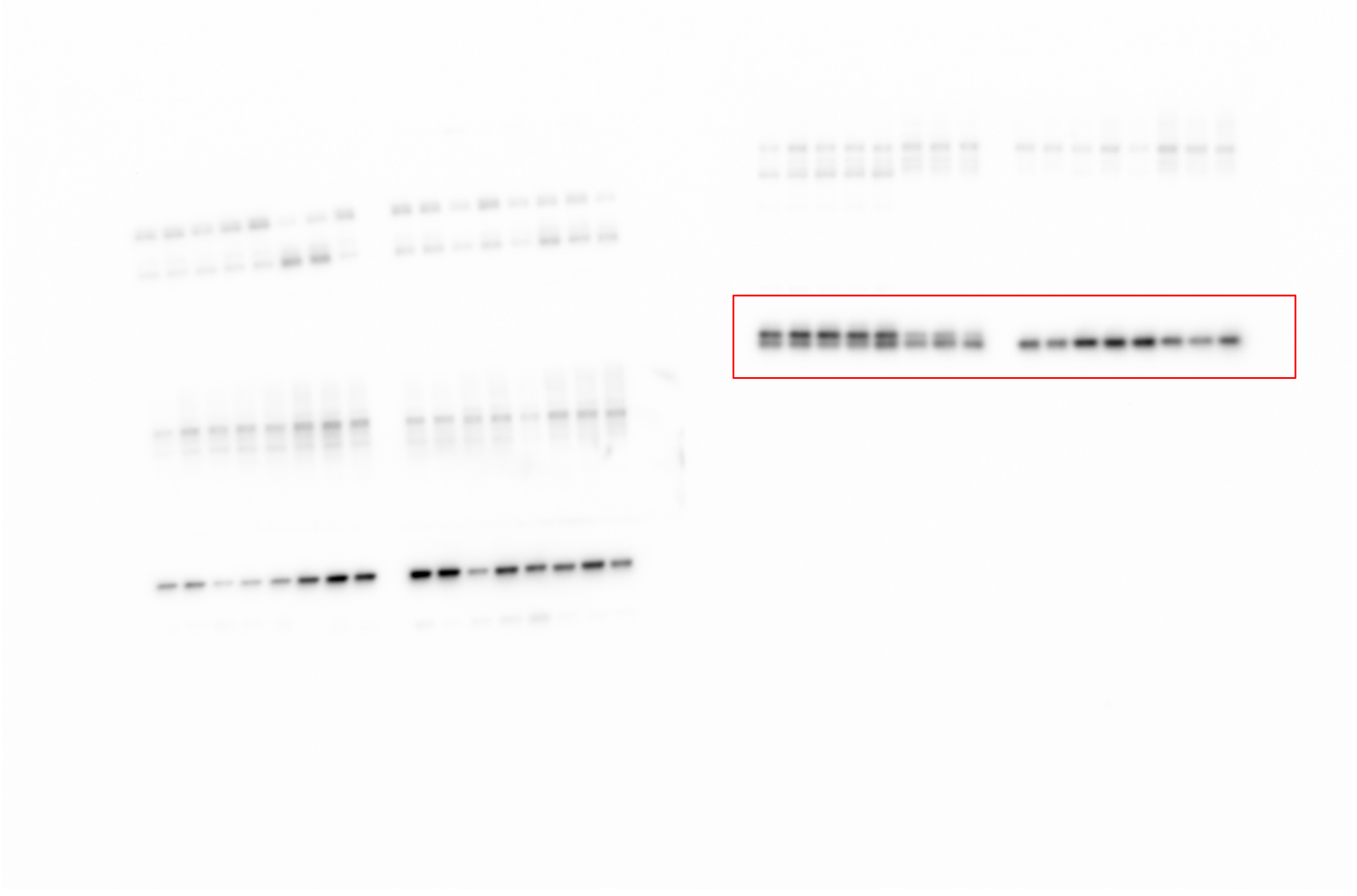

Raw unedited blot for Figure 7F
B-actin

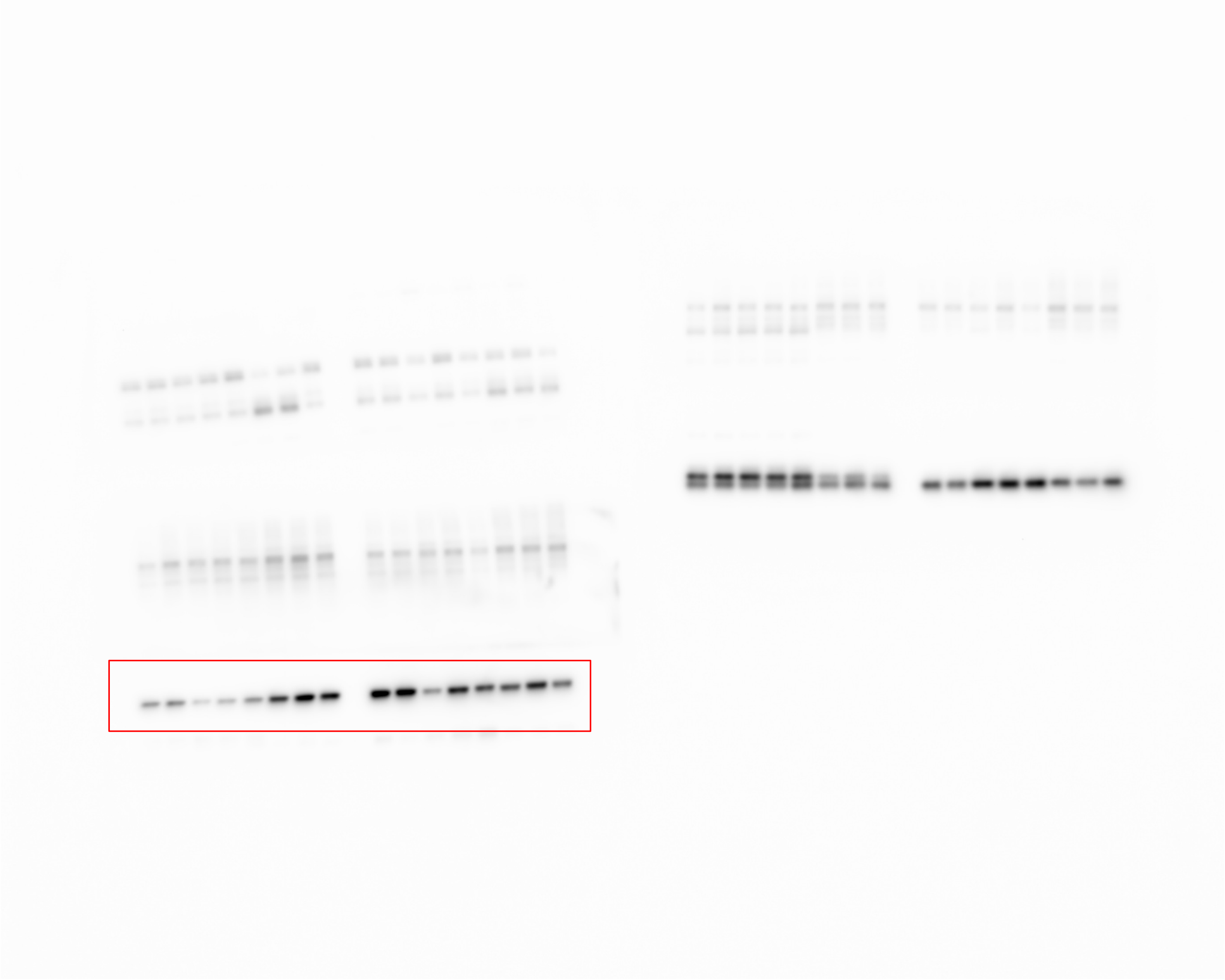

Raw unedited blot for Figure 7F
Laminin

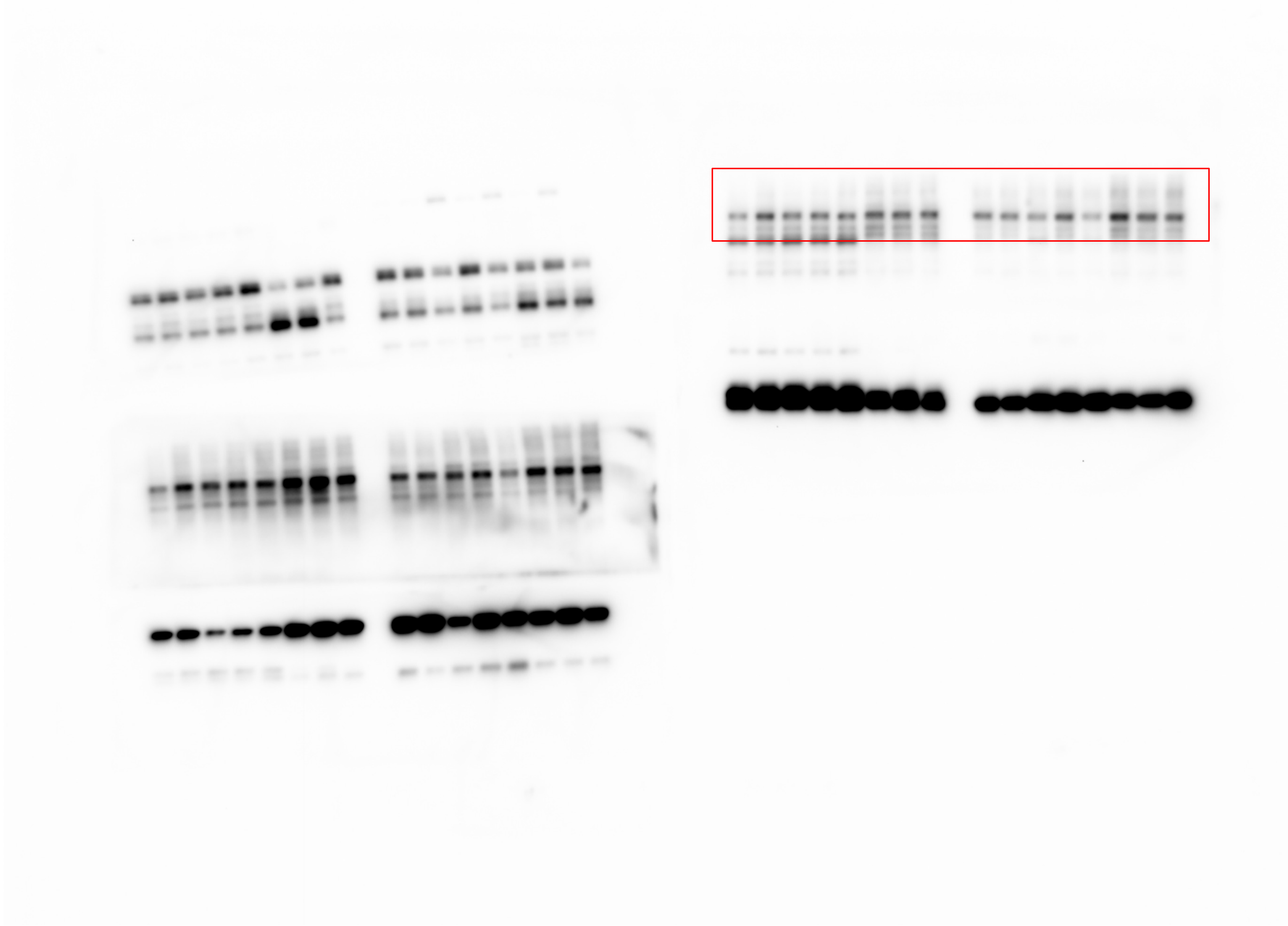

Raw unedited blot for Supp Fig 10U
Cleaved caspase 3

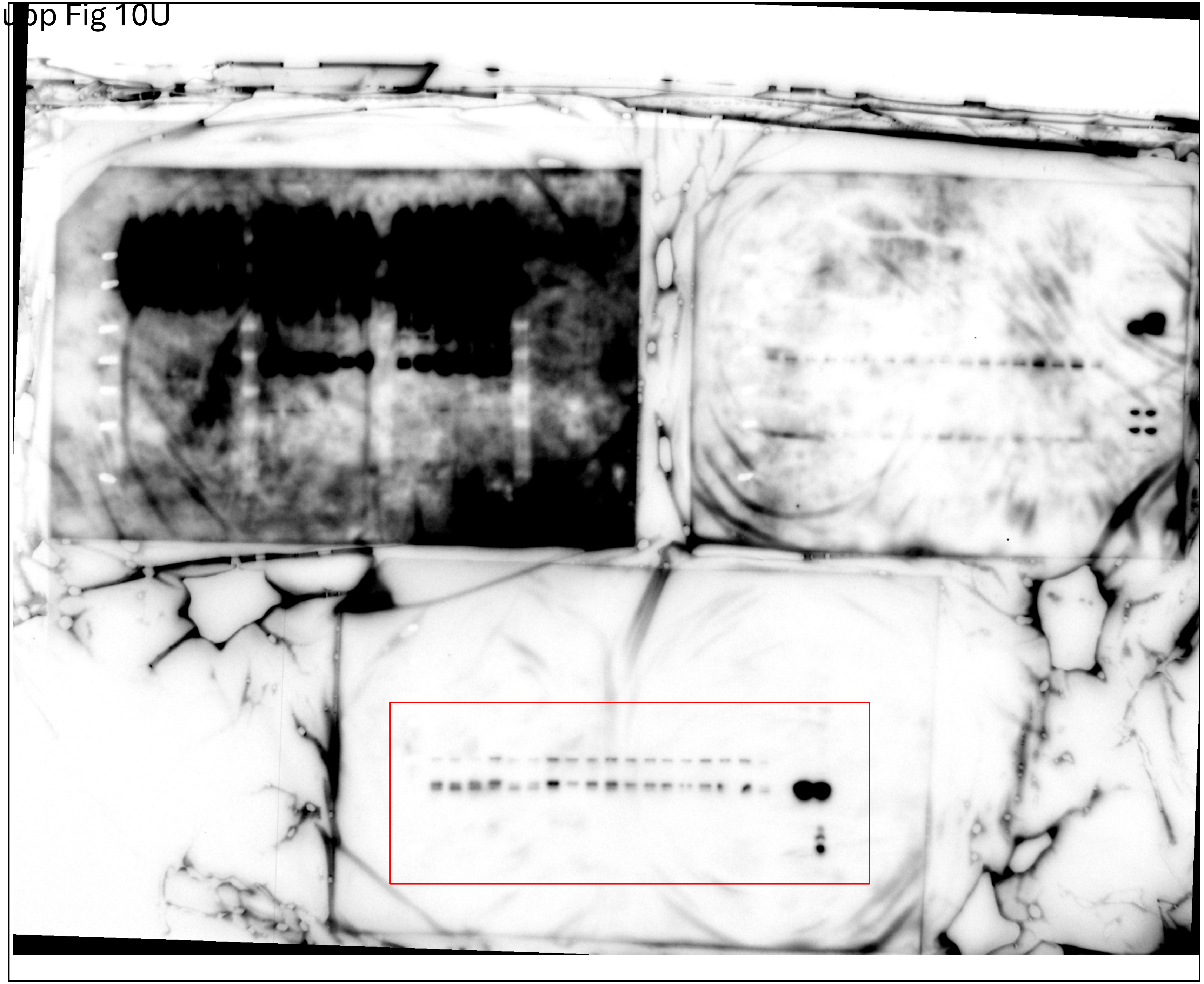

Raw unedited blot for Supp Fig 10U
Laminin

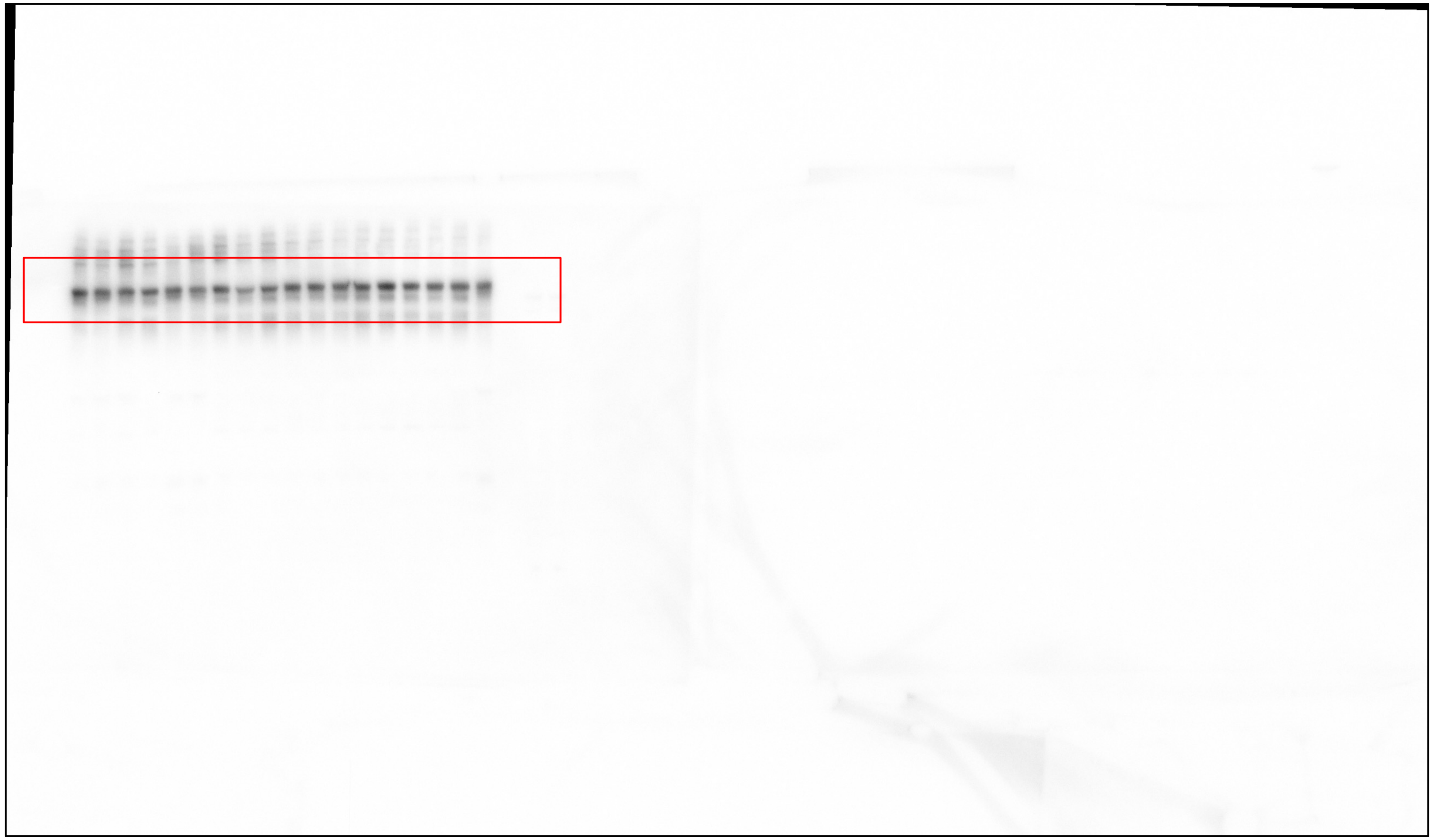

Raw unedited blot for Supp Fig 10V
TBK

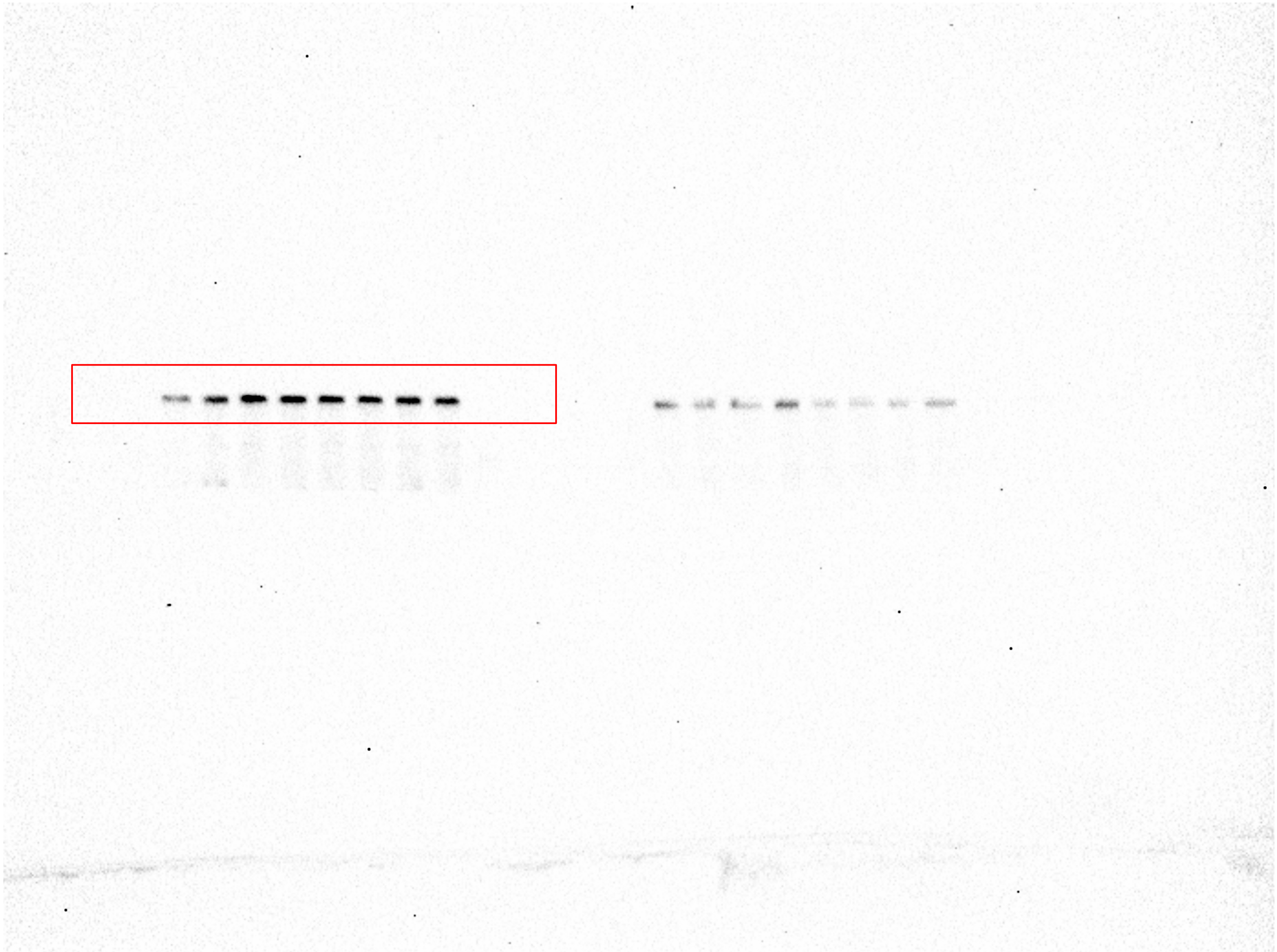

Raw unedited blot for Supp Fig 10V
STING

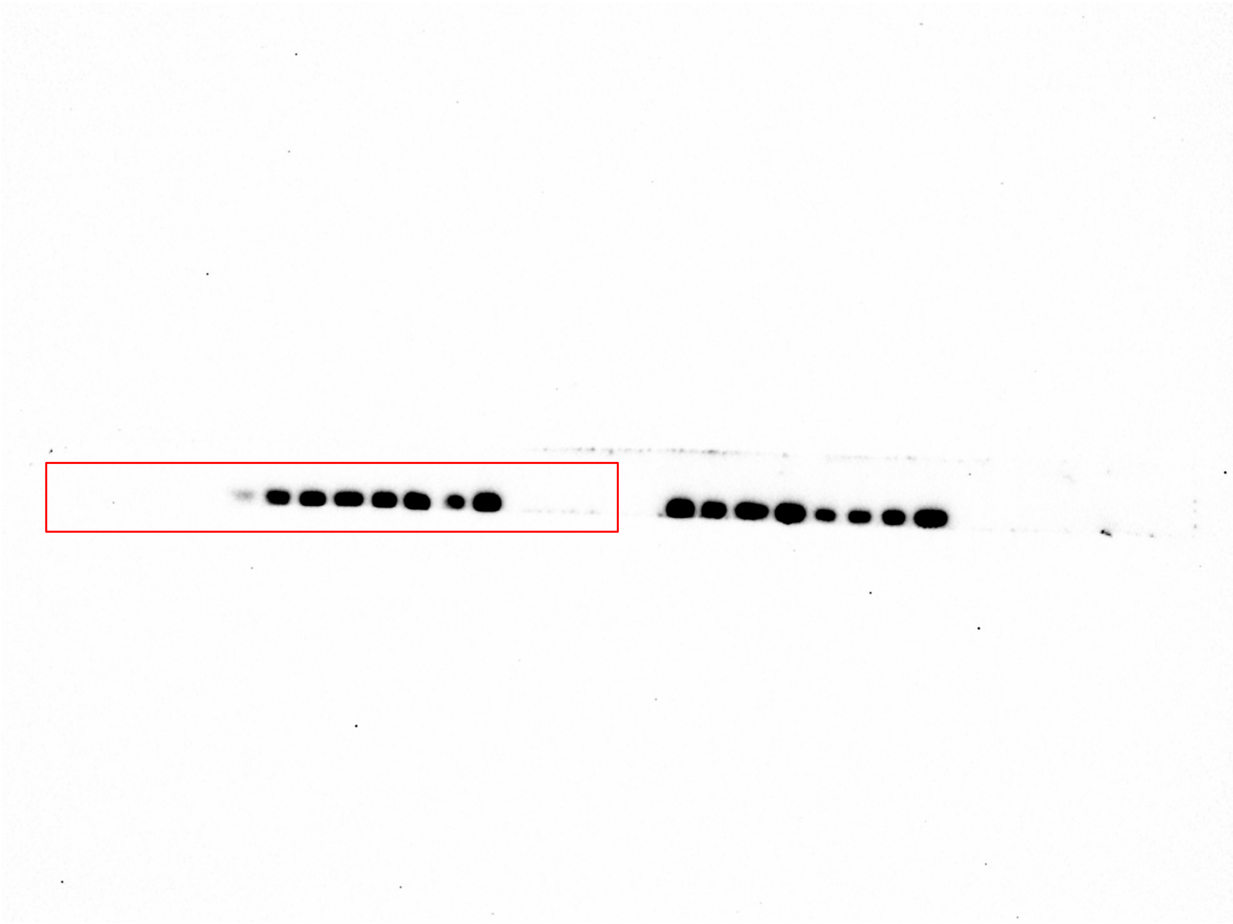

Raw unedited blot for Supp Fig 10V
cGAS

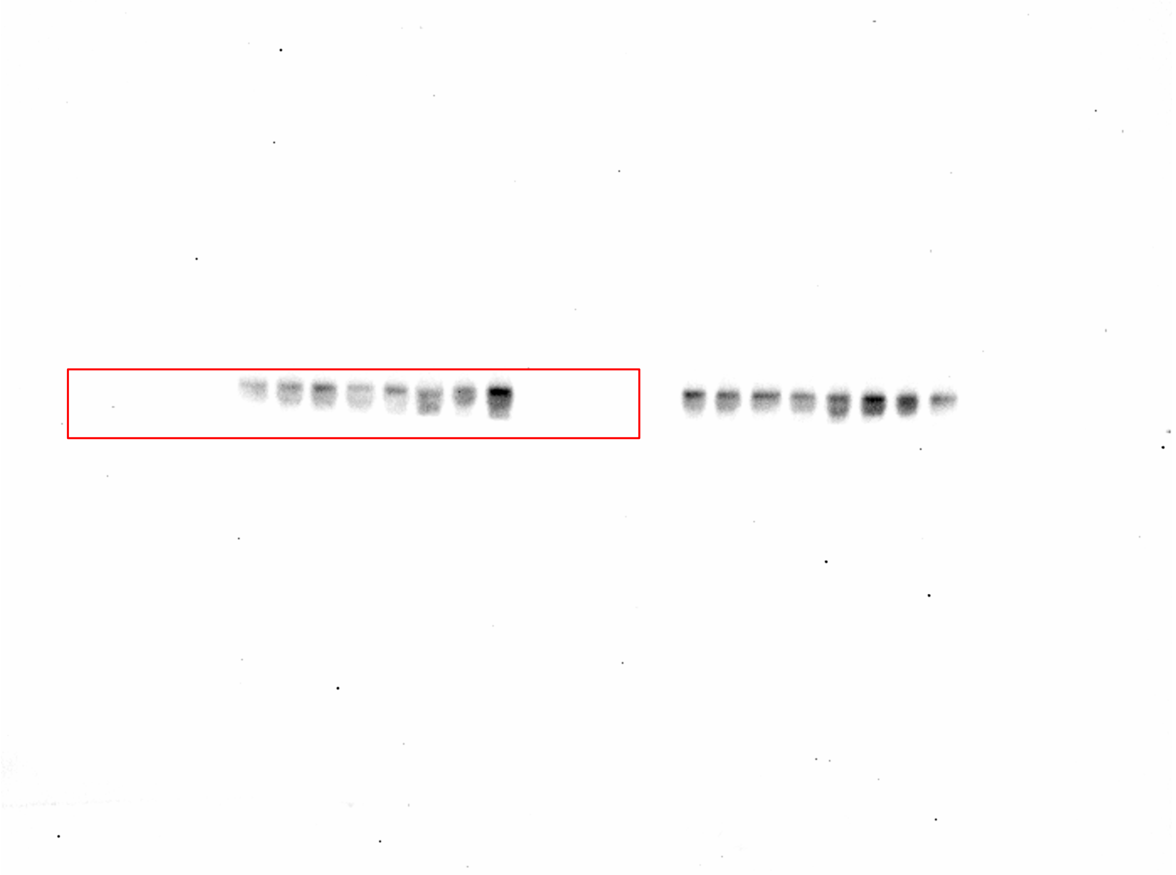

Raw unedited blot for Supp Fig 10V
Laminin

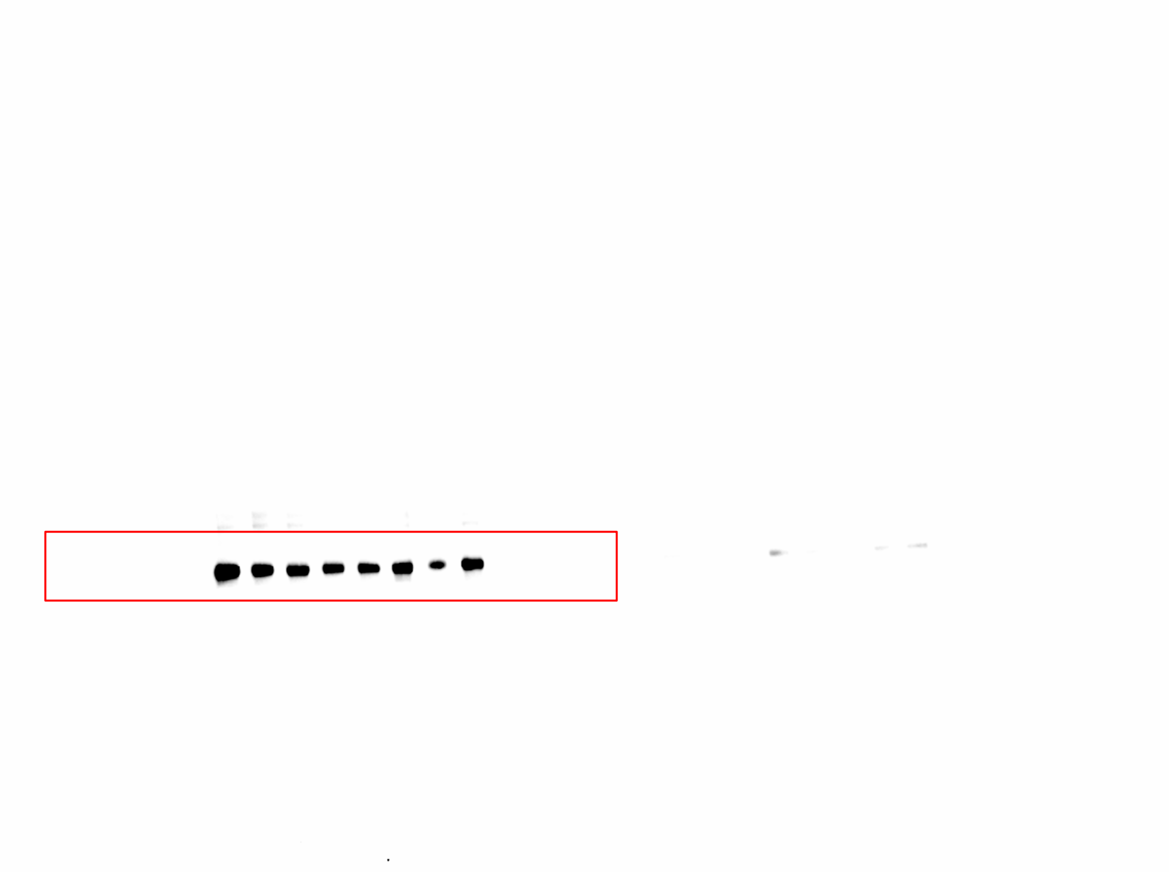

Supplement: Unedited blot and gel images [file jci-136-198387-s288.pdf]
